# Next generation maleimides enable the controlled assembly of antibody–drug conjugates *via* native disulfide bond bridging†
†Electronic supplementary information (ESI) available: Synthetic procedures, ^1^H and ^13^C NMR spectra for synthesised compounds, additional SDS-PAGE gels, LCMS chromatograms, Ellman's analysis and other supplementary results. See DOI: 10.1039/c4ob01550a



**DOI:** 10.1039/c4ob01550a

**Published:** 2014-08-08

**Authors:** Felix F. Schumacher, João P. M. Nunes, Antoine Maruani, Vijay Chudasama, Mark E. B. Smith, Kerry A. Chester, James R. Baker, Stephen Caddick

**Affiliations:** a Department of Chemistry , University College London , 20 Gordon Street , London , WC1H 0AJ , UK . Email: s.caddick@ucl.ac.uk ; Email: j.r.baker@ucl.ac.uk ; Tel: +44 (0)20 3108 7538; b UCL Cancer Institute , 72 Huntley Street , London , WC1E 6BT , UK

## Abstract

The advent of Adcetris™ and Kadcyla™, two recently FDA-approved antibody–drug conjugates (ADCs), in the clinic has had a major impact on the treatment of lymphoma and breast cancer patients, respectively, worldwide. Despite these successes many new ADCs fail at various stages of development, often due to shortcomings in the methods used for their assembly. To address this problem we have developed next generation maleimides (NGMs), which specifically re-bridge reduced interchain disulfide bonds and allow the efficient conjugation of small molecules to antibodies, without the need for engineering of the target antibody. The method is site-specific and generates near homogeneous products in good yields. Moreover, adjustment of the reaction conditions allows control of the conjugation in terms of stoichiometry (drug-loading) and site selectivity. Using this method we prepared a series of ADCs from trastuzumab and doxorubicin (DOX) with a controlled drug-to-antibody ratio (DAR) of 1, 2, 3 and 4. All of these constructs were fully active by ELISA and had more than 90% of re-bridged disulfide bonds by CE-SDS when compared to clinical grade antibody. Furthermore, digest experiments of the DAR 2 material revealed that almost all of the drug had been targeted to the Fab arms of the antibody. Thus, NGMs offer a flexible and simple platform for the controlled assembly of ADCs from an antibody.

## Abstract 2

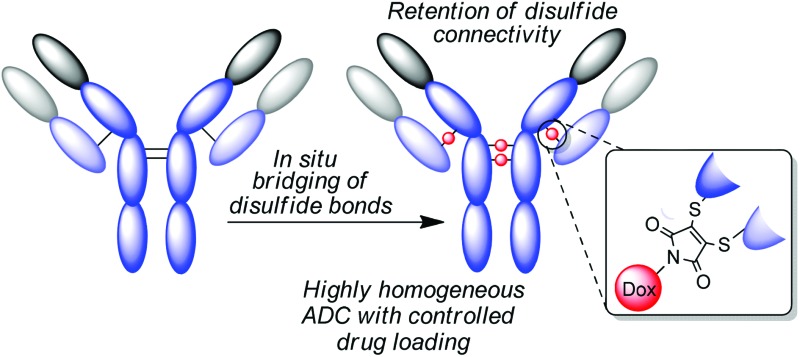
Highly homogeneous ADCs are generated by the efficient bridging of interchain disulfide bonds in trastuzumab, using next generation maleimides.

## Introduction

From all drug classes currently under development, antibody–drug conjugates (ADCs) come closest to Paul Ehrlich's late 19^th^ century vision of the perfect medicine, the “magic bullet”.^1^ The specific binding capability of an antibody enables delivery of an attached cytotoxic drug (payload) to a specific site, which in principle restricts undesirable off-site toxicity effects.^2^ Despite being a relatively simple hypothesis, the development of ADCs is marked with many failures.^3^ Only three molecules of this drug class have received regulatory approval to date, one of which has been voluntarily withdrawn from the market due to a lack of efficacy.^3^

One of the major issues for ADCs is the limited amount of cytotoxic compound that ultimately reaches the tumor.^4^ As a consequence ADCs typically incorporate very potent toxins, such as tubulin inhibitors monomethyl auristatins E^5–8^ and F^9,10^ or the novel DNA intercalators pyrrolobenzodiazepines.^11^ The nature of these toxins necessitates the use of robust linkages to the antibody, which is inherently reliant on the underlying conjugation strategy. Of particular importance is the selectivity of the conjugation method employed – non-selective methods will lead to a heterogeneous mixture of ADC species which^12^ will have different binding activities, potencies, therapeutic indexes, clearance rates and other properties.^13–16^ Homogeneous ADCs are also desirable from a production point of view, as heterogeneity places a large burden on purification, analytics and quality control^17^ to ensure patient safety, which may become an important consideration for regulatory stakeholders.^18^

Developing conjugation methods to deliver homogeneous products can in principle be achieved if the reaction is selective for particular conjugation sites and progresses to completion. In addition, it is important that the conjugation linkage is stable for storage and use *in vivo*. The conjugation methods currently used to assemble ADCs are sub-optimal. Kadcyla™ is produced *via* lysine conjugation,^19,20^ which, with as many as 20 solvent accessible lysines in a typical antibody, is not site-specific and thus cannot be allowed to go to completion. An alternative strategy used for the synthesis of Adcetris™ relies on partial reduction of interchain disulfide bonds followed by alkylation.^6,21^ In this case, the number of conjugation sites differs due to the non-selective nature of the reduction step, thus resulting in a variety of drug-to-antibody (DAR) conjugation ratios. Furthermore the loss of the disulfide bonds introduces stability issues *in vivo*, as does the use of traditional maleimide reagents for conjugation.^22–25^

A number of innovative solutions have been developed to enable the synthesis of highly homogeneous products. “Thiomabs”, which yield ADCs with a DAR close to 2, are based on the insertion of additional free cysteines into the amino acid sequence of the antibody.^15,24^ In a similar fashion, the expression of antibodies containing non-natural amino acids that can be targeted to install small molecules *via* “click chemistry” has been established.^26^ Another alternative involves insertion of small amino acid sequences into the antibody, which can then be used for conjugation using enzymatic methods.^27^ Common to all of these methods is their dependence on protein-engineering to insert additional reactive groups for conjugation. Whilst technically feasible this adds an additional layer of complexity to the production process of ADCs, requiring detailed optimisation for determining suitable insertion sites and introducing potential challenges for scale-up and reproducibility.

**Fig. 1:**
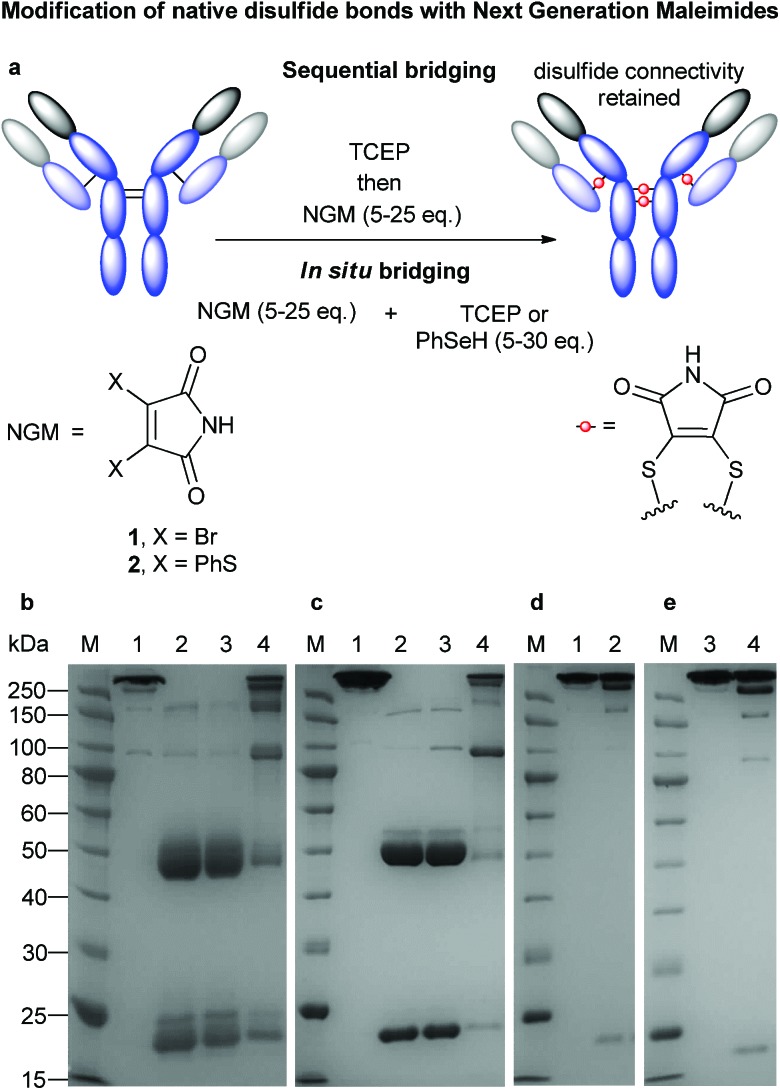
Bridging of native antibody disulfide bonds with NGMs. (a) Sequential and *in situ* functionalisation of antibodies with NGMs (b) Initial sequential modification. (M) Molecular marker. (1) Untreated antibody. (2) Reduced antibody. (3) Antibody reduced in the presence of DMF. (4) Reduction with 20 eq. TCEP, followed by addition of 25 eq. dibromomaleimide. (c) Optimized sequential modification. (M) Molecular marker. (1) Untreated antibody. (2) Reduced antibody. (3) Antibody reduced in the presence of DMF. (4) Reduction with 6 eq. TCEP in pH 8.0 for 2 h at 37 °C followed by addition of 5 eq. dibromomaleimide in 10% final DMF for 1 h. (d) Selenol-based *in situ* modification. (M) Molecular marker. (1) Untreated antibody. (2) Bridging with 10 eq. benzeneselenol and 20 eq. dithiophenolmaleimide in pH 8.0, 15% DMF on ice for 2 h. (e) TCEP-based *in situ* modification. (M) Molecular marker. (3) Untreated antibody. (4) Bridging with 7 eq. TCEP and 7 eq. dithiophenolmaleimide in pH 8.0, 10% DMF at 37 °C for 2 h.

To avoid these issues we have developed reagents (next generation maleimides, NGMs) for site-selective conjugation in a controlled manner, using a feature native to all antibody sub-classes: their interchain disulfide bonds. NGMs are a new class of maleimides, which are substituted in the 3- and 4-position with good leaving groups enabling reaction with two nucleophilic thiol groups such as the two cysteines of a reduced cystine.^28^ This chemical process inserts a 2-carbon bridge into a disulfide bond keeping this structural feature intact whilst functionalizing the protein of interest. The reaction is fast, efficient, high yielding and generates stable products that retain their full biological activity.^29^ Therefore, NGMs fulfill the theoretical criteria for the synthesis of homogeneous conjugation products. Herein we show how this class of compound can be used in the production of near homogeneous ADCs without the need for antibody-engineering (Fig. 1a).

## Results and discussion

### Bridging the interchain disulfide bonds of trastuzumab

To explore the suitability of NGMs in the context of the preparation of ADCs we first needed to establish if efficient re-bridging of the multiple disulfide bonds of a full-length antibody was feasible. We selected clinically validated drug trastuzumab (Herceptin™), a monoclonal IgG1 antibody that targets the internalising HER2 receptor, which is in wide use for the treatment of HER2 positive breast cancer.^30^ It is also the antibody component of ADC trastuzumab emtansine (Kadcyla™).^19^ Initially, all four solvent accessible disulfide bonds of trastuzumab were reduced in PBS buffer with a large excess of reducing agent (20 eq. TCEP, Fig. 1b, lane 3) followed by the addition of dibromomaleimide **1** (25 eq.) without any payload attached. Our initial results were promising as SDS-PAGE revealed formation of fully bridged antibody (Fig. 1b, lane 4), however, significant fragmentation was also observed. It should be noted that this was visible only when samples were heat-treated before the run to ensure full dissociation of heavy and light chains (HCs and LCs) not connected by an intact interchain bridge. No free thiol group was detected by an Ellman's test in the purified product, indicating that NGMs do react very efficiently. However, the method clearly needed further optimisation. To this end, a screen of conditions was carried out, focusing on the following parameters: pH, time, temperature, organic solvent and concentration (Fig. S1, ESI†). Combining the findings of these experiments in an improved re-bridging protocol yielded promising results (Fig. 1c).

Using a revised protocol, the dissociation of the peptide chains was greatly reduced leading to the isolation of two products, completely re-bridged antibody (*i.e.* LHHL) and half-antibody (*i.e.* HL).

We speculate that the formation of this second, half-antibody species can be explained by formation of intrachain bridges. This is possible because the cysteines that form the two disulfide bonds connecting the HCs in the so-called hinge region are separated by only two amino acids. When completely reduced and then re-bridged these can either form the original interchain connections giving full antibody, or make intrachain bridges to produce half-antibody by prohibiting covalent connection of the HCs permanently.

In order to overcome the intrinsic challenge associated with improving hinge region connectivity, we explored the use of an *in situ* method, which we reasoned would avoid disulfide bond shuffling by facilitating immediate re-connection of any opened cystines.^28^ After some experimentation we discovered that using dithiophenolmaleimide **2** in tandem with either TCEP or benzeneselenol (Fig. S2, ESI†) could help mitigate fragment formation as confirmed by SDS-PAGE (Fig. 1d and e). Most notably, we found that *in situ* modification of trastuzumab with the selenol reagent was best performed at 0–4 °C whilst the ideal reaction temperature when using TCEP was 37 °C.

### Synthesis of NGM-doxorubicin

**Fig. 2:**
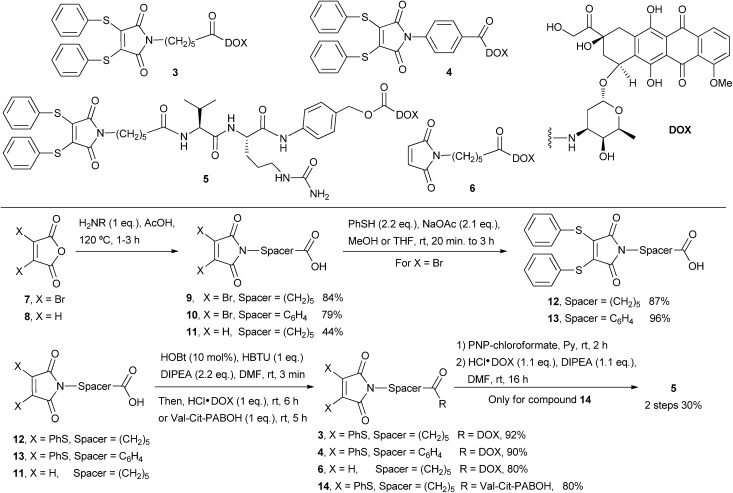
Synthesis of NGM-DOX **3–5** and M-DOX **6**.

We then explored the possibility of using NGMs for conjugation of full antibodies with cytotoxic drugs. We selected anti-cancer drug doxorubicin (DOX) as a warhead, given its prior use as a model payload in ADCs and its convenient UV-vis absorption profile that would facilitate DAR analysis.^12,31,32^ We prepared NGM-DOX compounds **3** and **5**, bearing a C6 alkyl chain. Compound **5** also possesses the valine-citrulline linker for drug release by lysosomal cathepsin B cleavage, which is used in several ADCs.^6,15,31,33–35^ Compound **4** was synthesized to allow evaluation of maleamic acid conjugates.^36^ The preparation of M-DOX **6** enabled comparison with existing technologies (Fig. 2).

The preparation of these compounds started with condensation of 3,4-dibromomaleic anhydride and the chosen amine in acetic acid in accordance with the literature.^37^ This was followed by addition of thiophenol to afford precursors **12** and **13** under efficient and mild conditions.^28^ DOX was attached using HBTU promoted amide bond formation to afford NGM-DOX **3** and **4**.

It is notable that whilst dibromomaleimides can be prone to side reactions in couplings with amines, reaction was facile and high yielding for dithiomaleimides. Since dithiophenolmaleimides are just as effective as dibromomaleimides for cysteine conjugation and allow for *in situ* methods, we focused exclusively on NGM-DOX compounds **3–5** herein. M-DOX **6** was also prepared by the described methods. The valine-citrulline *p*-aminobenzyl alcohol linker (Val-Cit-PABOH) was synthesised as reported^31^ and coupled to compound **12** in high yield *via* HBTU promoted amide bond formation. This was followed by reaction with *p*-nitrophenyl chloroformate and subsequent addition of DOX to afford NGM-DOX **5** (Fig. 2).

### Assembly of trastuzumab–doxorubicin conjugates with NGMs

**Fig. 3:**
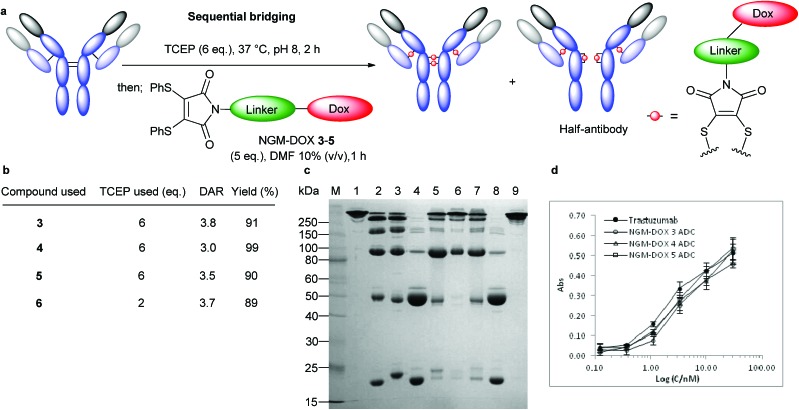
Bridging of native antibody disulfide bonds *via* optimised sequential method. (a) General outline of conjugation with NGM-DOX **3–5**, compared to conjugation with M-DOX **6**. (b) Isolated yields and DAR from conjugation reactions. (c) Analysis by SDS-PAGE: (M) Molecular marker. (1) Untreated antibody. (2) Reduced antibody with 2 eq. TCEP. (3) Conjugation with 5 eq. M-DOX **6**. (4) Reduced antibody with 6 eq. TCEP. (5) Conjugation with 5 eq. NGM-DOX **3**. (6) Conjugation with 5 eq. NGM-DOX **4**. (7) Conjugation with 5 eq. NGM-DOX **5**. (8) Reduced antibody with 6 eq. TCEP in the presence of DMF 10% without NGM. (9) Unreduced antibody with DMF 10% and NGM-DOX **3** (DAR 0). (d) Binding activity of trastuzumab ADCs with NGM-DOX **3–5** to HER2 compared to unmodified trastuzumab.

With NGMs **3–5** in hand we attempted to prepare ADCs with a DAR of 4 by targeting all 4 solvent accessible interchain disulfides in trastuzumab, initially *via* the sequential protocol (Fig. 3a) as described above. Despite the propensity of this protocol to generate some half-antibody, it allowed for direct comparison with alternative contemporary methods of conjugation (*e.g.* with compound **6**). The sequential method using only a slight excess of NGM-DOX reagents **3**, **4** and **5** (5 eq., 1.25 eq. per disulfide bond) provided DARs measured by UV-vis of 3.8, 3.0 and 3.5, respectively (Fig. S3, ESI†), with very high yields of the conjugates obtained after purification (Fig. 3b). Complete cysteine selectivity was confirmed by virtue of the following observations: no conjugation observed between the NGMs and unreduced trastuzumab (Fig. 3c, lane 9); the absence of sulphydryls following conjugation as determined *via* Ellman's analysis (Table S1, ESI†); and no further reaction observed with increased equivalents of NGMs.

The NGM-DOX reagents demonstrated less fragmentation (Fig. 3c, lanes 5, 6 and 7) compared to the use of simple maleimide compound **6**, which afforded all possible fragments (Fig. 3c, lane 3). However, consistent with our expectations, we observed formation of half-antibody (HL) in all cases of conjugation with NGMs **3–5**. Nonetheless, ADCs prepared with NGM-DOX **3–5** did still retain full binding activity to the HER2 antigen, as determined by ELISA (Fig. 3d).

**Fig. 4:**
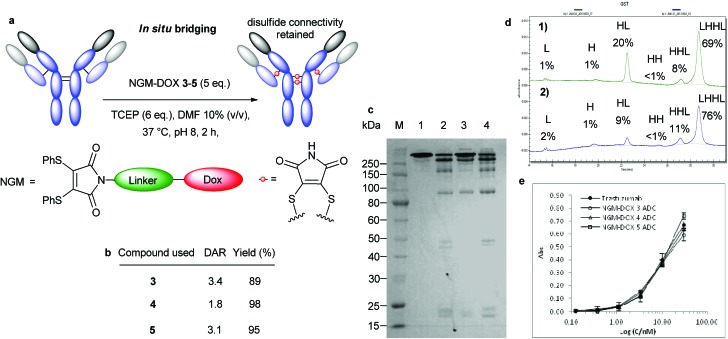
Bridging of native antibody disulfide bonds *via in situ* method. (a) General outline of conjugation with NGM-DOX **3–5**. (b) Isolated yields and DAR from conjugation reactions. (c) Analysis by SDS-PAGE: (M) Molecular marker. (1) Untreated antibody. (2) Conjugation with 5 eq. NGM-DOX **3**. (3) Conjugation with 5 eq. NGM-DOX **4**. (4) Conjugation with 5 eq. NGM-DOX **5**. (d) CGE of conjugation with NGM-DOX **4** by different methods; peak area given as percentage of total peak area: (1) sequential method with 5 eq. of NGM-DOX **4**. (2) *in situ* method with 5 eq. of NGM-DOX **4**. (e) Binding activity of trastuzumab ADCs with NGM-DOX **3–5** to HER2 compared to unmodified trastuzumab.

We next explored the use of *in situ* protocols to avoid the formation of half antibody using NGMs **3–5** and successfully achieved moderate to good DAR values (Fig. S4, ESI†) in high yields (Fig. 4b) and with negligible free sulphydryl groups in the product (Table S2, ESI†). The quantity of half-antibody was dramatically reduced, as indicated by SDS-PAGE and this was confirmed by quantitative capillary gel electrophoresis (CE-SDS) analysis (Fig. 4c and d). The use of NGMs **3** and **5** led to decreased levels of half-antibody and with good DARs. The use of NGM-DOX **4** under *in situ* conditions was optimal, generating conjugates with only minor by-products resulting from fragmentation. This shows that the *in situ* method is capable of affording a full antibody ADC with high selectivity and in good yields. As anticipated, ADCs prepared by the *in situ* protocol displayed excellent retention of binding activity to HER2 (Fig. 4e).

NGM-DOX **4** consistently afforded the lowest DARs, whether generated using the sequential (3.0) or *in situ* (1.8) protocols, and we suspected that this might be a consequence of hydrolysis of the conjugate. NGM-DOX **4** was designed to undergo rapid hydrolysis to generate maleamic acid bridges following conjugation and this was verified by SDS-PAGE (Fig. S5, ESI†).^36^ To explore this further, we modified trastuzumab Fab with NGM-DOX **4** and confirmed full hydrolysis of maleimide to maleamic acid using mass spectrometry (Fig. S6, ESI†). Significantly, mass spectrometry also revealed partial hydrolysis of the acetal moiety in DOX (Table S3, ESI†). The instability of DOX has been documented^38^ and we have previously reported similar observations for another *N*-aryl maleamic acid DOX construct.^39^ Thus, we believe the lower DAR observed with NGM-DOX **4** is more likely to be due to DOX acetal hydrolysis over the extended conjugation time of 2 h rather than a limitation of the conjugation chemistry.

### Controlling drug loading and localization

**Fig. 5:**
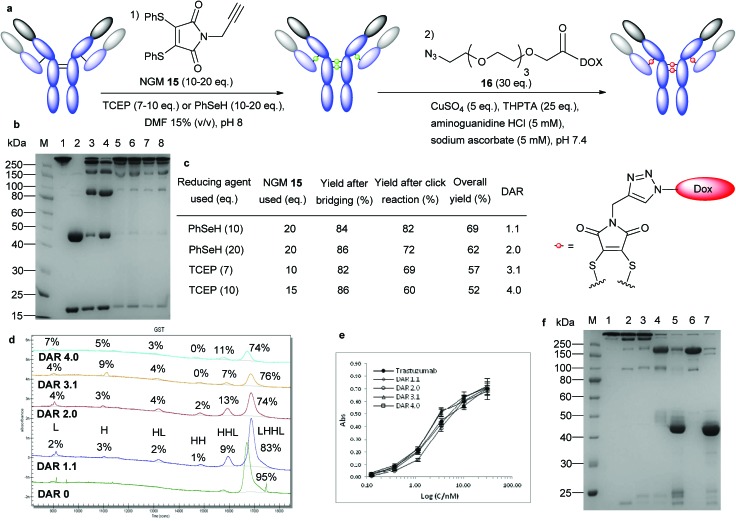
Controlled synthesis of trastuzumab–DOX conjugates and localization of the payload. (a) General outline of the two-step process using *in situ* disulfide bond modification with NGMs followed by copper-catalysed Huisgen 1,3-dipolar cycloaddition. (b) Trastuzumab–DOX DAR series. (M) Molecular marker. (1) Untreated antibody. (2) Reduced antibody. (3) Fragmentation control of trastuzumab prepared by treatment with 2 eq. TCEP (4) Trastuzumab–DOX conjugate prepared with compound **6** (compare Fig. 3c, lane 3). (5) DAR 1.1: reaction on ice for 1 h followed by 90 min click reaction. (6) DAR 2.0: as DAR 1.1 but with addition of 10 eq. benzeneselenol after 30 min and 16 h click reaction. (7) DAR 3.1: reaction at 37 °C for 2 h followed by 90 min click reaction. (8) DAR 4.0: reaction at 37 °C for 2 h followed by 16 h click reaction. (c) Isolated yields and DAR from conjugation reactions, overall yield after SEC purification. (d) CE-SDS analysis of the trastuzumab–DOX DAR series; peak area given as percentage of total peak area. It was assumed for the calculation of correctly bridged disulphide bonds (in contrast to the population of the fragments) that the H and L chains contained 0, the HL fragment 1, the HH fragment 2 and the HHL fragment 3 correct bridges. DAR 0 material was generated by treatment of trastuzumab as outlined for DAR 3.1 material but without addition of reducing agent. (e) Binding activity of the trastuzumab DAR 1 to 4 series to HER2 compared to unmodified trastuzumab. (f) Localization of DOX in DAR 2.0 material. (M) Molecular marker. (1) Untreated antibody. (2) Trastuzumab bridged with compound **15**. (3) Trastuzumab–DOX with a DAR of 2.0 after the click reaction. (4) DAR 2.0 material after pepsin digest. (5) DAR 2.0 material after papain digest. (6) Control digest of unmodified trastuzumab with pepsin. (7) Control digest of pepsin digested trastuzumab with papain.

To avoid problems based on the model payload and to assess the true potential of NGMs in the context of ADC synthesis we decided to examine an alternative approach in which the bridging reaction and the drug-conjugation step could be carried out separately. To this end, we prepared *N*-alkyne-dithiophenolmaleimide **15**,^40^ which would be inserted into the trastuzumab disulfide bonds to provide a bioconjugate ready for further elaboration. We also prepared a DOX-azide species **16** containing a polyethylene glycol (PEG) chain that we envisaged could lead to conjugation *via* a copper catalysed 3 + 2 cycloaddition (Fig. 5a).^41^

Modification of the target antibody with compound **15** *via* TCEP and benzeneselenol protocols proceeded as expected (Fig. S7, ESI†) and yielded the desired intermediate in more than 80% yield (Fig. 5c). Copper-catalysed Huisgen 1,3-dipolar cycloaddition (click reaction) was carried out in the presence of 3[tris(3-hydroxypropyltriazolylmethyl) amine] (THPTA) and aminoguanidine hydrochloride using a seven fold excess of **16** per disulfide bond, which had negligible impact on antibody fragmentation (Fig. S7, ESI†).

The products were purified by size exclusion chromatography (SEC) and we isolated ADCs in 52–69% yield (Fig. 5c). We observed that TCEP-based protocols gave DARs between 3 and 4, whilst methods employing benzeneselenol yielded DARs between 1 and 2 (Fig. S8, ESI†). We explored this observation further in an attempt to develop protocols that would deliver a series of trastuzumab–DOX conjugates with distinct DARs. Careful variation of the reaction conditions, including repeated additions of benzeneselenol, adjusting TCEP stoichiometry and moderating reaction times enabled us to develop protocols for the synthesis of ADCs with DARs of 1.1, 2, 3.1 and 4 (Fig. 5b and c). Furthermore, all molecules isolated contained more than 90% of the connected interchain bridges, at least 74% of intact antibody (Fig. 5d), and retained full binding activity as shown by ELISA (Fig. 5e).

The 4 solvent accessible cystines in IgG1 antibodies can be divided into 2 groups: 2 of them in the hinge region and 2 in the switch/Fab region. The fact that the selenol-based *in situ* protocol did not yield ADCs with a DAR higher than 2 prompted us to explore the possibility that the selenol-based protocol was reacting specifically with the Fab arms.

Proteolytic digestion of the trastuzumab–DOX DAR 2 conjugate derived from the selenol protocol allowed us to isolate the corresponding Fab fragments (Fig. 5f) with a DAR of 0.79 (Fig. S9, ESI†). As direct readout from this process would be misleading due to issues of DOX stability under the reaction conditions, we required an appropriate control/correction factor. To this end, we synthesized a Fab–DOX conjugate *via* the same protocol as established for the full antibody, and this species was subjected to the digest conditions. The loss of 20% of the DOX payload under these conditions, as observed by mass spectrometry and UV-vis spectroscopy (Fig. S10 and S11, ESI†), were used to adjust the results from the full antibody digestion experiment. Taking this into account, the Fab fraction obtained from digestion was found to contain more than 95% of the originally conjugated DOX after enzymatic cleavage. This observation suggests that the combination of NGMs with benzeneselenol can be used to target small molecules exclusively to the Fab arms of antibodies. Additional experimentation will be necessary to understand the chemical and structural basis of this effect.

## Conclusions

ADCs offer a tantalizing possibility for the development of the next generation of therapeutics, but careful consideration needs to be given to each component, including the conjugation method. The methods described in this paper show the possibility for delivering high yields of homogeneous and stable^29^ conjugates using site-specific reactions of native disulfide bonds. It is also possible to offer control over the drug loading in a range between 0 and 4 whilst maintaining the structure and activity of the antibody. The possibility of using the native disulfide bonds of antibodies for bioconjugation offers potential practical advantages over antibody engineering. Moreover the possibility for targeting the Fab arms has not, to the best of our knowledge, been described and may be useful for spatial separation of hydrophobic payloads attached to antibodies. The synthetic methods described herein are practical, efficient and have the potential for further modification and optimisation depending on the antibody and payload under investigation. For these reasons we anticipate that the NGM platform will find considerable utility for the development of future ADCs.

## Materials and methods

### General sequential bridging protocol

To trastuzumab (22.9 μM, ∼300 μL) in borate buffer (25 mM sodium borate, 25 mM NaCl, 1 mM EDTA, pH 8.0) was added TCEP (min. 5 eq., in the same buffer) from a 400–1000× stock solution and the reaction incubated at 37 °C for 2 h under mild agitation. NGMs were prepared in dry DMF (purchased from Sigma Aldrich) as 400–1000× stock solutions and added to reduced trastuzumab (minimum 5 eq.) to achieve a final DMF concentration of 10%. After 1 h at 37 °C excess reagents were removed by ultrafiltration (10 kDa MWCO).

### General *in situ* bridging protocol with TCEP

To trastuzumab (22.9 μM, ∼300 μL) in borate buffer were added NGMs (min. 5 eq. in dry DMF) from 400–1000× stock solutions to achieve a final DMF concentration of 10%. Then TCEP (min. 6 eq.) in borate buffer was added from a 400–1000× stock solution and the reaction incubated at 37 °C for 2 h under mild agitation after which the reaction was purified by ultrafiltration (10 kDa MWCO).

### General *in situ* bridging protocol with benzeneselenol

To trastuzumab (22.9 μM, ∼300 μL) in borate buffer was added DMF to a final concentration of 12% and the mixture put on ice. Next benzeneselenol (min. 10 eq. in dry DMF) was added from a freshly prepared 1000× stock solution followed by addition of NGMs (min. 10 eq. in dry DMF) from 400–1000× stock solutions to achieve a final DMF concentration of 15%. The reaction was kept on ice for 1 h and purified by ultrafiltration (10 kDa MWCO).

### Copper-catalysed Huisgen 1,3-dipolar cycloaddition

Bridging reactions were stopped with 20 eq. of maleimide (in dry DMF) and purified into PBS (pH 7.4) by ultrafiltration (MWCO 10 kDa). After determination of the concentration by UV-vis (*ε*_280_ = 215 380 cm^–1^ M^–1^) and dilution of the antibody to 30 μM, samples were treated with 30 eq. compound **16** in the presence of 150 μM CuSO_4_, 750 μM THPTA, 5 mM aminoguanidine HCl and 5 mM sodium ascorbate. Reactions were incubated at 22 °C for 90 min or 16 h. ADCs were purified by size exclusion chromatography (on a HiLoad Sephadex 75 16/60 column, GE Healthcare, equilibrated in PBS) and the DAR calculated by UV-vis.

### CE-SDS and data analysis

CE-SDS analysis was carried out on a PEREGRINE I machine (deltaDOT). Samples were diluted to 1 mg mL^–1^ in SDS-MW sample buffer (Proteome Lab) and heated to 65 °C for 20 min. 50 μL were transferred into sample vials and loaded into the machine. Separations were performed in a 50 μm diameter fused silica capillary at 22 °C. Separation length was 20.2 cm, run time 45 min and antibody fragments detected at a wavelength of 214 nm. The capillary was flushed with 0.1 M HCl, water and run buffer before sample loading at 5 psi/16 kV. Noise was recorded for 3 min from the run buffer. To verify comparison-based fragment identification a protein sizing standard (Beckman Coulter) was used. Data analysis was carried out with the EVA software (version 3.1.7, deltaDOT). Run files were loaded and analysed with the GST algorithm at a frequency of 40 and a sensitivity of 1. GST peak search was performed between 13 and 32 min (8000 to 20 000 scans) based on the peak identification by mass and comparison between unmodified, partially and fully reduced antibody samples. Peaks corresponding to the LHHL, HHL, HH, HL, H and L antibody species were added manually where necessary and peak area boundaries adjusted for all signals. As the peak area (absorbance) varies depending on the size a correction factor between the absorbance of the full antibody and the completely disassembled antibody (only H and L fragments) was calculated. This factor was adjusted for the area correction of the remaining fragments (HHL, HH, HL) depending on their disulfide bond status, *e.g.* only 25% of the correction factor was applied to the peak area of the HHL fragment as 75% of the disulfide bonds were assumed to be intact. The normalization was established based on the samples of a reduction series and transferred to the samples with varying DARs. For the calculation of the correctly bridged disulfide bonds it was assumed that the H and L fragments contained none, the HL one, the HH two, the HHL three and the full antibody (LHHL) four correct disulfide bonds.

### Digest of DAR 2 material

A Herceptin–DOX conjugate with a DAR of 2.0 was prepared and the pH of the sample lowered *via* a buffer exchange (into 20 mM sodium acetate, pH 3.1) by ultrafiltration (10 kDa MWCO). Immobilized pepsin (0.15 mL) was washed 4× with the same buffer and trastuzumab–DOX (0.45 mL, 3.19 mg mL^–1^) was added. The mixture was incubated for 5 h at 37 °C under constant agitation (1100 rpm). The resin was separated from the digest using a filter column, and washed 3× with digest buffer (50 mM sodium phosphate, 150 mM NaCl, 1 mM EDTA, pH 6.8). The digest was combined with the washes and the volume adjusted to 0.5 mL. Next, immobilised papain (0.5 mL, 0.25 mg mL^–1^) was activated with 10 mM DTT (in digest buffer) under an argon atmosphere and constant agitation (1100 rpm) for 1 h at 25 °C in the dark. The resin was washed 4× with digest buffer (without DTT) and the 0.5 mL of trastuzumab–DOX-Fab_2_ solution was added. The mixture was incubated for 16 h at 37 °C under constant agitation (1100 rpm) in the dark. The resin was separated from the digest using a filter column, washed 3× with PBS (pH 7.0) and the digest combined with the washes. The buffer was exchanged to PBS by ultrafiltration (10 kDa MWCO) and the volume adjusted to 0.3 mL. In parallel, a sample of unmodified Herceptin was processed as a control. Sample and control were analysed by SDS-PAGE and the control by MS as well. The drug loading of the trastuzumab–Fab–DOX was assessed by UV-vis.

## References

[cit1] Strebhardt K., Ullrich A. (2008). Nat. Rev. Cancer.

[cit2] Zhao R. Y., Wilhelm S. D., Audette C., Jones G., Leece B. A., Lazar A. C., Goldmacher V. S., Singh R., Kovtun Y., Widdison W. C., Lambert J. M., Chari R. V. (2011). J. Med. Chem..

[cit3] Beck A., Reichert J. M. (2014). MAbs.

[cit4] Epenetos A. A., Snook D., Durbin H., Johnson P. M., Taylor-Papadimitriou J. (1986). Cancer Res..

[cit5] Alley S. C., Okeley N. M., Senter P. D. (2010). Curr. Opin. Chem. Biol..

[cit6] Doronina S. O., Toki B. E., Torgov M. Y., Mendelsohn B. A., Cerveny C. G., Chace D. F., DeBlanc R. L., Gearing R. P., Bovee T. D., Siegall C. B., Francisco J. A., Wahl A. F., Meyer D. L., Senter P. D. (2003). Nat. Biotechnol..

[cit7] Flygare J. A., Pillow T. H., Aristoff P. (2013). Chem. Biol. Drug Des..

[cit8] Katz J., Janik J. E., Younes A. (2011). Clin. Cancer Res..

[cit9] Doronina S. O., Bovee T. D., Meyer D. W., Miyamoto J. B., Anderson M. E., Morris-Tilden C. A., Senter P. D. (2008). Bioconjugate Chem..

[cit10] Doronina S. O., Mendelsohn B. A., Bovee T. D., Cerveny C. G., Alley S. C., Meyer D. L., Oflazoglu E., Toki B. E., Sanderson R. J., Zabinski R. F., Wahl A. F., Senter P. D. (2006). Bioconjugate Chem..

[cit11] Hartley J. A. (2011). Expert Opin. Invest. Drugs.

[cit12] Willner D., Trail P. A., Hofstead S. J., King H. D., Lasch S. J., Braslawsky G. R., Greenfield R. S., Kaneko T., Firestone R. A. (1993). Bioconjugate Chem..

[cit13] Hamblett K. J., Senter P. D., Chace D. F., Sun M. M. C., Lenox J., Cerveny C. G., Kissler K. M., Bernhardt S. X., Kopcha A. K., Zabinski R. F., Meyer D. L., Francisco J. A. (2004). Clin. Cancer Res..

[cit14] Junutula J. R., Flagella K. M., Graham R. A., Parsons K. L., Ha E., Raab H., Bhakta S., Nguyen T., Dugger D. L., Li G., Mai E., Lewis Phillips G. D., Hiraragi H., Fuji R. N., Tibbitts J., Vandlen R., Spencer S. D., Scheller R. H., Polakis P., Sliwkowski M. X. (2010). Clin. Cancer Res..

[cit15] Junutula J. R., Raab H., Clark S., Bhakta S., Leipold D. D., Weir S., Chen Y., Simpson M., Tsai S. P., Dennis M. S., Lu Y., Meng Y. G., Ng C., Yang J., Lee C. C., Duenas E., Gorrell J., Katta V., Kim A., McDorman K., Flagella K., Venook R., Ross S., Spencer S. D., Lee Wong W., Lowman H. B., Vandlen R., Sliwkowski M. X., Scheller R. H., Polakis P., Mallet W. (2008). Nat. Biotechnol..

[cit16] McDonagh C. F., Turcott E., Westendorf L., Webster J. B., Alley S. C., Kim K., Andreyka J., Stone I., Hamblett K. J., Francisco J. A., Carter P. (2006). Protein Eng., Des. Sel..

[cit17] Wakankar A., Chen Y., Gokarn Y., Jacobson F. S. (2011). MAbs.

[cit18] Panowski S., Bhakta S., Raab H., Polakis P., Junutula J. R. (2014). MAbs.

[cit19] Verma S., Miles D., Gianni L., Krop I. E., Welslau M., Baselga J., Pegram M., Oh D.-Y., Diéras V., Guardino E., Fang L., Lu M. W., Olsen S., Blackwell K. (2012). N. Engl. J. Med..

[cit20] Wakankar A. A., Feeney M. B., Rivera J., Chen Y., Kim M., Sharma V. K., Wang Y. J. (2010). Bioconjugate Chem..

[cit21] Younes A., Bartlett N. L., Leonard J. P., Kennedy D. A., Lynch C. M., Sievers E. L., Forero-Torres A. (2010). N. Engl. J. Med..

[cit22] Adem Y. T., Schwarz K. A., Duenas E., Patapoff T. W., Galush W. J., Esue O. (2014). Bioconjugate Chem..

[cit23] Boswell C. A., Mundo E. E., Zhang C., Bumbaca D., Valle N. R., Kozak K. R., Fourie A., Chuh J., Koppada N., Saad O., Gill H., Shen B. Q., Rubinfeld B., Tibbitts J., Kaur S., Theil F. P., Fielder P. J., Khawli L. A., Lin K. (2011). Bioconjugate Chem..

[cit24] Shen B. Q., Xu K., Liu L., Raab H., Bhakta S., Kenrick M., Parsons-Reponte K. L., Tien J., Yu S. F., Mai E., Li D., Tibbitts J., Baudys J., Saad O. M., Scales S. J., McDonald P. J., Hass P. E., Eigenbrot C., Nguyen T., Solis W. A., Fuji R. N., Flagella K. M., Patel D., Spencer S. D., Khawli L. A., Ebens A., Wong W. L., Vandlen R., Kaur S., Sliwkowski M. X., Scheller R. H., Polakis P., Junutula J. R. (2012). Nat. Biotechnol..

[cit25] Wilbur D. S., Chyan M.-K., Nakamae H., Chen Y., Hamlin D. K., Santos E. B., Kornblit B. T., Sandmaier B. M. (2012). Bioconjugate Chem..

[cit26] Axup J. Y., Bajjuri K. M., Ritland M., Hutchins B. M., Kim C. H., Kazane S. A., Halder R., Forsyth J. S., Santidrian A. F., Stafin K., Lu Y., Tran H., Seller A. J., Biroc S. L., Szydlik A., Pinkstaff J. K., Tian F., Sinha S. C., Felding-Habermann B., Smider V. V., Schultz P. G. (2012). Proc. Natl. Acad. Sci. U. S. A..

[cit27] Rabuka D. (2010). Curr. Opin. Chem. Biol..

[cit28] Schumacher F. F., Nobles M., Ryan C. P., Smith M. E., Tinker A., Caddick S., Baker J. R. (2011). Bioconjugate Chem..

[cit29] Schumacher F. F., Sanchania V. A., Tolner B., Wright Z. V., Ryan C. P., Smith M. E., Ward J. M., Caddick S., Kay C. W., Aeppli G., Chester K. A., Baker J. R. (2013). Sci. Rep..

[cit30] Hudis C. A. (2007). N. Engl. J. Med..

[cit31] Dubowchik G. M., Firestone R. A., Padilla L., Willner D., Hofstead S. J., Mosure K., Knipe J. O., Lasch S. J., Trail P. A. (2002). Bioconjugate Chem..

[cit32] Shouval D., Adler R., Wands J. R., Hurwitz E., Isselbacher K. J., Sela M. (1988). Proc. Natl. Acad. Sci. U. S. A..

[cit33] Bhaskar V., Law D. A., Ibsen E., Breinberg D., Cass K. M., DuBridge R. B., Evangelista F., Henshall S. M., Hevezi P., Miller J. C., Pong M., Powers R., Senter P., Stockett D., Sutherland R. L., von Freeden-Jeffry U., Willhite D., Murray R., Afar D. E. H., Ramakrishnan V. (2003). Cancer Res..

[cit34] Mao W., Luis E., Ross S., Silva J., Tan C., Crowley C., Chui C., Franz G., Senter P., Koeppen H., Polakis P. (2004). Cancer Res..

[cit35] Sanderson R. J., Hering M. A., James S. F., Sun M. M. C., Doronina S. O., Siadak A. W., Senter P. D., Wahl A. F. (2005). Clin. Cancer Res..

[cit36] Ryan C. P., Smith M. E. B., Schumacher F. F., Grohmann D., Papaioannou D., Waksman G., Werner F., Baker J. R., Caddick S. (2011). Chem. Commun..

[cit37] Dubernet M., Caubert V., Guillard J., Viaud-Massuard M. C. (2005). Tetrahedron.

[cit38] Trissel L. A., Handbook on Injectable Drugs, American Society of Health-System Pharmacists, MacMillan Press, Basingstoke, 9th edn, 1996, p. 379.

[cit39] Castañeda L., Maruani A., Schumacher F. F., Miranda E., Chudasama V., Chester K. A., Baker J. R., Smith M. E. B., Caddick S. (2013). Chem. Commun..

[cit40] Castañeda L., Wright Z. V. F., Marculescu C., Tran T. M., Chudasama V., Maruani A., Hull E. A., Nunes J. P. M., Fitzmaurice R. J., Smith M. E. B., Jones L. H., Caddick S., Baker J. R. (2013). Tetrahedron Lett..

[cit41] Agard N. J., Baskin J. M., Prescher J. A., Lo A., Bertozzi C. R. (2006). ACS Chem. Biol..

