# Supplementary material for: Next generation maleimides enable the controlled assembly of antibody–drug conjugates via native disulfide bond bridging
Source: Org Biomol Chem. 2014 Aug 8;12(37):7261–9. doi: 10.1039/c4ob01550a (PMC4159697; doi:10.1039/c4ob01550a)
Supplement: Supplementary file 1 [file OB-012-C4OB01550A-s001.pdf]

This journal is © The Royal Society of Chemistry 2014

Electronic Supplementary Information

Next generation maleimides enable the controlled assembly of antibody-drug conjugates *via* native disulfide bond bridging

Felix F. Schumacher,[a,b] João P. M. Nunes,[a,b] Antoine Maruani,[a] Vijay Chudasama,[a] Mark E. B. Smith,[a] Kerry A. Chester,[c] James R. Baker*[a] and Stephen Caddick*[a]

[a] Department of Chemistry, University College London, 20 Gordon Street, London, WC1H OAJ, UK.
[b] These authors contributed equally to the paper.
[c] UCL Cancer Institute, 72 Huntley Street, London, WC1E 6BT, UK

Correspondence should be addressed to Stephen Caddick (s.caddick@ucl.ac.uk).

Sections Included

Synthesis General Remarks 4
Synthesis of compounds 5
3,4-Dibromo-maleimide-*N*-hexanoic acid **9** 5
N-(*p*-Benzoic acid)-3,4-dibromo-maleimide **10** 7
Maleimide-*N*-hexanoic acid **11** 9
3,4-Dithiophenoyl-maleimide-*N*-hexanoic acid **12** 11
N-(*p*-Benzoic acid)-3,4-dithiophenoyl-maleimide **13** 13
N-(Hexanamide-*N*-doxorubicin)-3,4-dithiophenoyl-maleimide **3** 15
N-(*p*-Benzamide-*N*-doxorubicin)-3,4-dithiophenoyl-maleimide **4** 18
N-(*N*-Doxorubicinhexanamide)-maleimide **6** 20
3,4-Dithiophenoyl-maleimide-*N*-hexanamide-valine-citrulline-*p*-aminobenzyl alcohol **14** 23
N-(Hexanamide-valine-citruline-*p*-aminobenzylcarbonyl-*N*-doxorubicin)-3,4-dithiophenoyl-maleimide **5** 25
N-Propargyl-3,5-dithiophenoyl-maleimide **15** 28
N-Doxorubicin-14-azido-3,6,9,12-tetraoxatetradecan-1-amide **16** 29
Conjugation Experiments General Remarks 32
Analytical methods for antibody-drug conjugates 33
SDS-PAGE 33
Determination of drug to antibody ratio (DAR) 33
Ellman’s assay 34
Liquid chromatography mass spectrometry (LCMS) 34
Enzyme-linked immunosorbent assay (ELISA) 35
Trastuzumab modification protocols 36
Conjugation of trastuzumab with NGM-DOX **3-5** by sequential protocol 36
Conjugation of trastuzumab with M-DOX **6** by sequential protocol 36
Conjugation of trastuzumab with NGM-DOX **3-5** by *in situ* protocol 36
Conjugation of trastuzumab with compound **15** by *in situ* protocols, followed by copper-catalysed Huisgen 1,3-dipolar cycloaddition with compound **16** 37
Supplementary figures and tables 38
Figure S1 – Optimization of the sequential bridging protocol 38
Figure S2 – Optimization of the *in situ* bridging protocol 40
Figure S3 – UV-vis spectra for determination of DAR for ADCs prepared with NGM-DOX **3-5** and M-DOX **6** by sequential protocol 41
Table S1 – Ellman’s analysis after conjugation of trastuzumab with NGM-DOX **3-5** by sequential protocol. 42
Figure S4 – UV-vis spectra for determination of DAR for ADCs prepared with NGM-DOX **3-5** by *in situ* protocol 42
Table S2 – Ellman’s analysis after conjugation of trastuzumab with NGM-DOX **3-5** by *in situ* protocol. 43
Figure S5 – Reduction SDS-PAGE gel of ADCs to determine hydrolysis of maleimide bridges to maleamic acid bridges 43
Figure S6 – MS data of conjugation of trastuzumab Fab with NGM-DOX **4** 45
Table S3 – Comparison of DAR by UV-vis and amount of Fab-DOX by LCMS for conjugation of trastuzumab Fab with NGM-DOX **4**. 47
Figure S7 – Initial bridging with compound **15** and influence of duration of the copper-catalysed Huisgen 1,3-dipolar cycloaddition 47
Figure S8 – UV-vis spectra for determination of DAR for ADCs prepared with compound **15** by *in situ* protocols, followed by copper-catalysed Huisgen 1,3-dipolar cycloaddition with compound **16** 48

Figure S9 – UV-vis spectra for determination of DAR for trastuzumab Fab ADC resulting from proteolytic digest of DAR 2 ADC material 49
Figure S10 – MS data on the control digest of DAR 2 material.. 49
Figure S11 – UV-vis spectra for determination of DAR for the control digest of DAR 2 material 51
References.. 52

Synthesis General Remarks

All reactions were carried out at atmospheric pressure with stirring at 20 °C unless otherwise stated. Reagents and solvents were purchased from Sigma Aldrich and Alfa Aesar and used as supplied. Reactions were monitored by TLC analysis carried out on silica gel SIL G/UV254 coated onto aluminium plates purchased from VWR. Visualization was carried out under a UV lamp operating at 254 nm wavelength and by staining with a solution of potassium permanganate (3 g) and potassium carbonate (20 g) in 5% aqueous sodium hydroxide (5 mL) and water (200 mL), followed by heating. Flash column chromatography was carried out with silica gel 60 (0.04-0.063 mm, 230-400 mesh) purchased from Merck, using solvents dichloromethane (DCM), methanol (MeOH), ethyl acetate (EtOAc) and petroleum ether 40 °C - 60 °C boiling range, purchased from Fisher Scientific. Nuclear magnetic resonance spectra were recorded in either $CDCl_3$, MeOD-d_4 or DMSO-d_6 (unless another solvent is stated) on Bruker NMR spectrometers operating at ambient 20 °C probe. ^{1}H spectra were recorded at 400, 500 or 600 MHz and ^{13}C spectra were recorded at 125 or 150 MHz, using residual solvents as internal reference. Where necessary, DEPT135, COSY, HMQC, HMBC and NOESY spectra have been used to ascertain structure. Data is presented as follows for ^{1}H: chemical shift in ppm (multiplicity, *J* coupling constant in Hz, nº of H, assignment on structure); and on ^{13}C: chemical shift in ppm (assignment on structure). Multiplicity is reported as follows: s (singlet), d (doublet), t (triplet), q (quartet), quint. (quintet), sext. (sextet), oct. (octet), m (multiplet), br (broad), dd (doublet of doublet), dt (doublet of triplets), ABq (AB quartet). Infrared spectra were recorded on a Perkin Elmer Spectrum 100 FTIR spectrometer operating in ATR mode. Melting points were measured on a Gallenkamp apparatus and are uncorrected. Experimental procedures for all isolated compounds are presented. All yields quoted are isolated yields, unless otherwise stated, and when multiple products are obtained, data are presented in terms of order isolated. Dibromomaleimide **1** was purchased from Sigma Aldrich and dithiophenolmaleimide **2** was prepared as reported by Schumacher *et al.*[1]

Synthesis of compounds

3,4-Dibromo-maleimide-*N*-hexanoic acid 9

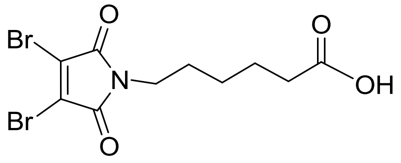

In a 10 mL round-bottom flask, 3,4-dibromomaleic anhydride (256 mg, 1.0 mmol), prepared as reported,[2] and 6-aminocaproic acid (131 mg, 1.0 mmol, 1 eq.) were added. Next, AcOH (2 mL) was added and the mixture was heated at 120 °C with stirring for 3 h. Then, the mixture was allowed to cool to 20 °C. AcOH was removed by concentrating under vacuum at 80 °C and traces of AcOH were removed by adding toluene (10 mL) and concentrating once more to yield a yellow white solid which was purified by flash chromatography on silica with petroleum ether:EtOAc (1:1 v/v) to afford **9** as a white solid (311 mg, 0.84 mmol, 84%). Data for **9**: mp = 123-124 °C. IR (pellet) ν_{max} 2936, 2868, 1721, 1695, 1589, 1396, 1046, 946, 842, 733. ^{1}H NMR (500 MHz, MeOD-d$_4$) 1.34 (quint., J = 7.5 Hz, 2H, CH_2), 1.63 (overlapped quint., J = 7.5 Hz, 4H, CH_2), 2.29 (t, J = 7.5 Hz, 2H, CH_2), 3.58 (t, J = 7.5 Hz, 2H, CH_2); ^{13}C NMR (125 MHz, MeOD-d4) 25.5 (CH_2), 27.2 (CH_2), 29.0 (CH_2), 34.6 (CH_2), 40.3 (CH_2), 130.3 (C), 165.5 (CO), 177.4 (CO). LRMS (ESI) 286 (100, [M^{79}Br-Br]$^+$), 288 (100, [M^{81}Br-Br]$^+$), 366 (20, [M^{79}Br^{79}Br]$^+$), 368 (40, [M^{81}Br^{79}Br]$^+$), 370 (20, [M^{81}Br^{81}Br]$^+$); HRMS (ESI) calcd. for $C_{10}H_{10}NO_4Br_2$ [M^{79}Br^{79}Br-H]$^+$ 365.8977, observed: 365.8986.

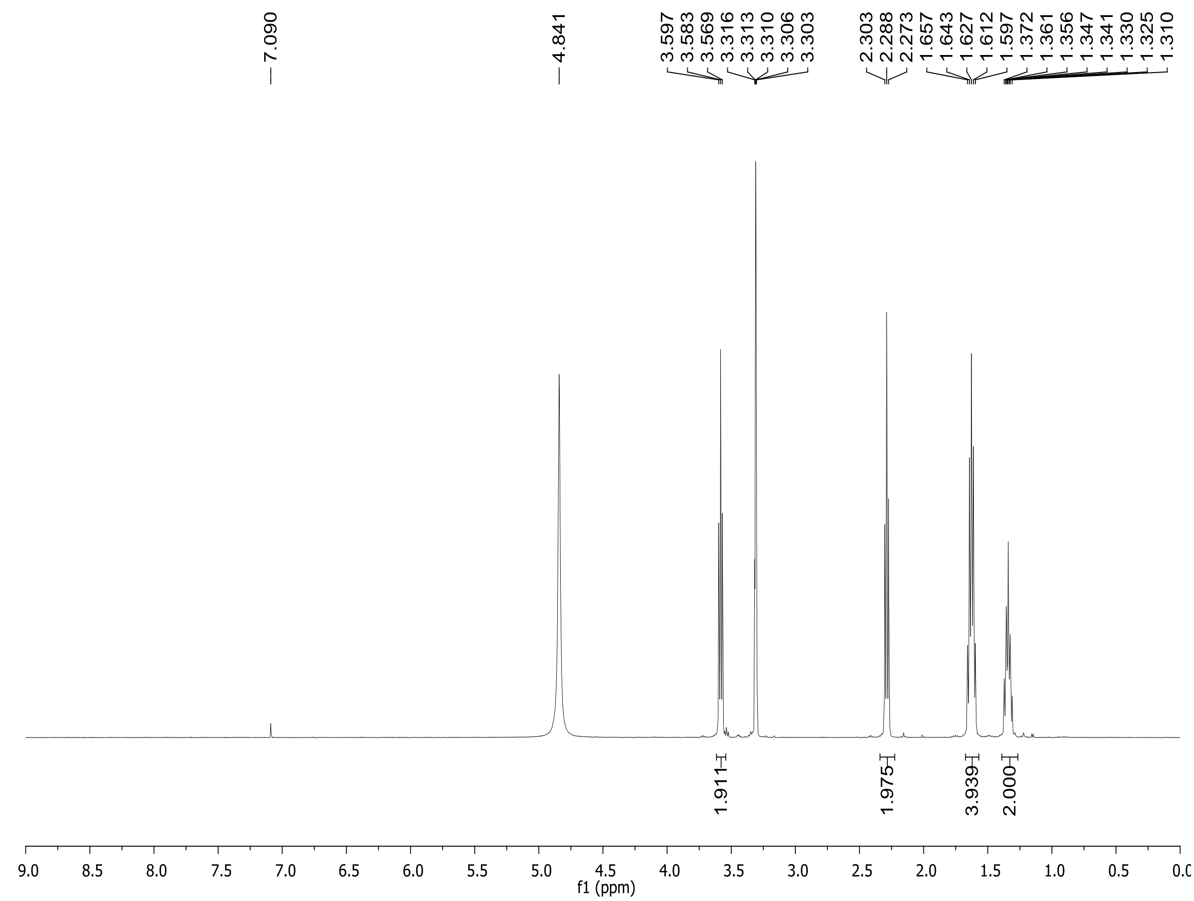
7.090
4.841
3.597
3.583
3.569
3.316
3.313
3.310
3.306
3.303
2.303
2.288
2.273
1.657
1.643
1.627
1.612
1.597
1.372
1.361
1.356
1.347
1.341
1.330
1.325
1.310
1.911
1.975
3.939
2.000
9.0 8.5 8.0 7.5 7.0 6.5 6.0 5.5 5.0 4.5 4.0 3.5 3.0 2.5 2.0 1.5 1.0 0.5
f1 (ppm)

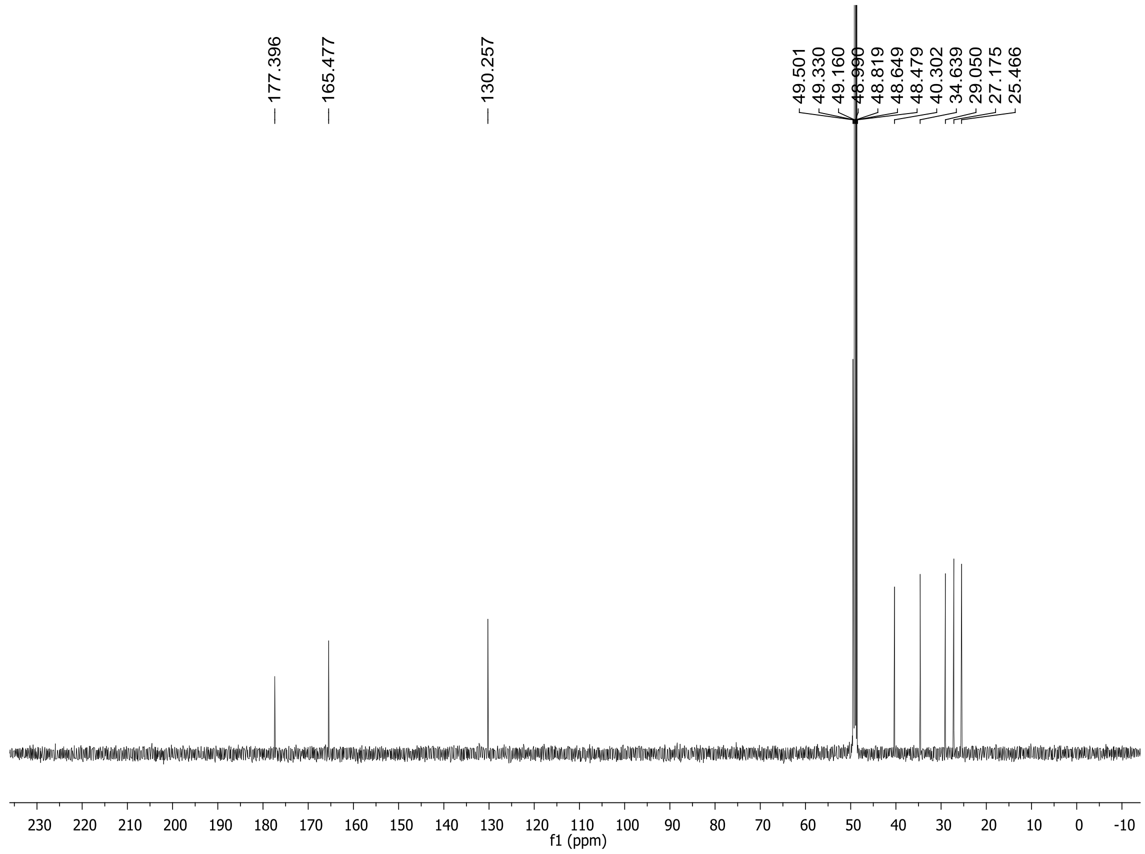
177.396
165.477
130.257
49.501
49.330
49.160
48.990
48.819
48.649
48.479
40.302
34.639
29.050
27.175
25.466
230 220 210 200 190 180 170 160 150 140 130 120 110 100 90 80 70 60 50 40 30 20 10 0 -10
f1 (ppm)

***N*-(*p*-Benzoic acid)-3,4-dibromo-maleimide 10**

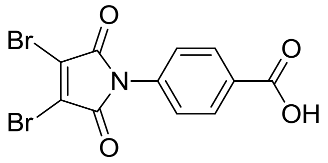

In a 25 mL round-bottom flask, 3,4-dibromomaleic anhydride (1.02 g, 4.0 mmol), prepared as reported,[2] and *p*-amino benzoic acid (0.549 g, 4 mmol, 1 eq.) were added. Next, AcOH (12 mL) was added and the mixture was heated at 120 °C with stirring for 40 minutes. The product crashes out from solution in the meantime. Then, the mixture was allowed to cool to 20 °C and filtered. The filter cake was washed with cold MeOH (2 mL) and DCM and dried under vacuum to afford **10** as an off-yellow solid (1.181 g, 3.15 mmol, 79%). Data for **10**: mp >260 °C. IR (pellet) ν_{max} 2828, 2544, 1778, 1728, 1689, 1591, 1376, 1286, 1100, 826, 723. ^{1}H NMR (600 MHz, DMSO-d$_6$) 7.51 (d, J = 8.4 Hz, 2H, Ar*H*), 8.06 (d, J = 8.4 Hz, 2H, Ar*H*), 13.2 (br, 1H, COO*H*); ^{13}C NMR (150 MHz, DMSO-d$_6$) 126.6 (ArCH), 129.8 (ArC), 130.1 (ArCH), 130.3 (ArC), 135.3 (C), 163.1 (CO) 166.7 (CO). LRMS (ESI) 373 (50, [M^{79}Br^{79}Br]$^+$), 375 (100, [M^{81}Br^{79}Br]$^+$), 377 (50, [M^{81}Br^{81}Br]$^+$); HRMS (ESI) calcd. for $C_{11}H_5NO_4Br_2$ [M^{79}Br^{79}Br]$^+$ 372.85798, observed: 372.85833.

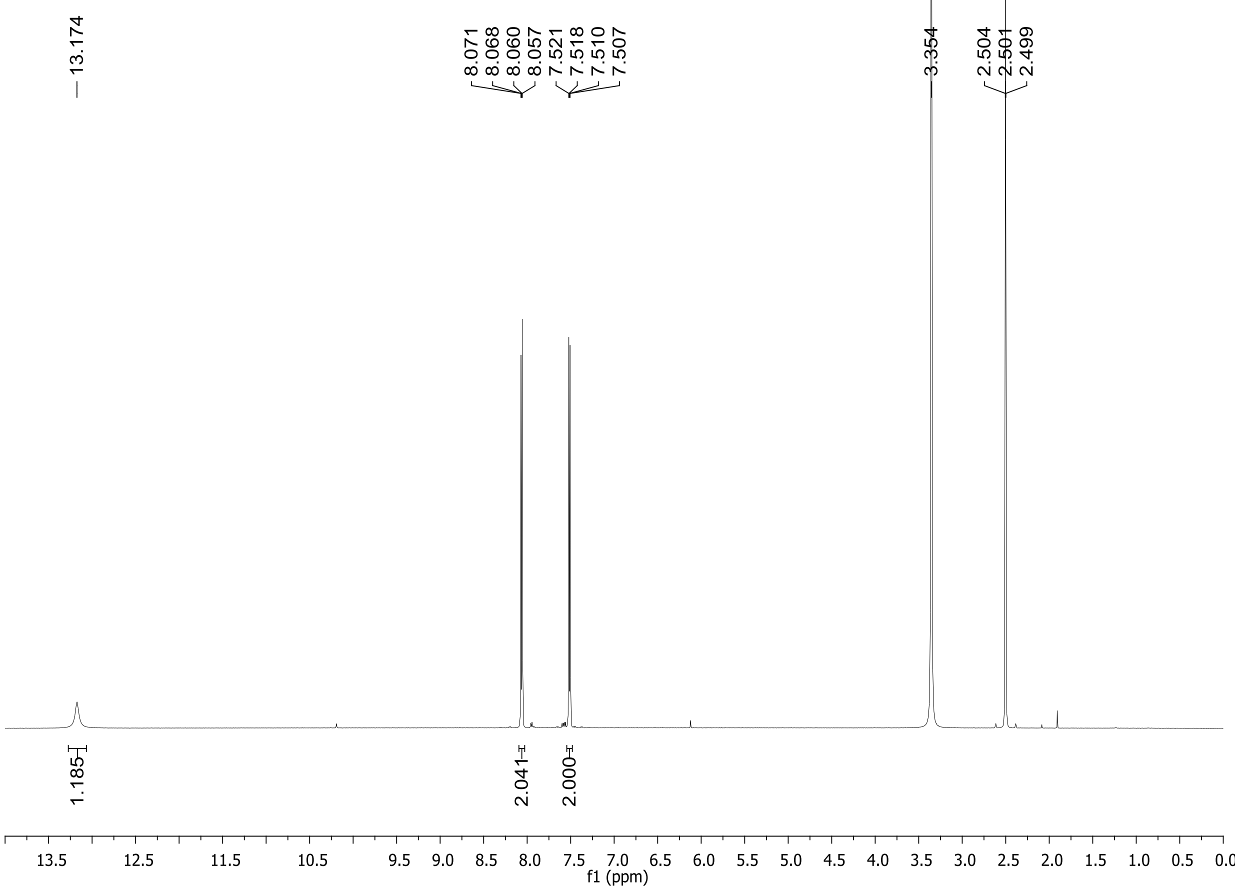
13.174
8.071
8.068
8.060
8.057
7.521
7.518
7.510
7.507
3.354
2.504
2.501
2.499
1.185
2.041
2.000
13.5 12.5 11.5 10.5 9.5 9.0 8.5 8.0 7.5 7.0 6.5 6.0 5.5 5.0 4.5 4.0 3.5 3.0 2.5 2.0 1.5 1.0 0.5 0.0
f1 (ppm)

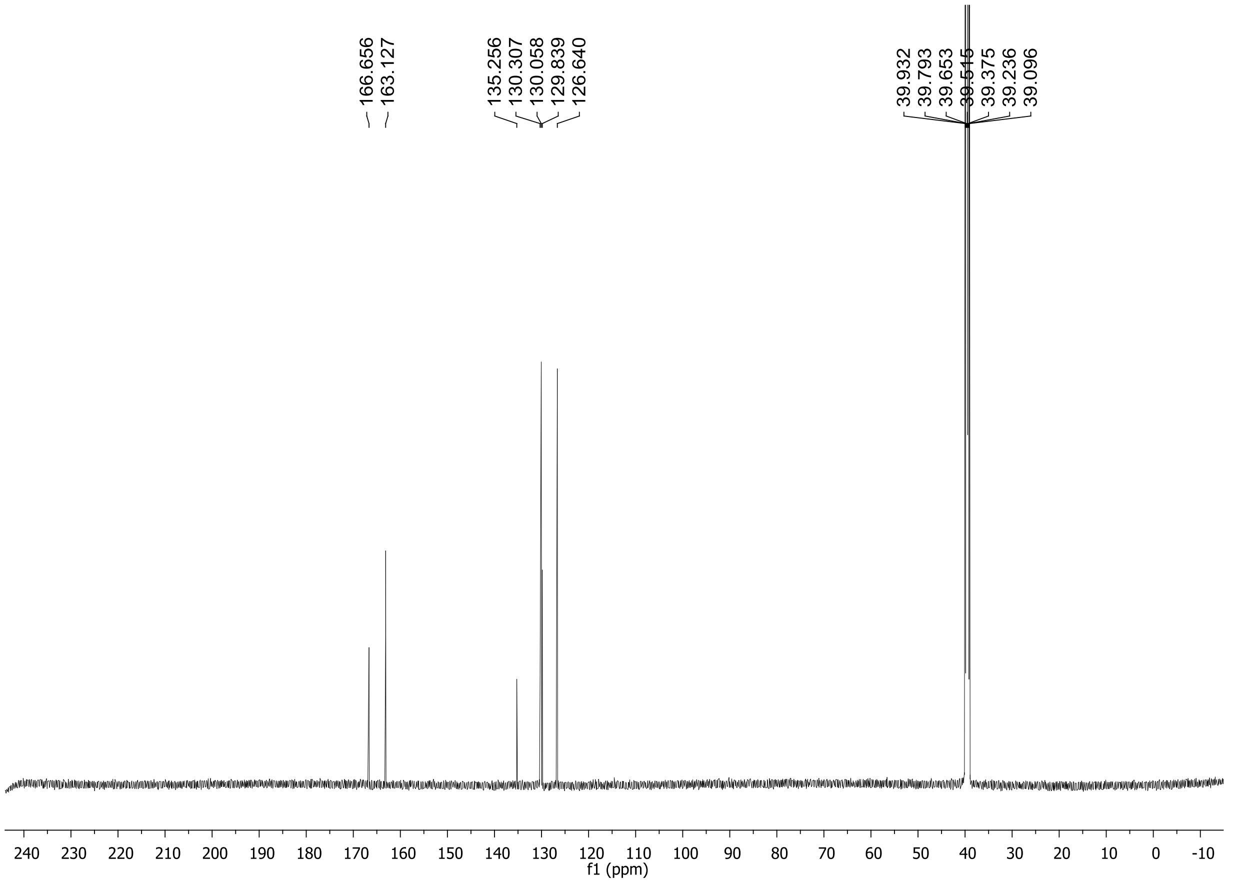
166.656
163.127
135.256
130.307
130.058
129.839
126.640
39.932
39.793
39.653
39.515
39.375
39.236
39.096
240 230 220 210 200 190 180 170 160 150 140 130 120 110 100 90 80 70 60 50 40 30 20 10 0 -10
f1 (ppm)

Maleimide-*N*-hexanoic acid 11[3, 4]

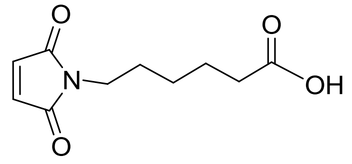

In a 10 mL round-bottom flask, maleic anhydride (196 mg, 2.0 mmol) and 6-aminocaproic acid (262 mg, 2 mmol, 1 eq.) were suspended in AcOH (4 mL). Next, the mixture was heated to 120 °C, dissolving all reagents. The mixture was stirred at 120 °C for 3 h. Afterwards, cooled down to 20 °C and concentrated under vacuum, redissolved in toluene and concentrated once more to dryness, affording the crude as a yellow oil. The crude oil was purified by flash chromatography on silica with DCM:MeOH (10:1 v/v with 1% AcOH) to afford **11** as a white solid (184 mg, 0.87 mmol, 44%) Data for **11**: mp = 83-85 °C, reported 89-90 °C,[3] 79-80 °C.[4] IR (pellet) ν_{max} 3086, 2938, 2872, 1686, 1409, 1310, 1259, 1208, 1109, 910, 834, 695. ^{1}H NMR (500 MHz, $CDCl_3$) 1.30 (m, J = 7.5 Hz, 2H, CH_2), 1.54-1.65 (overlapped quint., J = 7.5 Hz, 4H, CH_2), 2.30 (t., J = 7.5 Hz, 2H, CH_2), 3.49 (t, J = 7.5 Hz, 2H, CH_2), 6.67 (s, 2H, CH); ^{13}C NMR (125 MHz, $CDCl_3$) 24.1 (CH_2), 26.2 (CH_2), 28.2 (CH_2), 33.8 (CH_2), 37.6 (CH_2), 134.1 (2×CH), 170.9 (2×CO), 179.6 (CO). LRMS (ESI) 234 (100, $[M+Na]^+$), 212 (65, $[M+H]^+$); HRMS (ESI) calcd. for $C_{10}H_{14}NO_4$ $[M+H]^+$ 212.0917, observed: 212.0918.

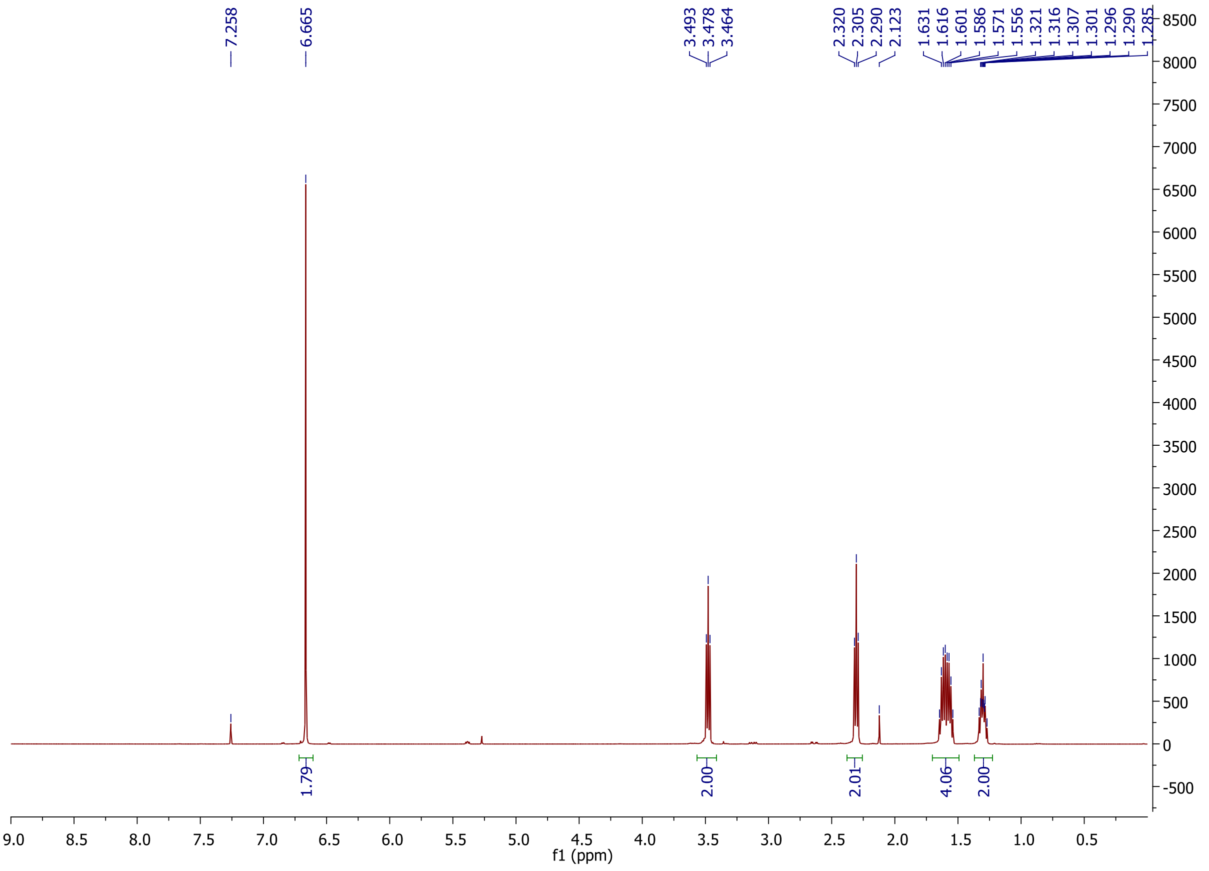
7.258
6.665
3.493
3.478
3.464
2.320
2.305
2.290
2.123
1.631
1.616
1.601
1.586
1.571
1.556
1.321
1.316
1.307
1.301
1.296
1.290
1.285
1.79
2.00
2.01
4.06
2.00
f1 (ppm)

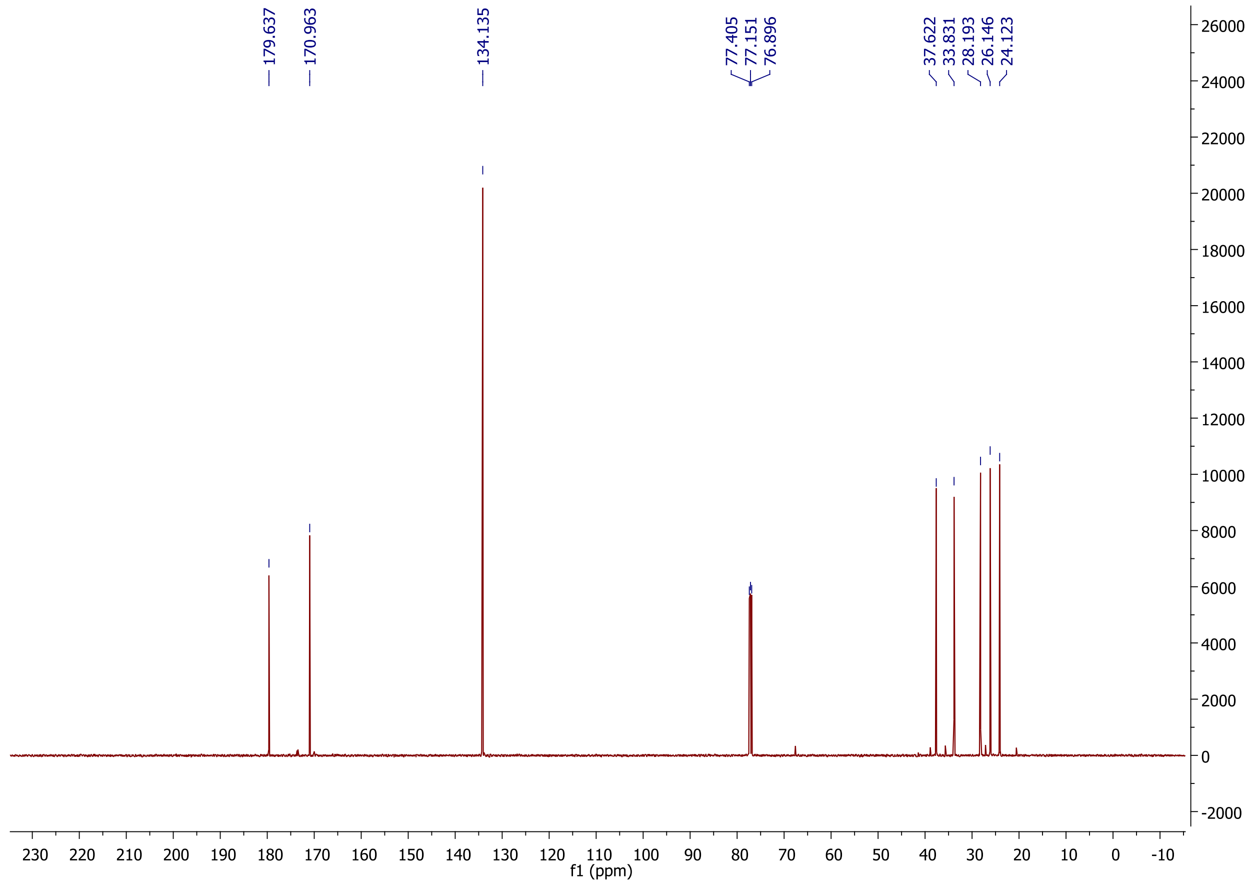
179.637
170.963
134.135
77.405
77.151
76.896
37.622
33.831
28.193
26.146
24.123
f1 (ppm)

3,4-Dithiophenoyl-maleimide-*N*-hexanoic acid 12

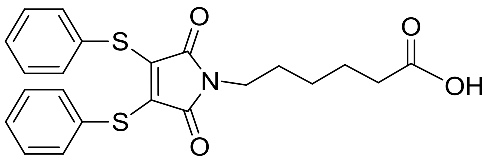

In a 25 mL round-bottom flask under argon, compound **9** (369 mg, 1.0 mmol) was dissolved in MeOH (4 mL). Then, added NaOAc (172 mg, 2.1 mmol, 2.1 eq.). Next, a solution of thiophenol (225 µL, 2.2 mmol, 2.2 eq.) in MeOH (2 mL) under argon was added to the reaction mixture dropwise over 5 minutes, giving an orange solution. The mixture was stirred at 20 °C for 20 minutes. Then, quenched with 20 mM HCl (10 mL, 0.2 mmol, 0.2 eq.) and extracted with EtOAc (2×20 mL). The combined organic layer was dried ($MgSO_4$), filtered and concentrated under vacuum to yield a yellow solid which was purified by flash chromatography on silica with petroleum ether:EtOAc (2:5 v/v) to afford **12** as a yellow solid (371 mg, 0.87 mmol, 87%). Data for **12**: mp = 117-118 °C. IR (pellet) ν_{max} 3058, 2940, 2870, 1766, 1697, 1541, 1395, 1176, 1049, 915, 842, 747, 687. ^{1}H NMR (600 MHz, MeOD-d_4) 1.31 (quint., J = 7.2 Hz, 2H, CH_2), 1.57-1.63 (overlapped quint., J = 7.2 Hz, 4H, CH_2), 2.27 (t, J = 7.2 Hz, 2H, CH_2), 3.51 (t, J = 7.2 Hz, 2H, CH_2), 7.17-7.18 (overlapped m, 4H, Ar*H*), 7.24-7.29 (overlapped m, 6H, Ar*H*); ^{13}C NMR (150 MHz, MeOD-d_4) 25.5 (CH_2), 27.3 (CH_2), 29.1 (CH_2), 34.8 (CH_2), 39.5 (CH_2), 129.2 (ArCH), 130.1 (ArCH), 130.7 (ArC), 132.4 (ArCH), 137.0 (C), 168.4 (CO), 177.5 (CO). LRMS (ESI) 427 (100, $[M]^+$); HRMS (ESI) calcd. for $C_{22}H_{21}NO_4S_2$ $[M]^+$ 427.09065, observed: 427.09131.

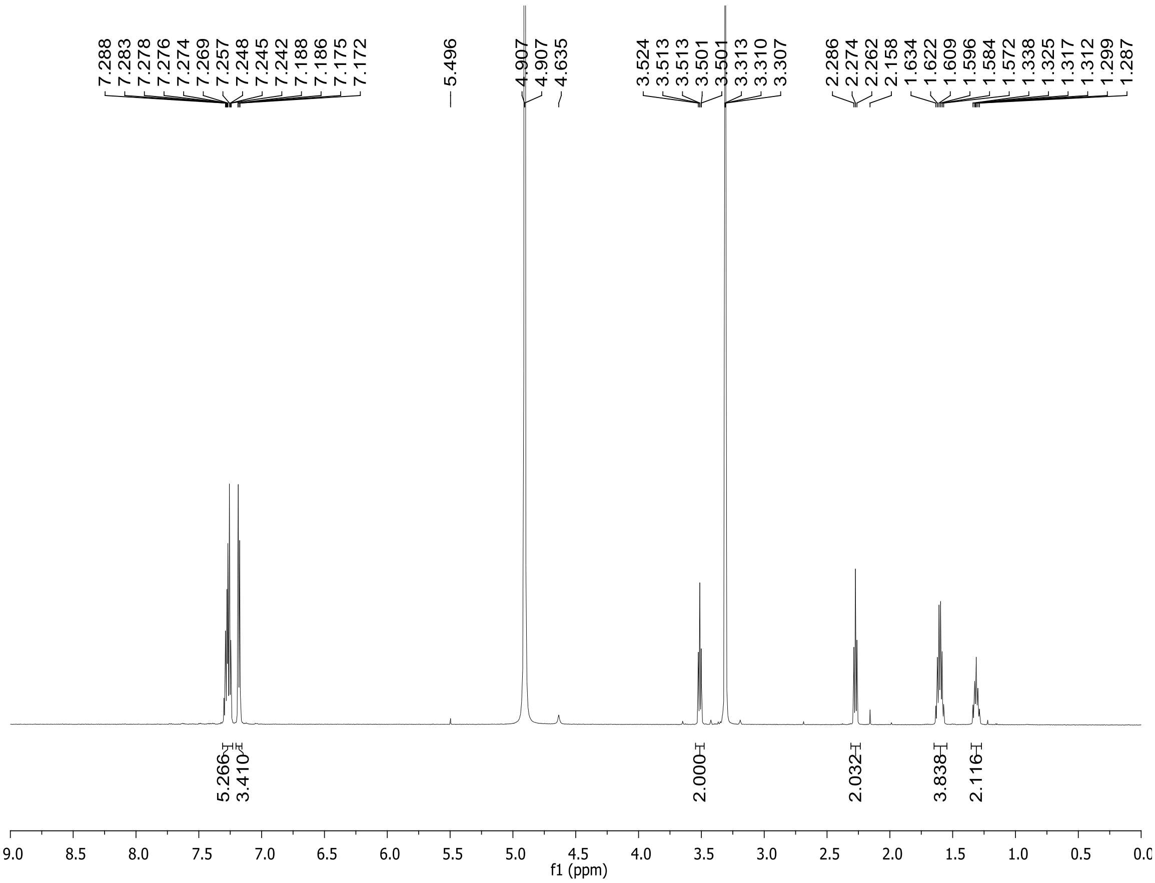
7.288
7.283
7.278
7.276
7.274
7.269
7.257
7.248
7.245
7.242
7.188
7.186
7.175
7.172
5.496
4.907
4.907
4.635
3.524
3.513
3.513
3.501
3.501
3.313
3.310
3.307
2.286
2.274
2.262
2.158
1.634
1.622
1.609
1.596
1.584
1.572
1.338
1.325
1.317
1.312
1.299
1.287
5.266
3.410
2.000
2.032
3.838
2.116
f1 (ppm)

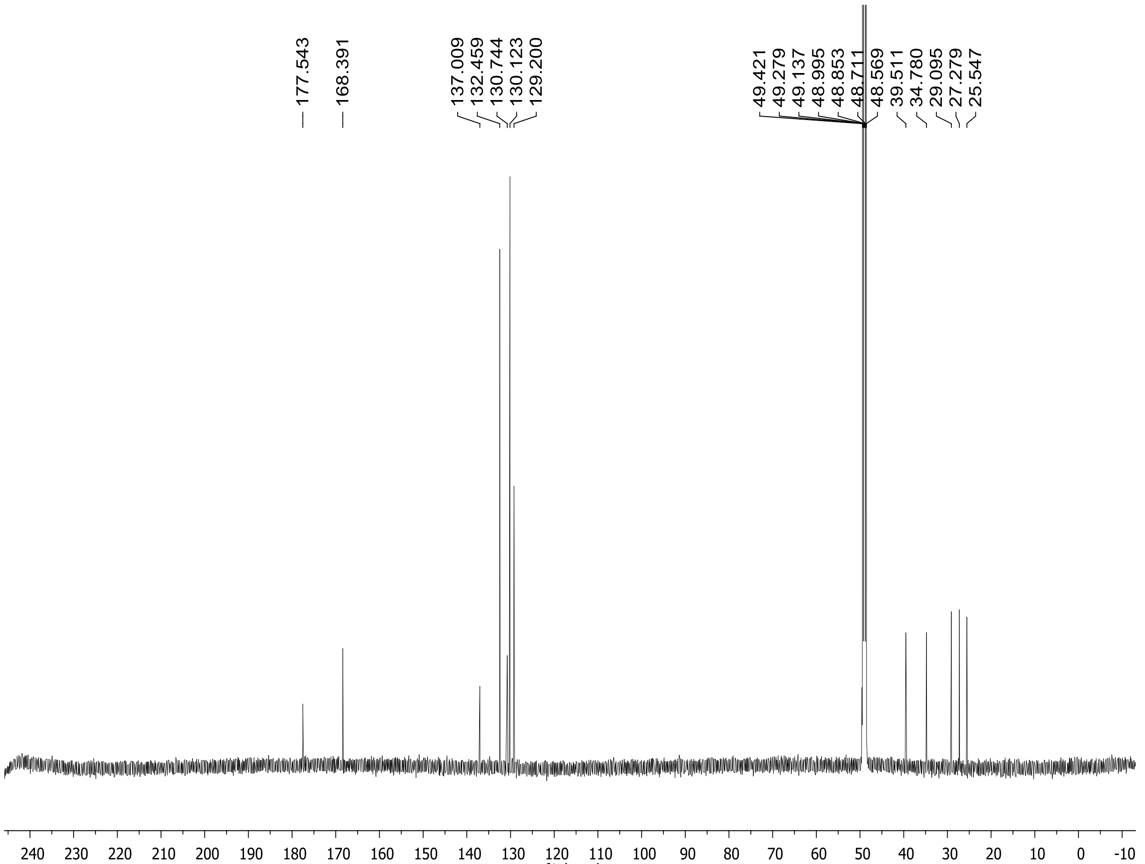
177.543
168.391
137.009
132.459
130.744
130.123
129.200
49.421
49.279
49.137
48.995
48.853
48.711
48.569
39.511
34.780
29.095
27.279
25.547
f1 (ppm)

***N*-(*p*-Benzoic acid)-3,4-dithiophenoyl-maleimide 13**

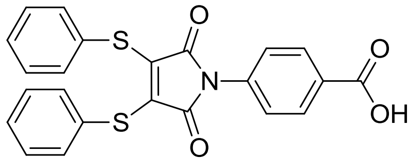

In a 25 mL round-bottom flask, compound **10** (375 mg, 1.0 mmol) was dissolved in THF (12 mL). Then, added NaOAc (172 mg, 2.1 mmol, 2.1 eq.). Next, a solution of thiophenol (225 µL, 2.2 mmol, 2.2 eq.) in THF (2 mL) under argon was added to the reaction mixture dropwise over 5 minute. The mixture was stirred at 20 °C for 90 minutes, slowly turning yellow overtime. Then, concentrated under vacuum, redissolved in DCM (80 mL) and sonicated for 3 minutes. Then, filtered to remove solids and concentrated the filtrate to give a yellow solid which was purified by flash chromatography on silica with DCM:MeOH (2:5 v/v) to afford **13** as a yellow solid (189 mg, 0.44 mmol, 44%). Data for **13**: mp = 236-237 °C. IR (pellet) ν_{max} 3120, 2163, 1708, 1431, 1053, 967, 733. ^{1}H NMR (500 MHz, DMSO-d$_6$) 7.30 (overlapped m, 10H, Ar*H*), 7.51 (d, *J* = 8.4 Hz, 2H, Ar*H*), 8.04 (d, *J* = 8.4 Hz, 2H, Ar*H*); ^{13}C NMR (125 MHz, DMSO-d$_6$) 126.1 (ArCH), 128.0 (ArC), 128.9 (ArCH), 129.0 (ArCH), 129.9 (ArCH), 130.7 (overlapped, ArCH and ArC), 135.8 (C), 165.2 (CO) 166.7 (CO). LRMS (ESI) 432 (100, [M-H]$^+$); HRMS (ESI) calcd. for $C_{23}H_{14}NO_4S_2$ [M-H]$^+$ 432.0364, observed: 432.0360.

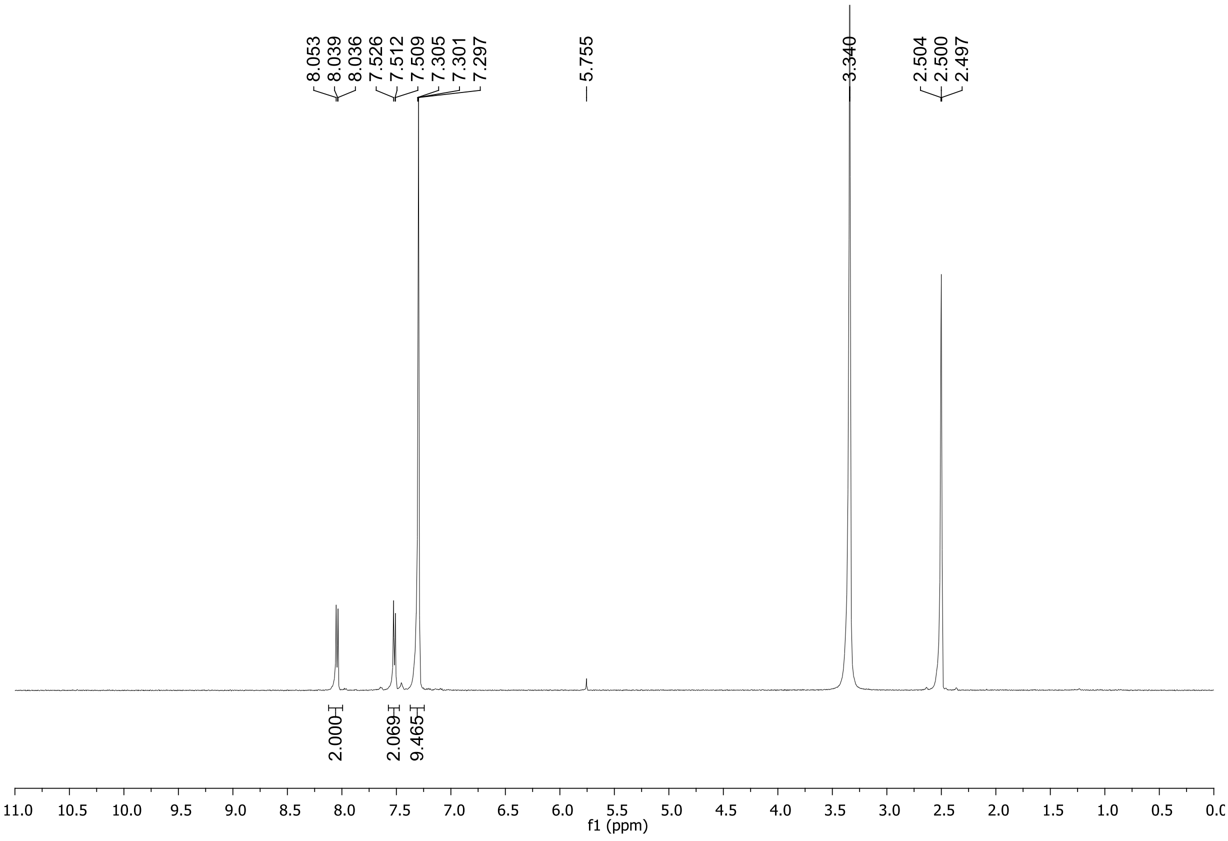
8.053
8.039
8.036
7.526
7.512
7.509
7.305
7.301
7.297
5.755
3.340
2.504
2.500
2.497
2.000
2.069
9.465
11.0 10.5 10.0 9.5 9.0 8.5 8.0 7.5 7.0 6.5 6.0 5.5 5.0 4.5 4.0 3.5 3.0 2.5 2.0 1.5 1.0 0.5 0.0
f1 (ppm)

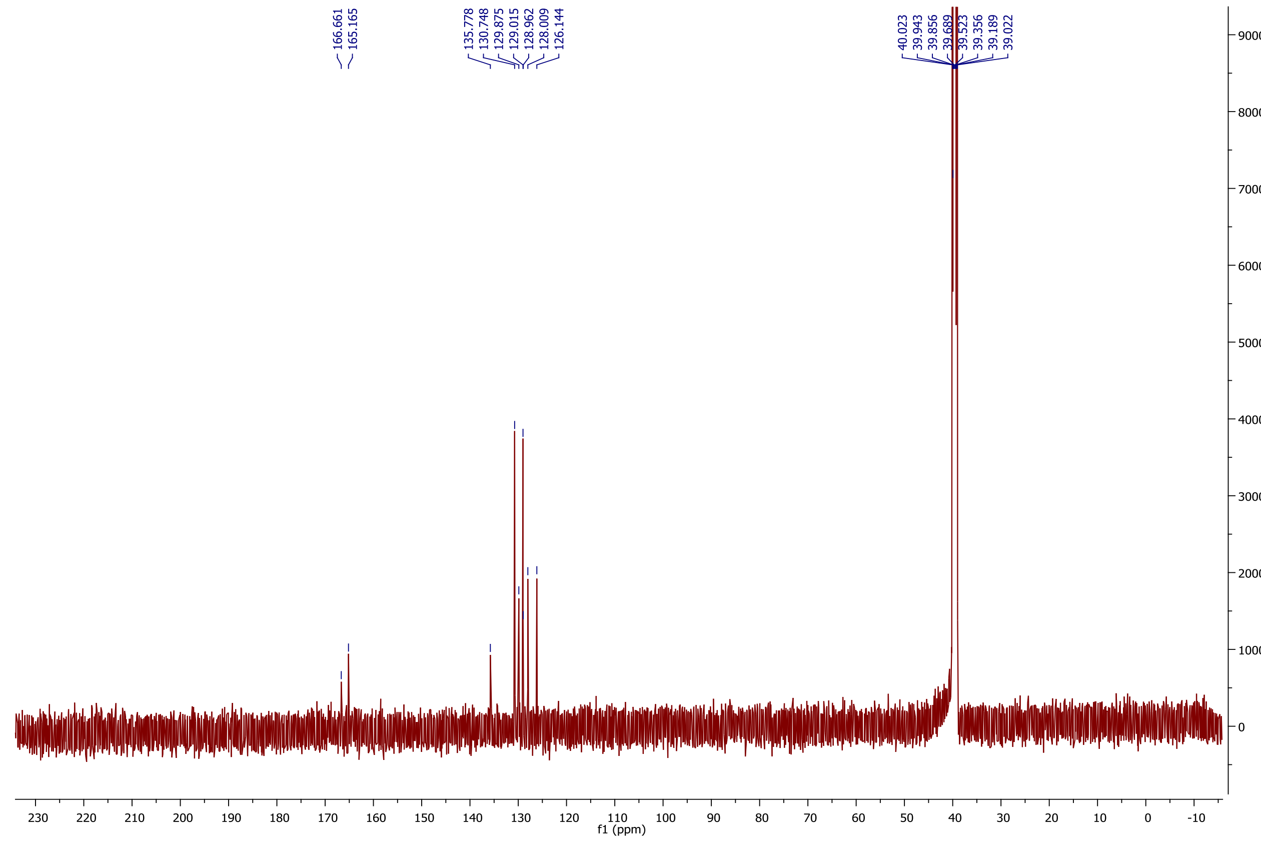
166.661
165.165
135.778
130.748
129.875
129.015
128.962
128.009
126.144
40.023
39.943
39.856
39.689
39.523
39.356
39.189
39.022
230 220 210 200 190 180 170 160 150 140 130 120 110 100 90 80 70 60 50 40 30 20 10 0 -10
f1 (ppm)
9000 8000 7000 6000 5000 4000 3000 2000 1000 0

N-(Hexanamide-N-doxorubicin)-3,4-dithiophenoyl-maleimide 3

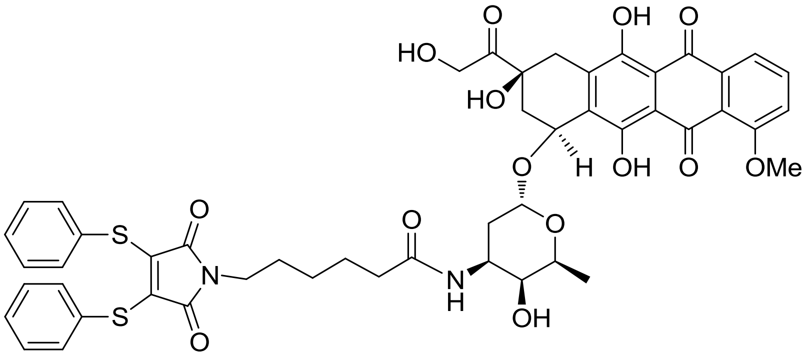

In a 10 mL round-bottom flask under argon, compound **12** (7.63 mg, 17.8 μmol, 1.0 eq.), HOBt (0.25 mg, 1.78 μmol, 0.10 eq.) and HBTU (6.7 mg, 17.8 μmol, 1.0 eq.) were dissolved in DMF (0.5 mL) to give a yellow solution. Next, a 0.378 M solution of DIPEA in DMF (50 μL, 18.9 μmol, 1.1 eq.) was added and the mixture was stirred for 3 min. Then, a solution of doxorubicin hydrochloride (10 mg, 17.2 μmol, 1.0 eq.) with DIPEA (3.27 μL, 18.9 μmol, 1.1 eq.) in DMF (0.7 mL) was added. The solution turned red upon addition. The solution was stirred at 20 °C for 6 h. Then, concentrated under vacuum, added DCM (20 mL) and washed with aqueous saturated LiCl solution (3×10 mL), 15% K_2CO_3 (10 mL), 15% citric acid solution (10 mL) and water (10 mL). The organic layer was dried ($MgSO_4$), filtered and concentrated under vacuum to yield a red solid which was purified by flash chromatography on silica with DCM:EtOAc:MeOH (10:10:1 v/v) to afford **3** as a red solid (15.1 mg, 16.0 μmol, 92%). Data for **3**: no melting point, decomposes at 132-135 °C. IR (pellet) ν_{max} 3469, 2435, 1702, 1617, 1580, 1398, 1207, 1077, 980, 735, 690. ^{1}H NMR (600 MHz, MeOD-d_4 + drops of $CDCl_3$) 1.20 (quint., *J* = 7.2 Hz, 2H, C*H*$_2$), 1.27 (d, *J* = 6.6 Hz, 3H, C*H*$_3$ DOX), 1.47-1.59 (overlapped quint., *J* = 7.2 Hz, 4H, C*H*$_2$), 1.74 (dd, *J* = 13.2, 4.8 Hz, 1H, C*H*$_2$ DOX), 1.99 (dt, *J* = 13.2, 3.6 Hz, 1H, C*H*$_2$ DOX), 2.11 (m, *J* = 4.8 Hz, 1H, C*H*$_2$ DOX), 2.15 (t, *J* = 7.2 Hz, 2H, C*H*$_2$), 2.33 (d, *J* = 14.4, 1H, C*H*$_2$ DOX), 2.85 (d, *J* = 18.6, 1H, C*H*$_2$ DOX), 3.01 (d, *J* = 18.6, 1H, C*H*$_2$ DOX), 3.38 (t, *J* = 7.2, 2H, C*H*$_2$), 3.61 (s, 1H, C*H* DOX), 3.95 (s, 3H, C*H*$_3$ DOX), 4.14 (dq, *J* = 13.2, 2.4, 1H, C*H* DOX), 4.25 (q, *J* = 6.6, 1H, C*H* DOX), 4.74

(ABq, *J* = 19.8, ν_{AB} = 17.5, 2H, CH_2 DOX), 5.07 (dt, *J* = 2.4, 1.8, 1H, C*H* DOX), 5.41 (d, *J* = 3.6, 1H, C*H* DOX), 7.06-7.07 (overlapped m, 4H, Ar*H*), 7.16-7.23 (overlapped m, 6H, Ar*H*), 7.43 (d, *J* = 8.4, 1H, Ar*H*, DOX), 7.72 (t, *J* = 8.4, 1H, Ar*H* DOX), 7.78 (d, *J* = 7.8, 1H, Ar*H* DOX); ^{13}C NMR (150 MHz, MeOD-d_4 + drops of $CDCl_3$) 17.4 (CH_3), 26.4 (CH_2), 27.2 (CH_2), 29.1 (CH_2), 30.5 (CH_2), 34.1 (CH_2), 36.7 (CH_2), 37.3 (CH_2), 39.5 (CH_2), 47.0 (CH), 57.1 (CH_3), 65.8 (CH_2), 68.6 (CH), 69.9 (CH), 71.2 (CH), 77.4 (C), 102.2 (CH), 112.2 (ArC), 112.4 (ArC), 120.2 (ArCH), 120.5 (ArCH), 121.5 (ArC), 129.2 (ArCH), 130.1 (ArCH), 130.6 (ArC), 132.5 (ArCH), 135.1 (ArC), 135.7 (ArC), 136.3 (ArC), 136.9 (C), 137.1 (ArCH), 156.2 (ArC), 157.3 (ArC), 162.3 (ArC), 168.2 (CO), 175.4 (CO), 187.6 (CO), 188.0 (CO), 214.7 (CO). LRMS (ESI) 975 (30, $[M+Na]^+$); HRMS (ESI) calcd. for $C_{49}H_{48}N_2O_{14}S_2Na$ $[M+Na]^+$ 975.2445, observed: 975.2427.

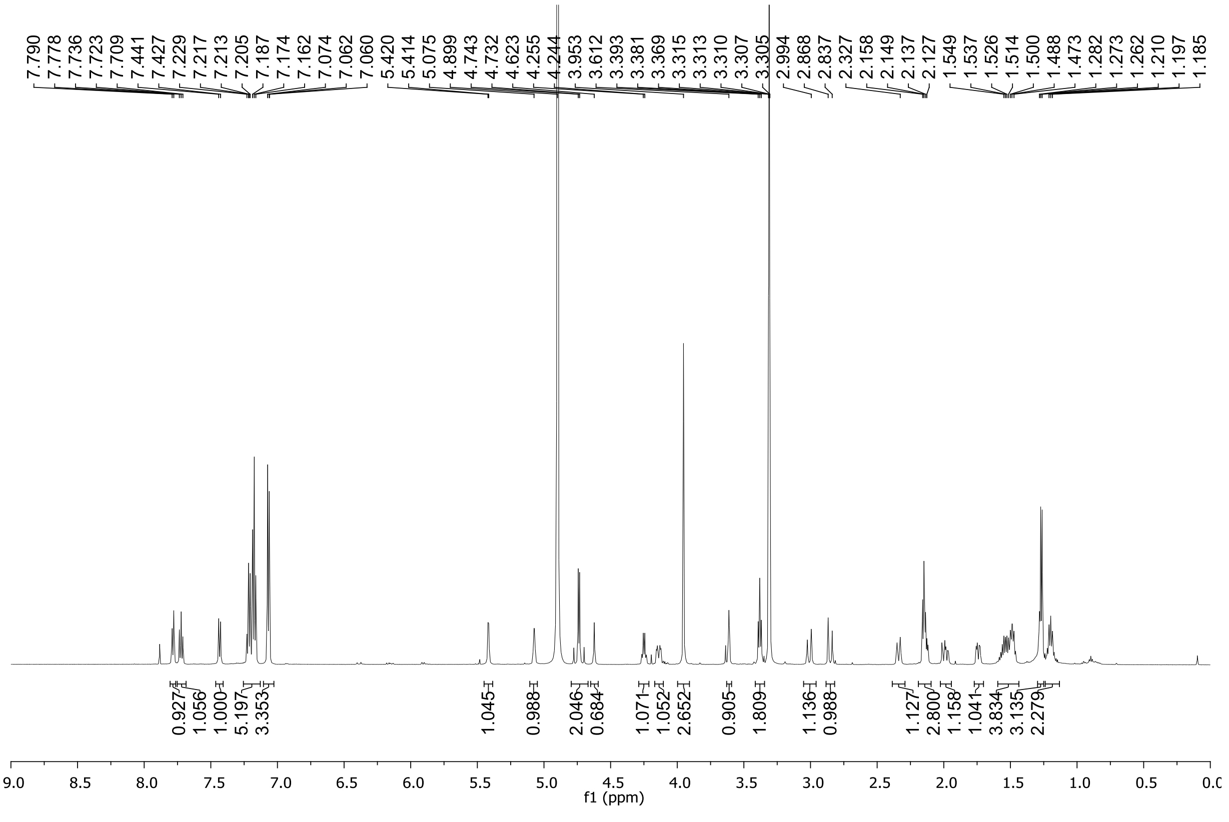

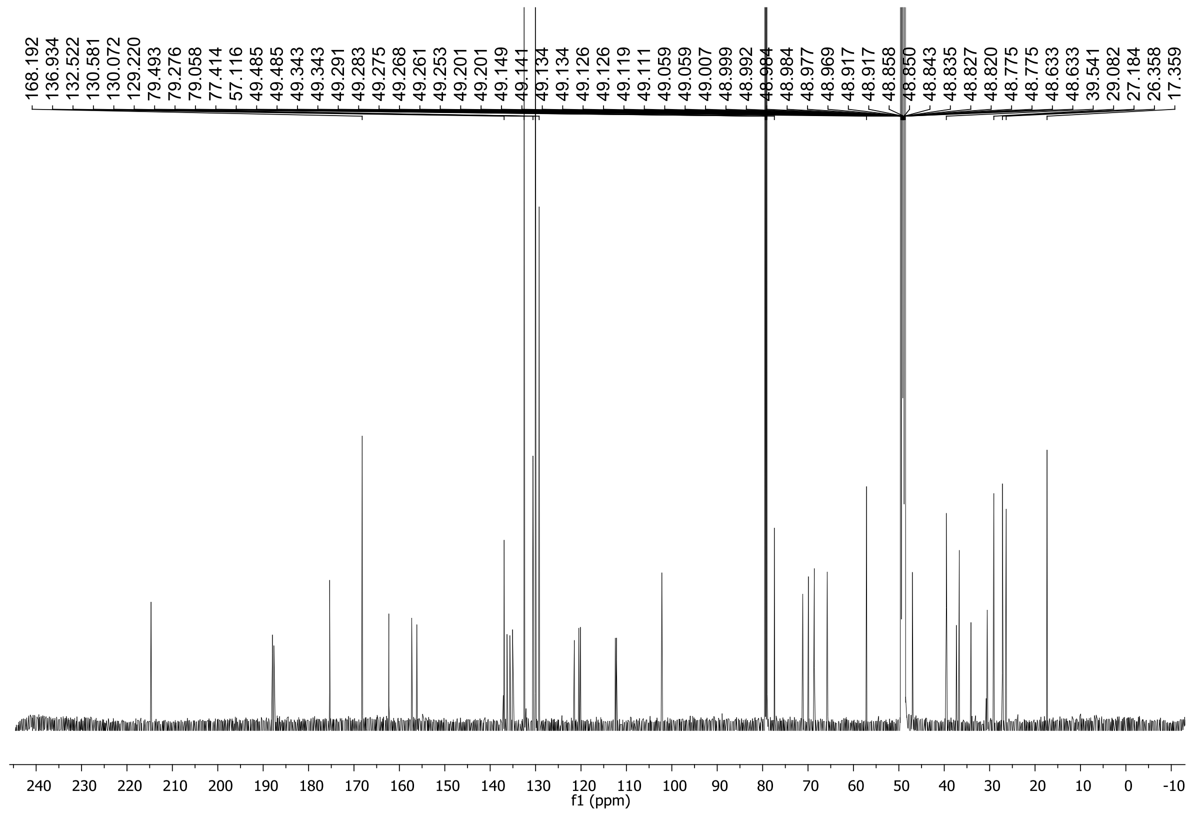

***N*-(*p*-Benzamide-*N*-doxorubicin)-3,4-dithiophenoyl-maleimide 4**

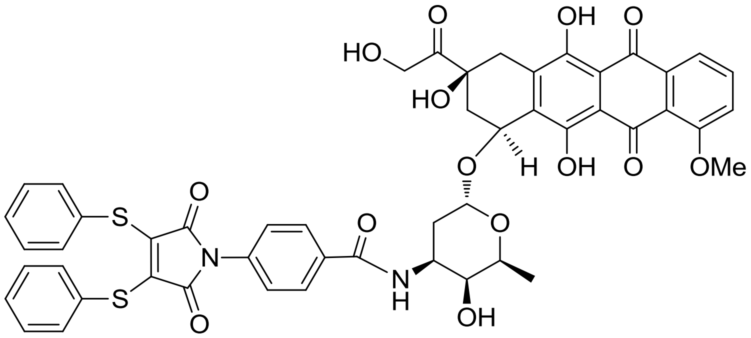

In a 10 mL round-bottom flask under argon, compound **13** (7.46 mg, 17.2 μmol, 1.0 eq.), HOBt (0.25 mg, 17.8 μmol, 0.10 eq.) and HBTU (6.7 mg, 17.8 μmol, 1.0 eq.) were dissolved in DMF (0.5 mL) to give a yellow solution. Next, a 0.378 M solution of DIPEA in DMF (50 μL, 18.9 μmol, 1.1 eq.) was added and the mixture was stirred for 3 min. Then, a solution of doxorubicin hydrochloride (10 mg, 17.2 μmol, 1.0 eq.) with DIPEA (3.27 μL, 18.9 μmol, 1.1 eq.) in DMF (0.7 mL) was added. The solution turned red upon addition. The solution was stirred at 20 °C for 6 h. Then, added DCM (10 mL) and washed with 0.68 M acetate buffer pH 5 (10 mL) and aqueous saturated LiCl solution (3×10 mL). The organic layer was dried ($MgSO_4$), filtered and concentrated under vacuum to yield a red solid which was purified by flash chromatography on silica with DCM:EtOAc:MeOH (20:20:1 v/v) to afford **4** as a red solid (14.9 mg, 15.5 μmol, 90%). Data for **4**: IR (pellet) ν_{max} 3516, 3407, 2926, 1714, 1615, 1578, 1374, 1284, 1207, 984, 732. ^{1}H NMR (600 MHz, DMSO-d$_6$) 1.16 (d, *J* = 6.6 Hz, 3H, C*H*$_3$), 1.54 (dd, *J* = 13.2, 4.2 Hz, 1H, C*H*$_2$ DOX), 2.08 (dt, *J* = 13.2, 3.6 Hz, 1H C*H*$_2$ DOX), 2.12-2.25 (ABq, *J* = 12.6, ν_{AB} = 61, 2H, C*H*$_2$ DOX), 3.00 (q, *J* = 18.6, 2H, C*H*$_2$ DOX), 3.56 (br, 1H, C*H* DOX), 3.97 (s, 3H, C*H*$_3$ DOX), 4.20 (m, 1H, C*H* DOX), 4.25 (q, *J* = 6.6, 1H, C*H* DOX), 4.59 (d, *J* = 5.4 Hz, 2H, C*H*$_2$ DOX), 4.88 (d, *J* = 5.4 Hz, 1H, O*H* DOX), 4.90 (t, *J* = 6.0 Hz, 1H, O*H* DOX), 4.97 (t, *J* = 4.2 Hz, 1H, C*H* DOX), 5.28 (d, *J* = 2.4 Hz, 1H, C*H* DOX), 5.52 (s, 1H, O*H*), 7.21-7.35 (overlapped m, 10H, Ar*H*), 7.43 (d, *J* = 8.4, 2H, Ar*H* overlapped with N*H*), 7.65 (t, *J* = 4.8, 1H, Ar*H*), 7.90-7.91 (overlapped d, *J* = 7.2 Hz, 4H,

Ar*H*), 7.78 (d, *J* = 7.8, 1H, Ar*H*), 13.29 (s, 1H, O*H*), 14.05 (s, 1H, O*H*); ^{13}C NMR (150 MHz, DMSO-d$_6$) 17.1 (CH_3), 29.5 (CH_2), 32.1 (CH_2), 36.8 (CH_2), 46.2 (C*H*), 56.6 (CH_3), 63.7 (CH_2), 66.7 (CH), 67.9 (CH), 70.1 (CH), 75.0 (C), 100.5 (CH), 110.7 (ArC), 110.9 (ArC), 119.0 (ArCH), 119.8 (ArCH), 120.1 (ArC), 126.1 (ArCH), 127.9 (ArCH) 128.0 (ArCH), 128.9 (ArC), 129.0 (ArCH overlapped with ArC), 133.9 (ArC), 130.6 (ArH), 134.2 (ArC), 134.7 (ArC), 135.6 (C overlapped with ArC) , 136.3 (ArCH), 154.5 (ArC), 156.2 (ArC), 160.8 (ArC), 165.2 (CO), 165.4 (CO), 186.5 (CO), 186.6 (CO), 213.9 (CO). LRMS (ESI) 981 (24, [M+Na]$^+$); HRMS (ESI) calcd. for $C_{50}H_{42}N_2O_{14}S_2Na$ [M+Na]$^+$ 981.1975, observed: 981.1976.

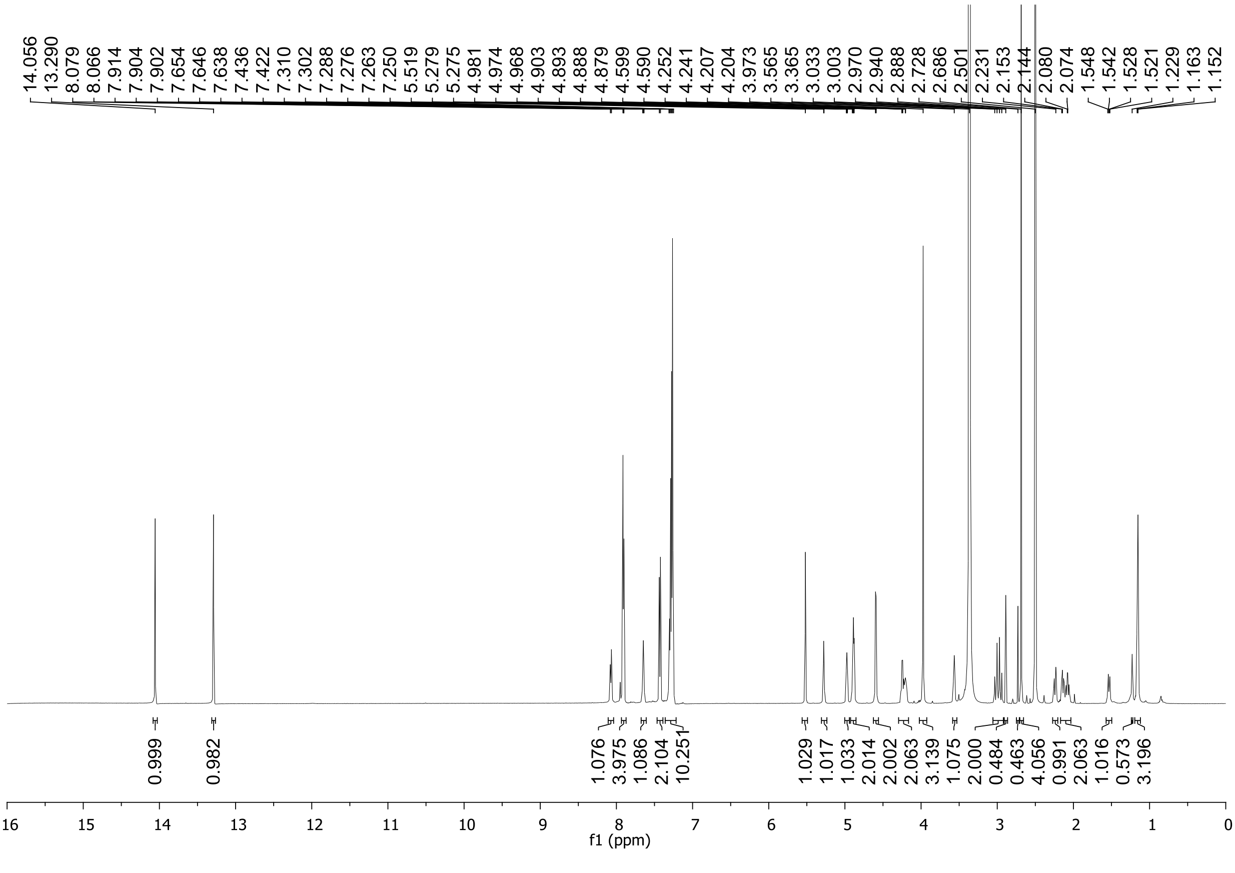

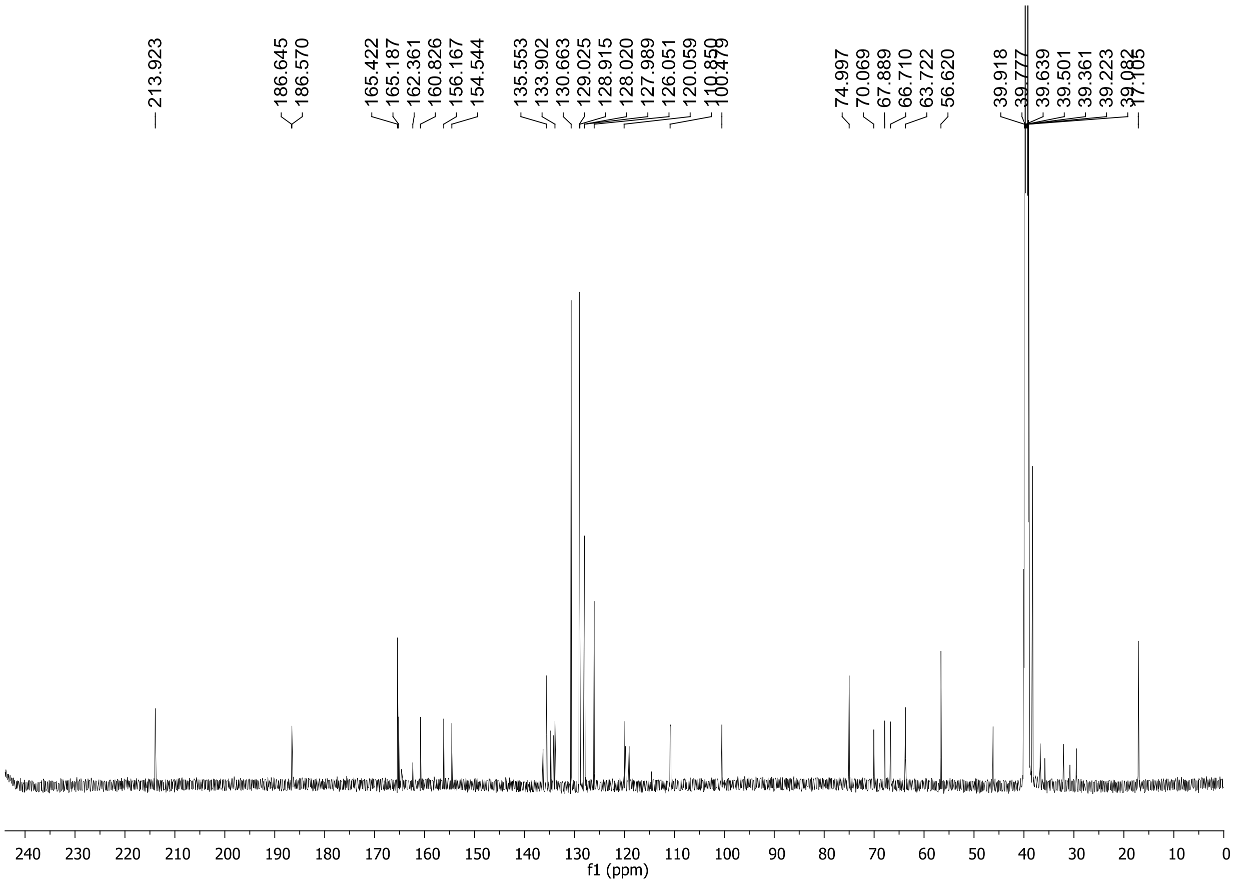

N-(*N*-Doxorubicinhexanamide)-maleimide 6

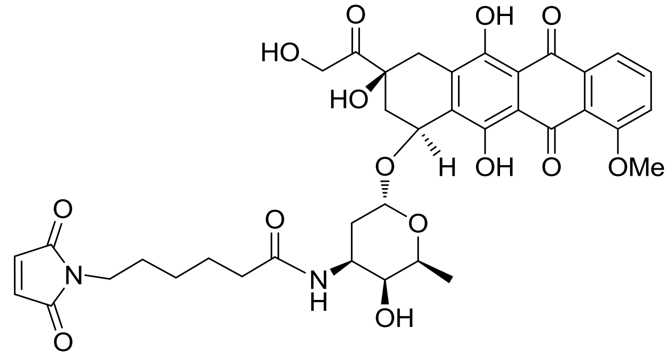

In a 10 mL round-bottom flask under argon, compound **11** (3.63 mg, 17.2 µmol, 1.0 eq.), HOBt (0.25 mg, 17.8 µmol, 0.10 eq.) and HBTU (6.7 mg, 17.8 µmol, 1.0 eq.) were dissolved in DMF (0.5 mL). Next, a 0.378 M solution of DIPEA in DMF (50 µL, 18.9 µmol, 1.1 eq.) was added and the mixture was stirred for 5 min. Then, a solution of doxorubicin hydrochloride (10 mg, 17.2 µmol, 1.0 eq.) with DIPEA (3.27 µL, 18.9 µmol, 1.1 eq.) in DMF (0.7 mL) was added. The solution turned red upon addition. The solution was stirred at 20 °C

for 5 h. Then, quenched with a 0.68 M acetate buffer pH 5 (10mL) and added DCM (10 mL). Separated layers and further washed organic layer with aqueous saturated LiCl solution (6×20 mL). The organic layer was dried ($MgSO_4$), filtered and concentrated under vacuum to yield a red solid which was purified by flash chromatography on silica with DCM:EtOAc:MeOH (20:20:3 v/v) to afford **6** as a red solid (10.2 mg, 0.0139 mmol, 80%). Data for **6**: no melting point, decomposes at 179-185 °C. IR (pellet) ν_{max} 3459, 2935, 2504, 1700, 1615, 1409, 1273, 979, 695. ^{1}H NMR (600 MHz, MeOD-d_4) 1.21 (quint., J = 7.2 Hz, 2H, CH_2), 1.27 (d, J = 6.6 Hz, 3H, CH_3 DOX), 1.45-1.57 (overlapped quint., J = 7.2 Hz, 4H, CH_2), 1.72 (dd, J = 13.2, 4.8 Hz, 1H, CH_2 DOX), 1.98 (dt, J = 13.2, 4.2 Hz, 1H CH_2 DOX), 2.15 (dd, J = 14.4, 4.8 Hz, 1H, CH_2 DOX), 2.33 (t, J = 7.2 Hz, 2H, CH_2), 2.30 (d, J = 14.4, 1H, CH_2 DOX), 2.78 (d, J = 18.0, 1H, CH_2 DOX), 2.96 (d, J = 17.4, 1H, CH_2 DOX), 3.37 (dt, J = 7.2, 2.4, 2H, CH_2), 3.60 (br, 1H, CH DOX), 3.94 (s, 3H, CH_3 DOX), 4.12 (dq, J = 10.2, 1.8, 1H, CH DOX), 4.26 (q, J = 6.6, 1H, CH DOX), 4.74 (ABq, J = 19.8, ν_{AB} = 15.6, 2H, CH_2 DOX), 4.99 (dt, J = 3.0, 1.2, 1H, CH DOX), 5.37 (d, J = 3.6, 1H, CH DOX), 6.70 (s, 2H, CH), 7.40 (dd, J = 7.8, 1.8, 1H, ArH DOX), 7.69 (overlapped m, 2H, ArH DOX); ^{13}C NMR (150 MHz, MeOD-d_4) 17.3 (CH_3), 26.4 (CH_2), 27.2 (CH_2), 29.2 (CH_2), 30.5 (CH_2), 34.0 (CH_2), 36.7 (CH_2), 37.2 (CH_2), 38.3 (CH_2), 47.0 (CH), 57.0 (CH_3), 65.7 (CH_2), 68.6 (CH), 69.9 (CH), 71.3 (CH), 77.3 (C), 102.3 (CH), 112.0 (ArC), 112.3 (ArC), 120.1 (ArCH), 120.4 (ArCH), 121.2 (ArC), 135.0 (ArC), 135.2 (CH), 135.6 (ArC), 136.1 (ArC), 137.1 (ArCH), 156.0 (ArC), 157.2 (ArC), 162.2 (ArC), 172.5 (CO), 175.4 (CO), 187.4 (CO), 187.7 (CO), 214.7 (CO). LRMS (ESI) 754 (100, $[M+H_2O]^+$), 759 (40, $[M+Na]^+$); HRMS (ESI) calcd. for $C_{37}H_{40}N_2O_{14}Na$ $[M+Na]^+$ 759.2377, observed: 759.2366.

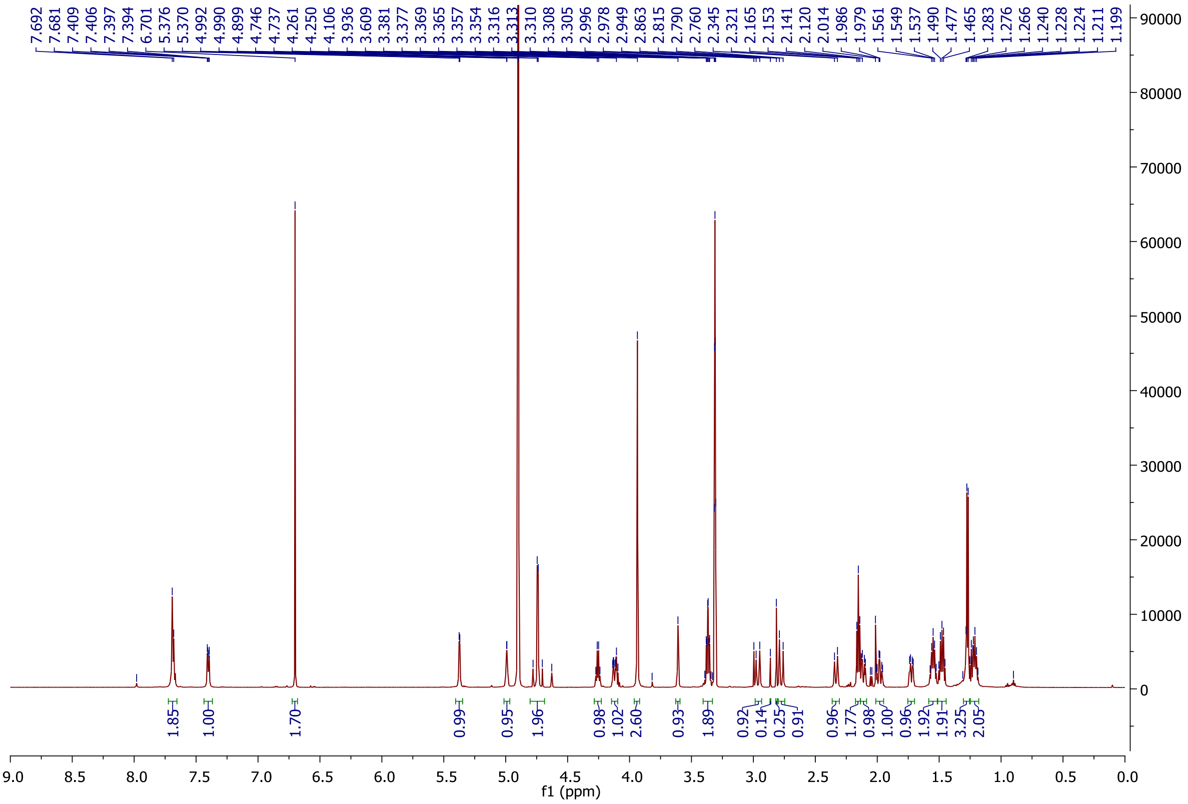
7.692 7.681 7.409 7.406 7.397 7.394 6.701 5.376 5.370 4.992 4.990 4.899 4.746 4.737 4.261 4.250 4.106 3.936 3.609 3.381 3.377 3.369 3.365 3.357 3.354 3.316 3.313 3.310 3.308 3.305 2.996 2.978 2.949 2.863 2.815 2.790 2.760 2.345 2.321 2.165 2.153 2.141 2.120 2.014 1.986 1.979 1.561 1.549 1.537 1.490 1.477 1.465 1.283 1.276 1.266 1.240 1.228 1.224 1.211 1.199
1.85 1.00 1.70 0.99 0.95 1.96 0.98 1.02 2.60 0.93 1.89 0.92 0.14 0.25 0.91 0.96 1.77 0.98 1.00 0.96 1.92 1.91 3.25 2.05
f1 (ppm)

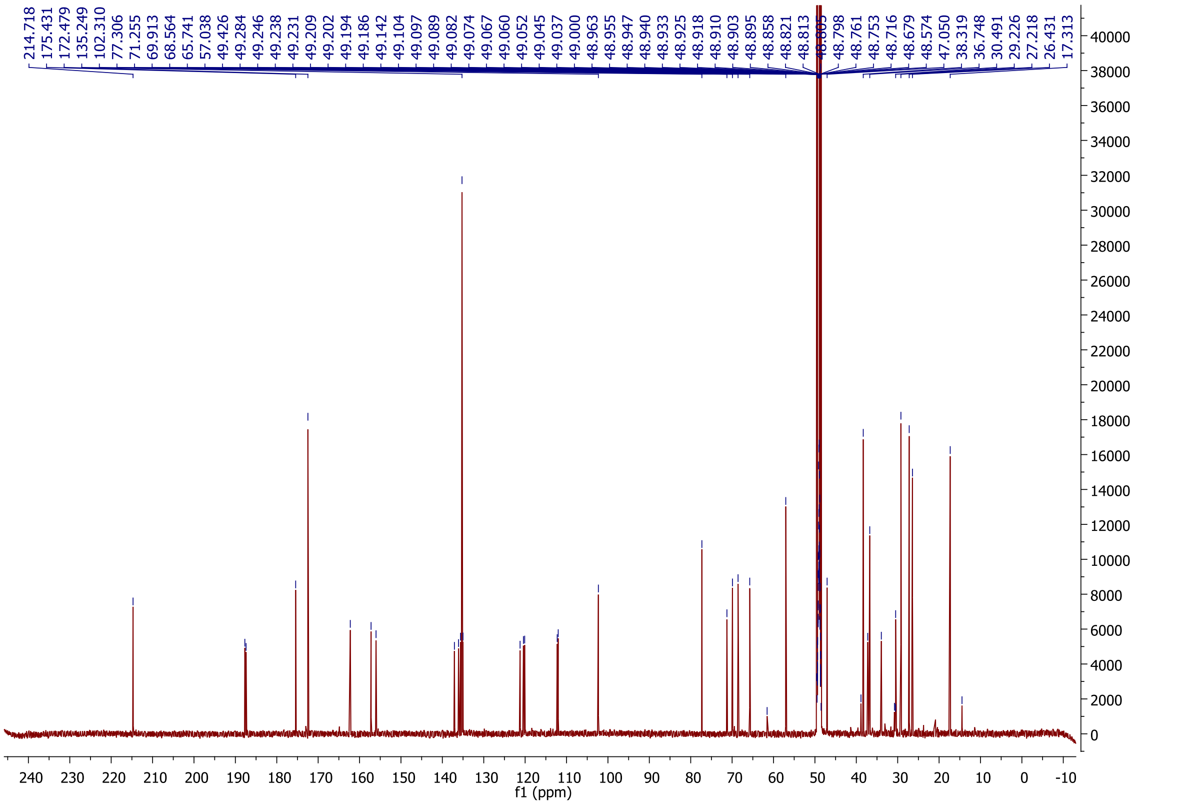
214.718 175.431 172.479 135.249 102.310 77.306 71.255 69.913 68.564 65.741 57.038 49.426 49.284 49.246 49.238 49.231 49.209 49.202 49.194 49.186 49.142 49.104 49.097 49.089 49.082 49.074 49.067 49.060 49.052 49.045 49.037 49.000 48.963 48.955 48.947 48.940 48.933 48.925 48.918 48.910 48.903 48.895 48.858 48.821 48.813 48.805 48.798 48.761 48.753 48.716 48.679 48.574 47.050 38.319 36.748 30.491 29.226 27.218 26.431 17.313
f1 (ppm)

3,4-Dithiophenoyl-maleimide-*N*-hexanamide-valine-citrulline-*p*-aminobenzyl alcohol 14

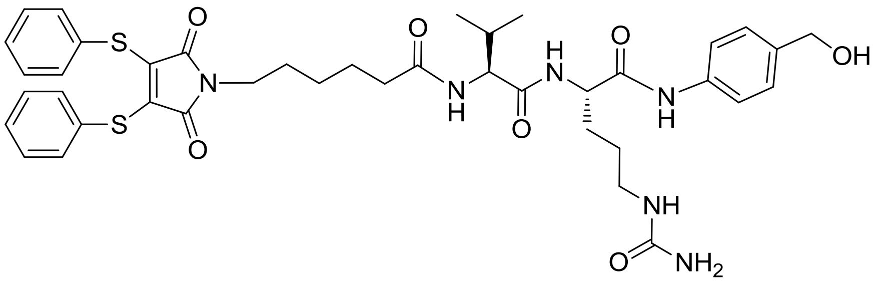

In a 5 mL round-bottom flask, under argon, compound **12** (85.7 mg, 200 μmol), HOBt (2.6 mg, 20.0 μmol, 0.10 eq.) and HBTU (75 mg, 200 μmol, 1.0 eq.) were dissolved in DMF (0.5 mL) to give a yellow solution. Next, DIPEA (37.7 μL, 220 μmol, 1.1 eq.) was added and the mixture was stirred for 3 min. Then, added valine-citrulline-*p*-aminobenzyl alcohol (val-cit-PABOH, 76.1 mg, 200 μmol, 1.0 eq.), prepared as reported,[5] and stirred at 20 °C in the dark for 5 h. Then, concentrated under vacuum, redissolved in 8:1 DCM:MeOH (90 mL) and filtered. Concentrated once more under vacuum to yield a yellow solid which was purified by flash chromatography on silica with DCM:MeOH (9:1 v/v) to afford **14** as a yellow solid (126.8 mg, 0.16 mmol, 80%). Data for **14**: no melting point, decomposes at 201-203 °C. IR (pellet) ν_{max} 3274, 2933, 2867, 1701, 1633, 1529, 1395, 1213, 1044, 1023, 736, 686. ^{1}H NMR (600 MHz, DMSO-d$_6$) 0.82-0.86 (2d, *J* = 6.6 Hz, 6H, C*H*$_3$), 1.20 (quint., *J* = 7.2 Hz, 2H, C*H*$_2$), 1.33-1.44 (2m, 2H, C*H*$_2$), 1.49 (overlapped m., 4H, C*H*$_2$), 1.55-1.70 (2m, 2H, C*H*$_2$), 1.95 (oct., *J* = 6.6 Hz, 1H, C*H*), 2.09-2.21 (2m, *J* = 7.2 Hz, 2H, C*H*$_2$), 2.92-3.01 (2m, *J* = 6.6 Hz, 2H, C*H*$_2$), 3.38 (t, *J* = 7.2 Hz, 2H, C*H*$_2$), 4.12 (ABq, *J* = 7.2, ν_{AB} = 4.3, 1H, C$_\alpha$*H*), 4.19 (ABq, *J* = 8.4, ν_{AB} = 10.8, 1H, C$_\alpha$*H*), 4.42 (d, *J* = 5.4 Hz, 2H, C*H*$_2$), 5.11 (t, *J* = 5.4 Hz, 1H, O*H*), 5.42 (br, 2H, N*H*$_2$), 5.98, (t, *J* = 5.4 Hz, 1H, N*H*), 7.21-7.30 (overlapped m, 12H, Ar*H*), 7.54 (d, *J* = 8.4 Hz, 2H, Ar*H*), 7.83 (d, *J* = 8.4 Hz, 1H, N*H*), 8.08 (d, *J* = 7.2 Hz, 1H, N*H*), 9.91 (br, 1H, N*H*); ^{13}C NMR (150 MHz, DMSO-d$_6$) 18.2 (CH_3), 19.3 (CH_3), 24.9 (CH_2), 25.3 (CH_2), 25.8 (CH_2), 26.9 (CH_2), 27.6 (CH_2), 29.4 (CH_2), 30.4 (CH), 34.9 (CH_2), 38.4 (CH_2), 53.1 (C$_\alpha$H), 57.6 (C$_\alpha$H), 62.6 (CH_2), 118.9 (ArCH), 126.9 (ArCH), 127.9 (ArCH),

129.1 (ArCH), 129.2 (ArC), 130.7 (ArCH), 135.4 (C), 137.4 (ArC), 137.5 (ArC), 158.9 (CO), 166.5 (CO), 170.4, (CO), 172.3 (CO), 172.8 (CO). LRMS (ESI) 811 (100, $[M+Na]^+$); HRMS (ESI) calcd. for $C_{40}H_{48}N_6O_7S_2Na$ $[M+Na]^+$ 811.2924, observed: 811.2917.

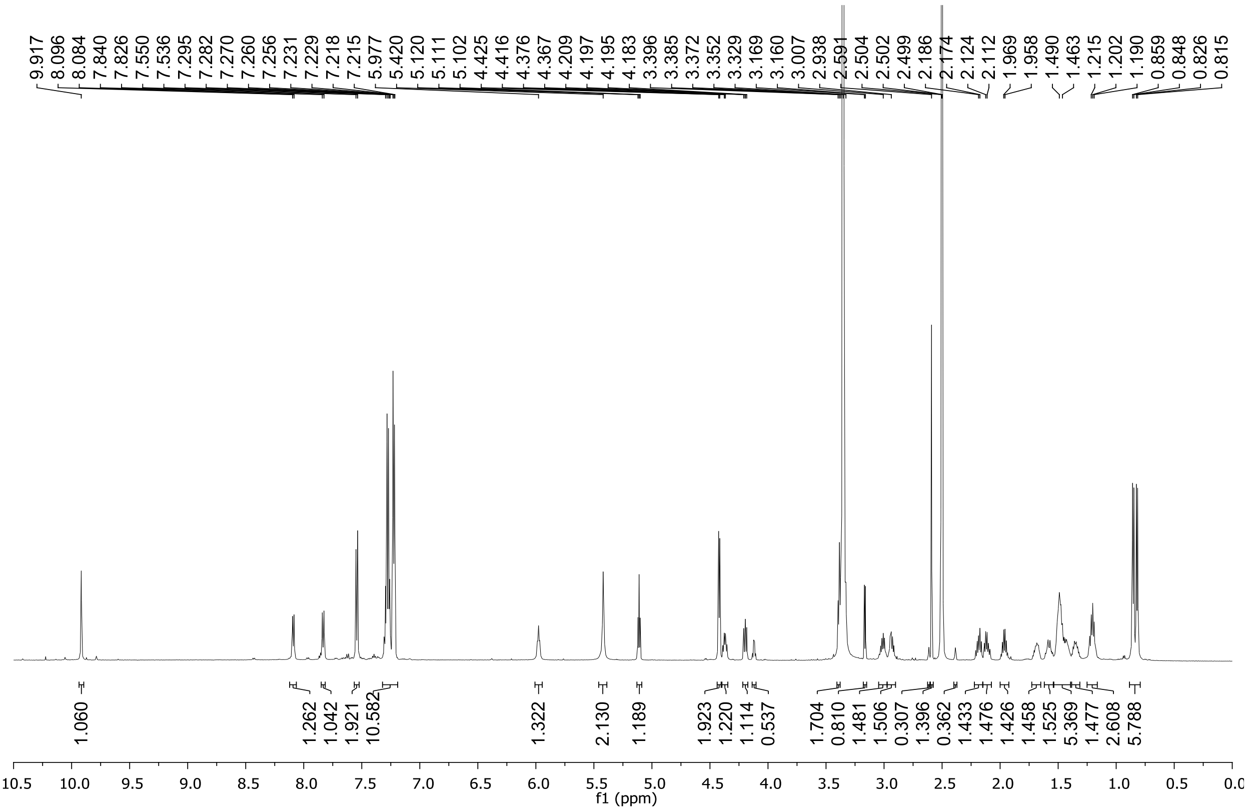

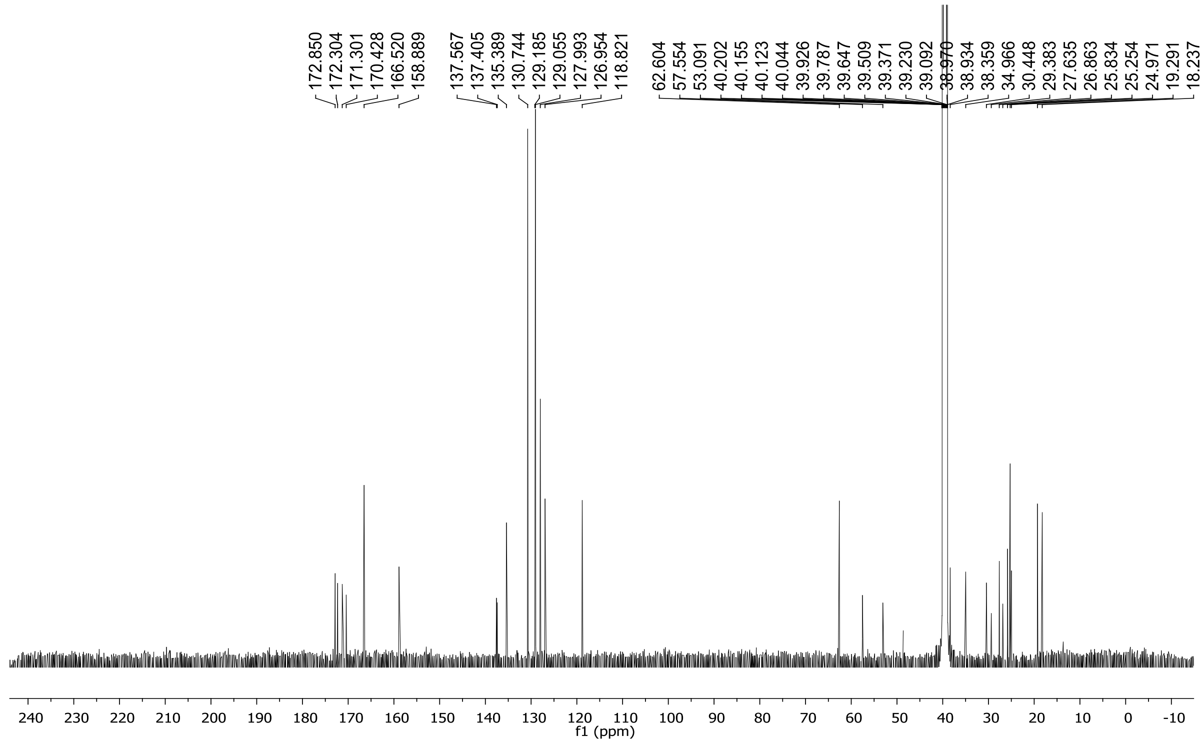

***N*-(Hexanamide-valine-citruline-*p*-aminobenzylcarbonyl-*N*-doxorubicin)-3,4-dithiophenoyl-maleimide 5**

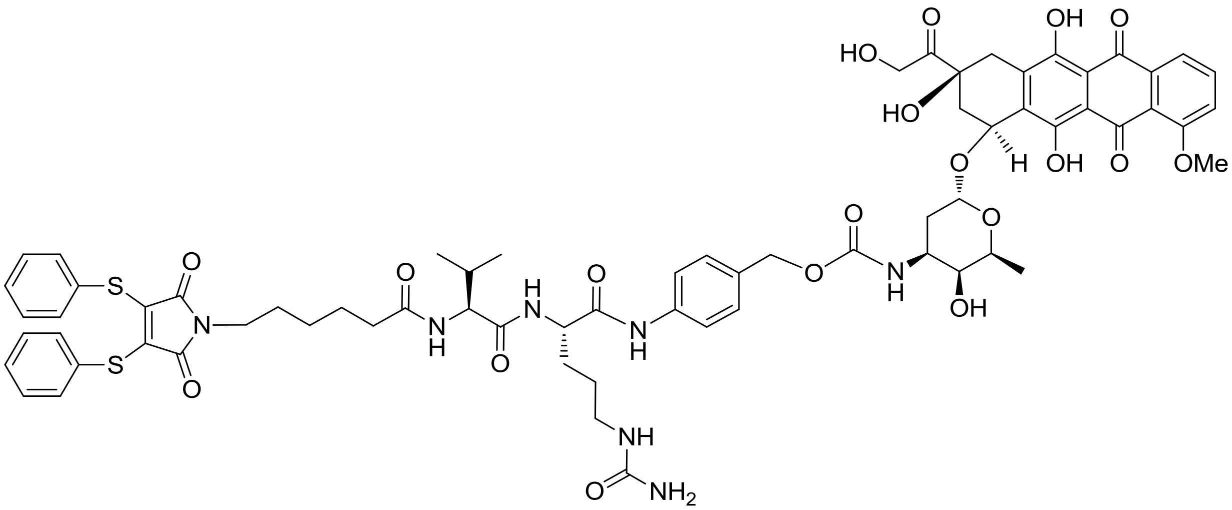

In a 10 mL round-bottom flask, under argon, compound **14** (64.1 mg, 80 μmol) was dissolved in pyridine (1.2 mL) to give a yellow solution. The solution was cooled to 0 °C and *p*-nitrophenyl-chloroformate (48.5 mg, 250 μmol, 3.0 eq.) in DCM (0.8 mL) was added. Stirred at 0 °C for 10 minutes and then allowed to warm to 20 °C and stirred for an additional 2 h. Then, added EtOAc (20 mL) and washed with 15% citric acid (3×25 mL). The organic layer was dried ($MgSO_4$), concentrated under vacuum and purified by column chromatography on silica gel 60 with a gradient of DCM:MeOH from 20:1 to 15:1 (v/v). The obtained 3,4-dithiophenoyl-maleimide-*N*-hexanamide-valine-citrulline-*p*-aminobenzyl-nitrophenyl carbonate intermediate (23.99 mg, 25 μmol, 30%) was immediately used in the next step by being dissolved under argon in DMF (1.4 mL), to which doxorubicin hydrochloride (16 mg, 27 μmol, 1.0 eq.) was added, followed by addition of DIPEA (4.8 μL, 28 μmol, 1.1 eq.). The red mixture was stirred for 16 h. Then, concentrated under vacuum (40 °C) to give a red solid which was purified by column chromatography on silica gel 60 in DCM:MeOH (10:1 v/v) to afford **5** as a red solid (33 mg, 0.24 mmol, 97%). Data for **5**: no melting point, decomposes at 134-137 °C. IR (pellet) ν_{max} 3324, 2935, 2411, 1704, 1620,

1579, 1519, 1440, 1400, 1284, 1208, 1017, 984, 736, 686. ^{1}H NMR (600 MHz, DMSO-d_6) 0.80-0.84 (2d, J = 6.6 Hz, 6H, CH_3), 1.11 (d, J = 6.6 Hz, 3H, CH_3 DOX), 1.20 (quint., J = 7.2 Hz, 2H, CH_2), 1.32-1.42 (2m, 2H, CH_2), 1.47 (overlapped m., 4H, CH_2), 1.55-1.68 (2m, 2H, CH_2), 1.83 (dt, J = 13.2, 3.6 Hz, 1H, CH_2 DOX), 1.94 (oct., J = 6.6 Hz, 1H, CH), 2.08-2.12 (m, 2H, CH_2 DOX), 2.09-2.21 (m, J = 7.8 Hz, 2H, CH_2), 2.92-3.01 (2m, J = 6.6 Hz, 2H, CH_2), 2.98 (d, J = 18 Hz, 1H, CH_2 DOX), 3.37 (m, 2H, CH_2 under water peak), 3.43 (m, 1H, CH DOX), 3.71 (m, J = 4.8 Hz, 1H, CH DOX), 3.99 (s, 3H, CH_3 DOX), 4.14 (q, J = 6.6 Hz, 1H, CH DOX), 4.18 (t, J = 7.8 Hz, 1H, $C_\alpha H$), 4.34 (q, J = 7.2 Hz, 1H, $C_\alpha H$), 4.57 (d, J = 6.0 Hz, 2H, CH_2 DOX), 4.72 (d, J = 5.4 Hz, 1H, OH DOX), 4.88 (m, 3 H, CH_2 overlapped with OH DOX), 4.94 (t, J = 4.2 Hz, 1H, CH DOX), 5.22 (d, J = 2.4 Hz, 1H, CH DOX), 5.42 (br, 2H, NH_2), 5.49 (s, 1H, OH DOX), 5.97, (t, J = 5.4 Hz, 1H, NH), 6.86 (d, J = 8.4 Hz, 1H, NH DOX), 7.21-7.29 (overlapped m, 12H, ArH), 7.54 (d, J = 8.4 Hz, 2H, ArH), 7.67 (dd, J = 6.0, 3.0 Hz, 1H, ArH DOX), 7.82 (d, J = 8.4 Hz, 1H, NH), 7.92 (m, 2H, ArH DOX), 8.09 (d, J = 7.2 Hz, 1H, NH), 9.97 (br, 1H, NH), 13.30 (br, 1H, OH DOX), 14.05 (br, 1H, OH DOX); ^{13}C NMR (150 MHz, DMSO-d_6) 17.1 (CH_3), 18.2 (CH_3), 19.3 (CH_3), 24.9 (CH_2), 25.8 (CH_2), 26.8 (CH_2), 27.6 (CH_2), 29.3 (CH_2), 29.8 (CH_2), 30.4 (CH), 30.7 (CH_2), 32.1 (CH_2), 34.9 (CH_2), 36.7 (CH_2), 38.2 (CH_2), 47.1 (CH), 53.1 ($C_\alpha H$), 56.6 (CH_3), 57.5 ($C_\alpha H$), 63.7 (CH_2), 64.9 (CH_2), 66.4 (CH), 67.9 (CH), 69.9 (CH), 74.9 (C), 100.3 (CH), 110.7 (ArC), 110.9 (ArC), 118.9 (ArCH), 119.1 (ArCH), 119.8 (ArCH), 120.1 (ArC), 128.0 (ArCH), 128.6 (ArCH), 129.0 (ArCH), 129.2 ArC), 130.7 (ArCH), 131.8 (ArC), 134.2 (ArC), 134.7 (ArC), 135.4 (C), 135.6 (ArC), 136.3 (ArCH), 154.5 138.5 (ArC), (ArC), 154.5 (ArC), 155.3 (ArC), 156.1 (CO), 158.9 (CO), 160.8 (ArC), 166.5 (CO), 170.6, (CO), 171.3 (CO), 172.3 (CO), 186.6 (CO), 186.7 (CO), 213.9 (CO). LRMS (ESI) 1380 (62, $[M+Na]^+$); HRMS (ESI) calcd. for $C_{68}H_{75}N_7O_{19}S_2Na$ $[M+Na]^+$ 1380.4457, observed: 1380.4388.

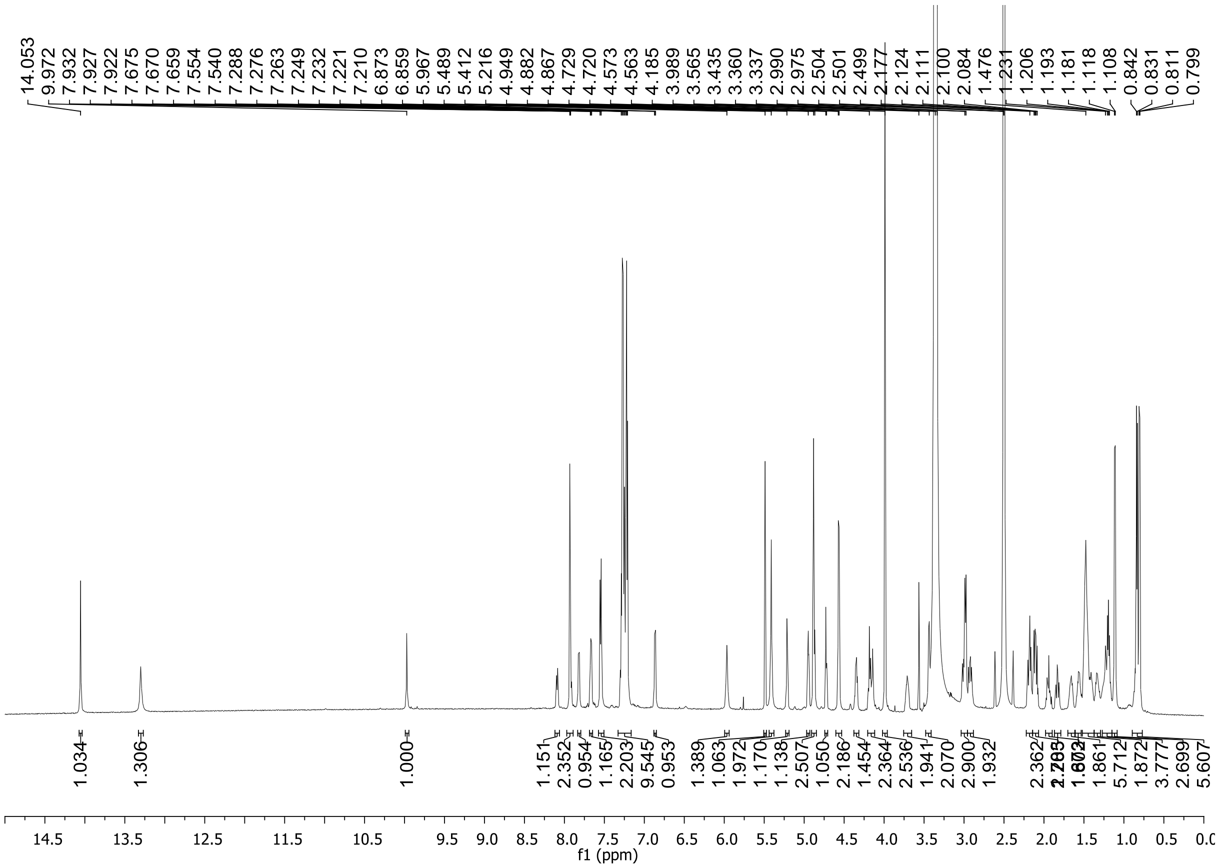
14.053
9.972
7.932
7.927
7.922
7.675
7.670
7.659
7.554
7.540
7.288
7.276
7.263
7.249
7.232
7.221
7.210
6.873
6.859
5.967
5.489
5.412
5.216
4.949
4.882
4.867
4.729
4.720
4.573
4.563
4.185
3.989
3.565
3.435
3.360
3.337
2.990
2.975
2.504
2.501
2.499
2.177
2.124
2.111
2.100
2.084
1.476
1.231
1.206
1.193
1.181
1.118
1.108
0.842
0.831
0.811
0.799
1.034
1.306
1.000
1.151
2.352
0.954
1.165
2.203
9.545
0.953
1.389
1.063
1.972
1.170
1.138
2.507
1.050
2.186
1.454
2.364
2.536
1.941
2.070
2.900
1.932
2.362
1.861
5.712
1.872
3.777
2.699
5.607
14.5 13.5 12.5 11.5 10.5 9.5 9.0 8.5 8.0 7.5 7.0 6.5 6.0 5.5 5.0 4.5 4.0 3.5 3.0 2.5 2.0 1.5 1.0 0.5 0.0
f1 (ppm)

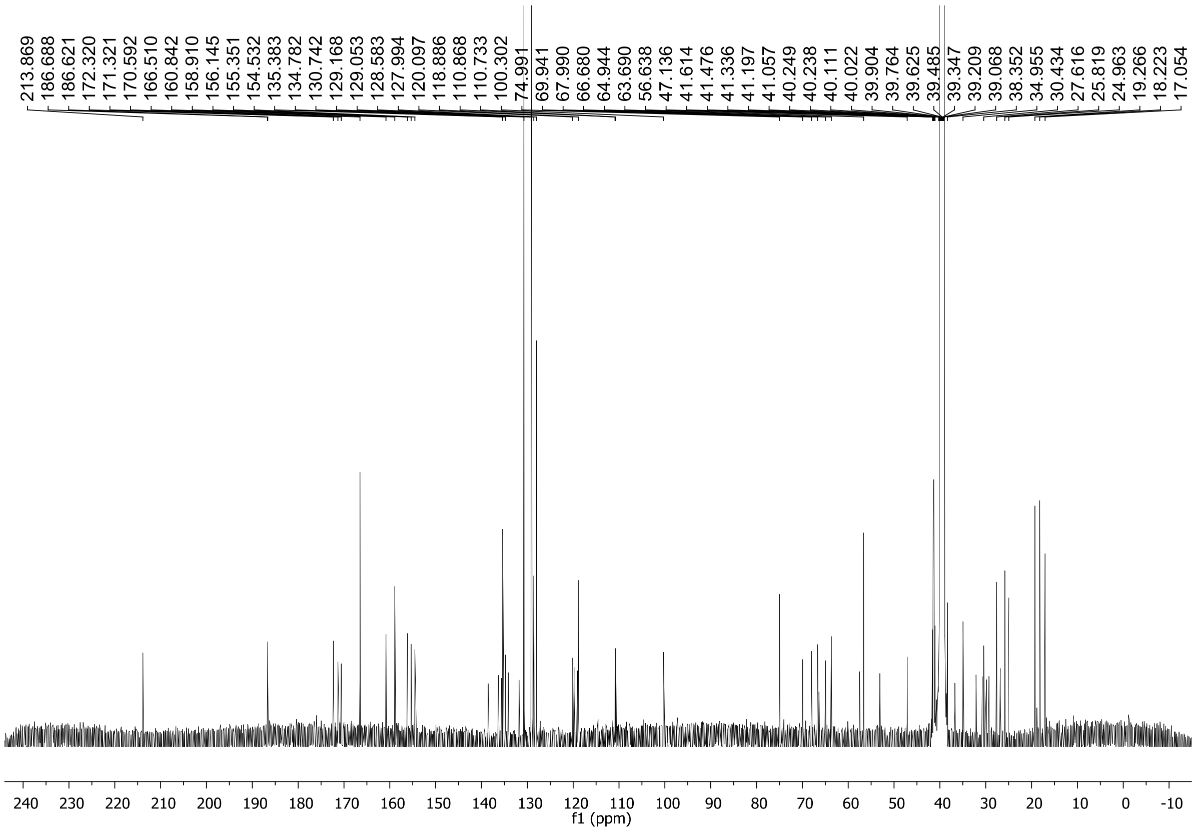
213.869
186.688
186.621
172.320
171.321
170.592
166.510
160.842
158.910
156.145
155.351
154.532
135.383
134.782
130.742
129.168
129.053
128.583
127.994
120.097
118.886
110.868
110.733
100.302
74.991
69.941
67.990
66.680
64.944
63.690
56.638
47.136
41.614
41.476
41.336
41.197
41.057
40.249
40.238
40.111
40.022
39.904
39.764
39.625
39.485
39.347
39.209
39.068
38.352
34.955
30.434
27.616
25.819
24.963
19.266
18.223
17.054
240 230 220 210 200 190 180 170 160 150 140 130 120 110 100 90 80 70 60 50 40 30 20 10 0 -10
f1 (ppm)

N-Propargyl-3,5-dithiophenoyl-maleimide 15[6]

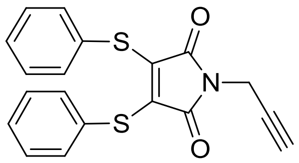

This compound was prepared as reported.[6] To a solution of *N*-methoxycarbonyl-3,4-dithiophenoylmaleimide (50 mg, 140 µmol), prepared as reported,[6] in DCM (6 mL) was added propargylamine (9 µL, 140 µmol, 1.0 eq.). The mixture was stirred at 20 °C for 2 h. Then, silica was added and the resulting mixture was stirred overnight. Next, the mixture was filtered and concentrated under vacuum to yield a yellow oil that was purified by column chromatography on silica gel 60 in petroleum ether:EtOAc (10:1 to 6:4 v/v) to afford **15** as a yellow oil (46.5 mg, 132 µmol, 98%). Data for **15** in accordance with reported:[6] m.p. 119-120 °C. IR (pellet) ν_{max} (cm^{-1}) 3285, 1775, 1707, 1422, 1393, 1341, 1118, 936, 730, 683. ^{1}H NMR (600 MHz, $CDCl_3$) 2.21 (t, J = 2.3 Hz, 1H, C*H*), 4.26 (d, J = 2.3 Hz, 2H, CH_2), 7.22 (d, J = 7.2 Hz, 4H, Ar*H*), 7.26 (t, J = 7.2 Hz, 4H, Ar*H*), 7.30 (t, J = 7.2 Hz, 2H, Ar*H*); ^{13}C NMR (150 MHz, $CDCl_3$) 27.7 (CH_2), 71.9 (CH), 76.9 (C), 128.7 (ArCH), 128.8 (ArC), 129.1 (ArCH), 131.9 (ArCH), 136.0 (C), 165.6 (CO). LRMS (ESI) 351 (100, $[M]^+$); HRMS (ESI) calcd. for $C_{19}H_{13}O_2NS_2$ $[M]^+$ 351.03822, observed: 351.03865.

***N*-Doxorubicin-14-azido-3,6,9,12-tetraoxatetradecan-1-amide 16**

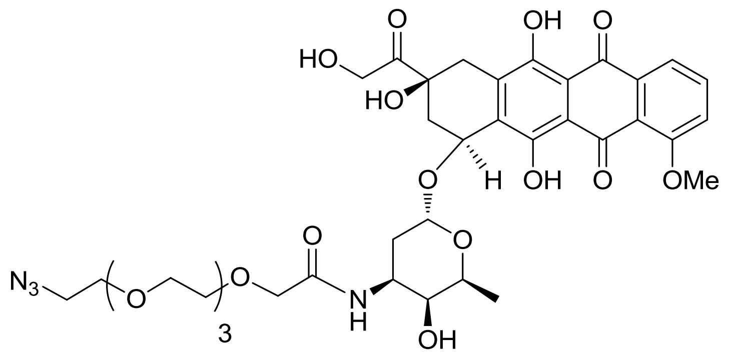

In a 10 mL round-bottom flask under argon, 2-(2-(2-(2-azidoethoxy)ethoxy)ethoxy)ethanamine (23.9 mg, 86 µmol, 1.0 eq.), prepared as reported,[7] HOBt (1.25 mg, 9.5 µmol, 0.11 eq.) and HBTU (6.7 mg, 89 µmol, 1.0 eq.) were dissolved in DMF (2 mL). Next, added DIPEA (16.4 µL, 95 µmol, 1.1 eq.) and the mixture was stirred for 5 min. Then, a solution of doxorubicin hydrochloride (50 mg, 86 µmol, 1 eq.) with DIPEA (16.4 µL, 95 µmol, 1.1 eq.) in DMF (2 mL) was added. The solution turned red upon addition. The solution was stirred at 20 °C for 6 h. Then, removed DMF by concentrating under vacuum in the presence of excess toluene (2×40 mL). Next, added DCM (50 mL) and MeOH (8 mL) and washed with 0.68 M acetate buffer pH 5 (2×10 mL) and aq. saturated LiCl solution (2×20 mL). The organic layer was dried ($MgSO_4$), filtered and concentrated under vacuum to yield a red solid which was purified by flash chromatography on silica with DCM:EtOAc:MeOH (20:20:1 v/v) to afford **9** as a red solid (25.7 mg, 32 µmol, 37%). Data for **9**: mp = 61-64 °C. IR (pellet) ν_{max} 3405, 3341, 2917, 2100, 1650, 1615, 1578, 1408, 1281, 1207, 1109, 1080, 980, 791. ^{1}H NMR (600 MHz, MeOD-d_4 + drops of $CDCl_3$) 1.27 (d, J = 6.6 Hz, 3H, CH_3 DOX), 1.75-1.78 (dd, J = 13.2, 4.8 Hz, 1H, CH_2 DOX), 1.92 (dt, J = 13.2, 4.2 Hz, 1H, CH_2 DOX), 2.12-2.15 (dd, J = 14.4, 4.2 Hz, 1H, CH_2 DOX), 2.33 (d, J = 14.4, 1H, CH_2 DOX), 2.90 (d, J = 18.6, 1H, CH_2 DOX), 3.18 (d, J = 18.6, 1H, CH_2 DOX), 3.39 (t, J = 5.4, 2H, N_3CH_2), 3.62-3.70 (overlapped m, 15H, CH_2O overlapped with CH DOX), 3.91 (ABq, J = 16.2, ν_{AB} = 16.9, 2H, OCH_2CO), 4.05 (s, 3H, CH_3 DOX), 4.11 (q, J =

6.6, 1H, C*H* DOX), 4.17 (m, 1H, C*H* DOX), 4.74 (ABq, *J* = 21.0, ν_{AB} = 8.9, 2H, CH_2 DOX), 5.24 (dt, *J* = 3.6, 1.8, 1H, C*H* DOX), 5.48 (d, *J* = 4.2, 1H, C*H* DOX), 7.24 (br, 1H, N*H* DOX), 7.36 (d, *J* = 8.4, 1H, Ar*H*, DOX), 7.74 (t, *J* = 7.8, 1H, Ar*H* DOX), 7.97 (dd, *J* = 7.8, 1.2, 1H, Ar*H* DOX); ^{13}C NMR (150 MHz, MeOD-d_4 + drops of $CDCl_3$) 17.1 (CH_3), 29.7 (CH_2), 34.0 (CH_2), 35.7 (CH_2), 45.0 (CH), 50.7 (CH_2), 56.8 (CH_3), 65.7 (CH_2), 67.6 (CH), 69.2 (CH), 69.7 (CH), 70.1 (CH_2O), 70.2 (CH_2O), 70.4 (CH_2O), 70.5 (CH_2O), 70.6 (CH_2O), 70.8 (CH_2O), 71.0 (CH_2O), 76.7 (C), 101.1 (CH), 111.4 (ArC), 111.5 (ArC), 118.5 (ArCH), 119.9 (ArCH), 120.8 (ArC), 133.7 (ArC), 133.8 (ArC), 135.5 (ArC), 135.8 (ArCH), 155.7 (ArC), 156.3 (ArC), 161.1 (ArC), 169.4 (CO), 186.6 (CO), 187.0 (CO), 214.1 (CO). LRMS (ESI) 825 (100, $[M+Na]^+$); HRMS (ESI) calcd. for $C_{37}H_{46}N_4O_{16}Na$ $[M+Na]^+$ 825.2808, observed: 825.2807.

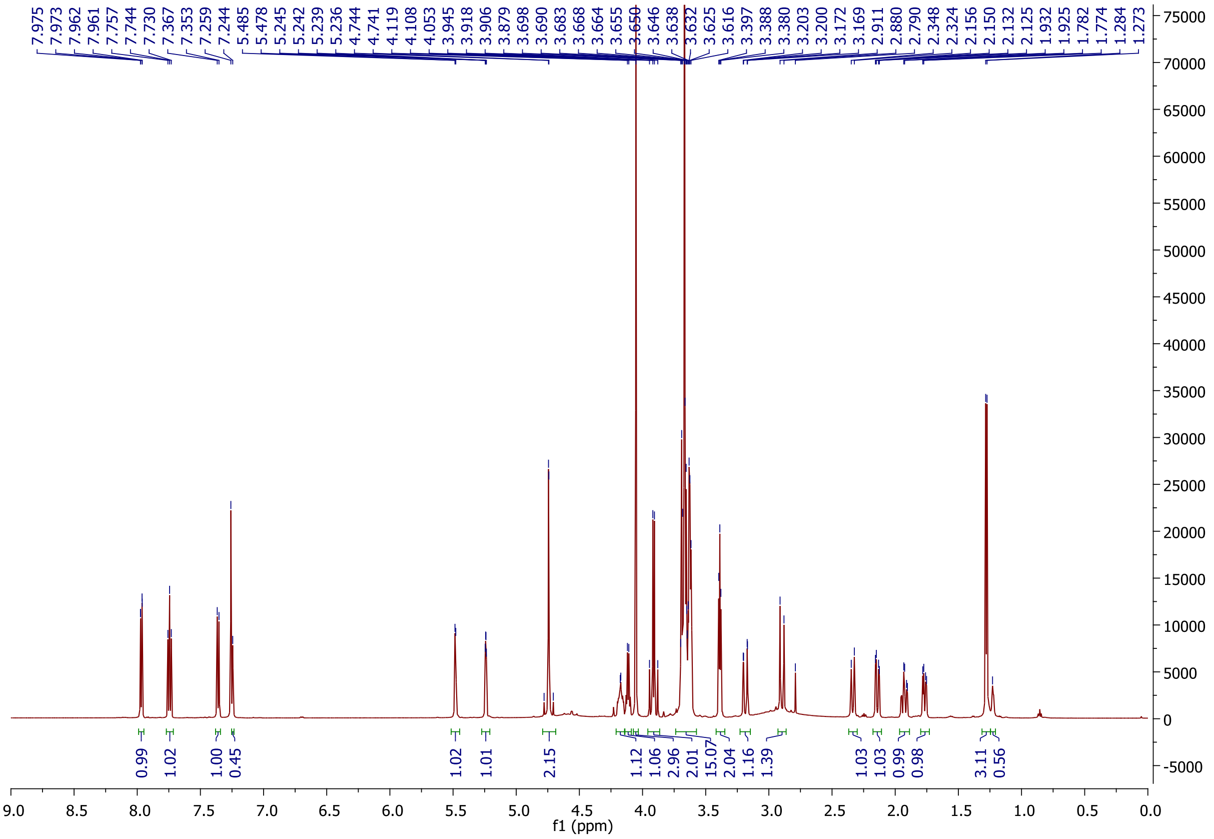

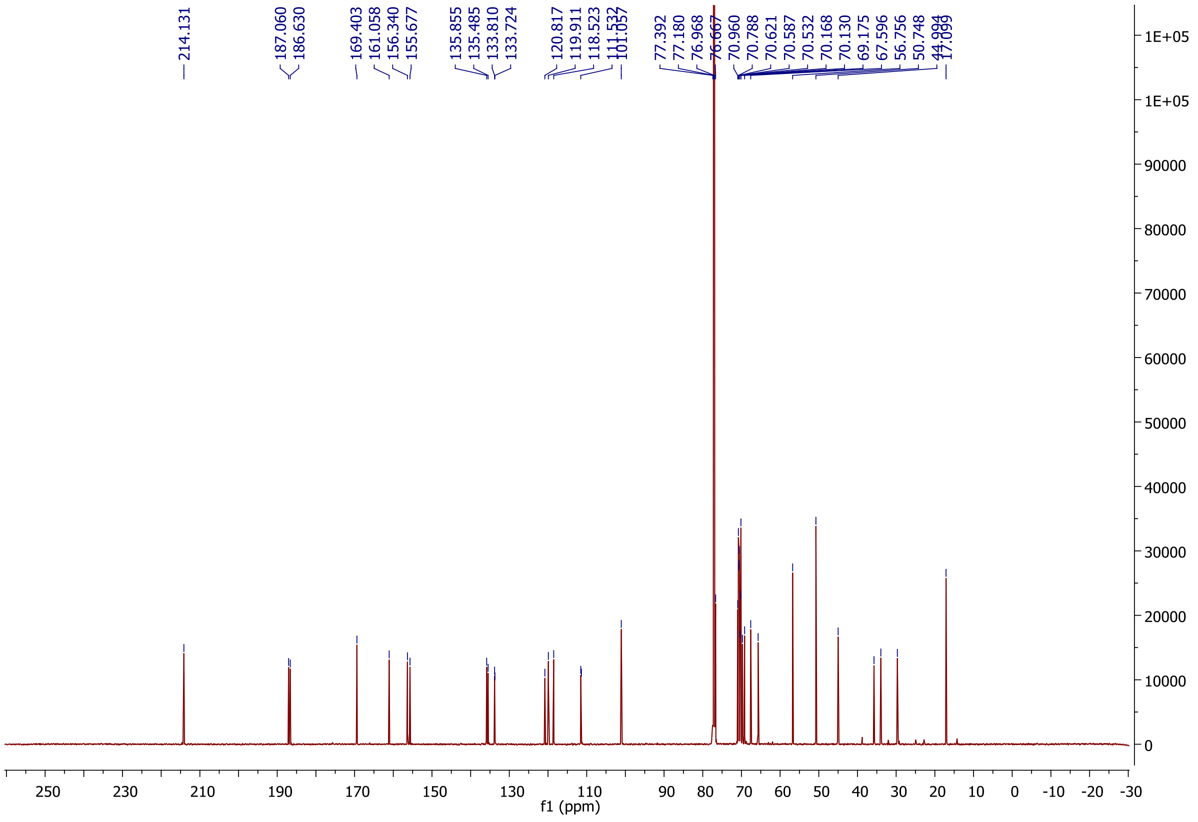
214.131
187.060
186.630
169.403
161.058
156.340
155.677
135.855
135.485
133.810
133.724
120.817
119.911
118.523
111.532
101.057
77.392
77.180
76.968
76.667
70.960
70.788
70.621
70.587
70.532
70.168
70.130
69.175
67.596
56.756
50.748
44.994
17.099
f1 (ppm)

Conjugation Experiments General Remarks

Conjugation experiments were carried out in standard polypropylene micro test tubes 3810x at atmospheric pressure with mixing at 20 °C unless otherwise stated. Reagents and solvents were purchased from commercial sources and used as supplied. All buffer solutions were prepared with double-deionised water and filter-sterilised. Borate buffer was 25 mM Sodium Borate, 25 mM NaCl and 1 mM EDTA at pH 8. Phosphate-buffered saline (PBS) was 140 mM NaCl and 12 mM phosphates at pH 7.4 Ultrapure DMF was purchased from Sigma-Aldrich and kept under dry conditions. Solutions of tris(2-carboxyethyl)phosphine hydrochloride (TCEP) 22.9 mM (6.56 mg/mL) were prepared in borate buffer. Solutions of benzeneselenol 22.9 mM (2.44 μL/mL) were prepared in DMF under argon. Opened bottles of benzeneselenol were kept under argon and replaced when the solution had turned orange.

Ultrafiltration was carried out in vivaspin 500 polyethersulfone (PES) membrane concentrators with a molecular weight cut-off (MWCO) of 10 kDa. Centrifugation was carried out on an eppendorf 5415R fixed angle rotor centrifuge operating at 14000 rcf at 20 °C. Purification by size exclusion chromatography (SEC) was carried out on an ÄKTA Purifier HPLC system (GE Healthcare), equipped with a HiLoad Superdex 75 16/60 column (GE Healthcare) equilibrated in PBS, running at a flow rate of 0.5 mL/min. Detection was by absorption at 280 nm.

Trastuzumab is a chimeric IgG1 full length antibody directed against HER2. The antibody was obtained in its clinical formulation (Roche, lyophilised). The powder was dissolved in 10 ml sterile water and the buffer exchanged completely for digest buffer (50 mM phosphate, 1 mM EDTA, pH 6.8) *via* ultrafiltration (MWCO 50 kDa, Sartorius). The concentration after the exchange was determined by UV-vis absorbance and adjusted to 22.9 μM (3.37 mg/mL) and was stored in flash frozen aliquots at –20 °C. For experiments, aliquots were thawed; buffer swapped into the buffer of choice and concentration was corrected to

22.9 μM (3.37 mg/mL). The following acronyms are used to describe antibody fragments based on their constituent heavy and light chains: heavy-heavy-light (HHL), heavy-heavy (HH), heavy-light (HL, a.k.a. half antibody), heavy (H) and light (L) chains.

Analytical methods for antibody-drug conjugates

SDS-PAGE

Non-reducing glycine-SDS-PAGE at 12% acrylamide gels were performed following standard lab procedures. A 4% stacking gel was used and a broad-range MW marker (10-250 kDa, BioLabs) was co-run to estimate protein weights. Samples (3-5 μL at ~22.9 μM in total mAb) were quenched with maleimide (1 μL of a 11.5 mM solution in PBS, 100 eq.) and mixed with loading buffer (1-2 μL, composition for 6× SDS: 1 g SDS, 3 mL glycerol, 6 mL 0.5 M Tris buffer pH 6.8, 2 mg R-250 dye) and heated at 65 °C for 2 minutes. The gel was run at constant current (30-35 mA) for 40 min in 1× SDS running buffer. All gels were stained following a modified literature protocol[8] where 0.12 % of the Coomassie G-250 and the Coomassie R-250 dyes were added to the staining solution (5:4:1 MeOH:H_2O:AcOH) instead of only the G-250 dye. Gel photographs were taken with a UVP BioDoc-it imaging system.

Determination of drug to antibody ratio (DAR)

UV-vis spectra were recorded on a Varian Cary 100 Bio UV-visible spectrophotometer, operating at 20 °C. Sample buffer was used as blank for baseline correction. Calculation of DAR follows the formula below with ε_{280} = 215380 M^{-1} cm^{-1} for trastuzumab mAb, ε_{280} = 68560 M^{-1} cm^{-1} for trastuzumab Fab, ε_{495} = 8030 M^{-1} cm^{-1} for doxorubicin and 0.724 as a correction factor for DOX absorption at 280 nm. DAR was calculated as follows:

$$\text{DAR} = \frac{Abs_{495}/\varepsilon_{495}}{\left(Abs_{280} - 0.724 \times Abs_{495}\right)/\varepsilon_{280}}$$

Ellman's assay

Ellman's assay was carried out by mixing a 1 mM solution of 5,5'-dithio-*bis*-(2-nitrobenzoic acid) (Ellman's reagent) in PBS (9.5 μL) with the sample at 22.9 μM (20 μL) and diluting with PBS (50.5 μL). The solution was incubated at 20 °C for 2 min and then absorption was measured at 280 nm (protein concentration) and 412 nm (2-nitro-5-thiobenzoic acid). A sample of 1 mM Ellman's reagent in PBS (9.5 μL) diluted with PBS (70.5 μL) was used as blank for baseline correction. Each sample in PBS was analysed in the absence and presence of Ellman's reagent, under identical concentration conditions. The absorption of DOX at 412 nm in the absence of Ellman's reagent was subtracted from the absorption at 412 nm in the presence of Ellman's reagent. The sulfhydryl per protein ratio (SPR) was calculated as follows with ε_{412} = 14150 M^{-1} cm^{-1}:

$$\text{SPR} = \frac{\left(Abs_{412} - Abs_{412}\left(DOX\right)\right)/\varepsilon_{412}}{Abs_{280}/\varepsilon_{280}}$$

Liquid chromatography mass spectrometry (LCMS)

Samples (22.9 μM) were diluted 5 times with water before LCMS analysis. LCMS was performed on Fab samples using a Thermo Scientific uPLC connected to MSQ Plus Single Quad Detector (SQD). Column: Hypersil Gold C4, 1.9 μm 2.1 × 50 mm. Wavelength: 254 nm. Mobile Phase: 99:1 Water:MeCN (0.1% formic acid) to 1:9 Water: MeCN (0.1% formic acid) gradient over 4 min. Flow Rate: 0.3 mL/min. MS Mode: ES+. Scan Range: m/z = 500-2000. Scan time: 1.5 s. Data obtained in continuum mode. The electrospray source of the MS was operated with a capillary voltage of 3.5 kV and a cone voltage of 50 V. Nitrogen was used as the nebulizer and desolvation gas at a total flow of 600 L/h. Ion series were generated by integration of the total ion

chromatogram (TIC) over the 3.5-5.0 min range. Total mass spectra for protein samples were reconstructed from the ion series using the pre-installed ProMass software.

Enzyme-linked immunosorbent assay (ELISA)

Binding affinity to HER2 receptor was determined by ELISA. A 96-well plate was coated for 2 h at 20 °C with HER2 (100 µL of a 0.25 µg/mL solution in PBS). One row of wells was coated with PBS only as a negative control. Next, each well was washed with PBS and blocked with a 1% BSA solution in PBS (200 µL) overnight at 4 °C. Then, the wells were washed with 0.1% Tween 20 in PBS followed by PBS. Trastuzumab and respective ADCs were diluted in PBS yielding the following concentrations: 30 nM, 10 nM, 3.33 nM, 1.11 nM, 0.37 nM and 0.12 nM. The dilution series was added, including PBS only and ADC at 30 nM in the absence of HER2 as negative controls. The plate was incubated for 2 h at 20 °C. Then, wells were washed and the detection antibody (100 µL of anti-human IgG, Fab-specific-HRP solution, 1:5000 in PBS) was added followed by incubation for 1 h at 20 °C. After another washing step, freshly prepared OPD solution (100 µL of 10 mg/20 mL OPD in phosphate-citrate buffer) was added to each well and the reaction was stopped by addition of 4 M HCl (50 µL). The colorimetric reaction was measured at 490 nm and the absorption was corrected by subtracting the average of negative controls. Each measurement was done in triplicates.

Trastuzumab modification protocols

Conjugation of trastuzumab with NGM-DOX 3-5 by sequential protocol

To trastuzumab (22.9 µM, 300 µL, 0.0069 µmol) in borate buffer was added TCEP (22.9 mM, 1.8 µL, 6 eq.) and the reaction was incubated at 37 °C for 2 h under mild agitation. Next, NGM-DOX compound was prepared in dry DMF (9.16 mM) and added to the reduced trastuzumab (3.8 µL, 5 eq.). The concentration of DMF was corrected to 10% (v/v) and the reaction was incubated at 37 °C for 1 h. Afterwards, excess reagents were removed by ultrafiltration (10 kDa MWCO) with PBS to afford the modified trastuzumab ADC in PBS. Yields and DAR are quoted for each reagent. NGM-DOX **3**: yield 91%, DAR 3.8; NGM-DOX **4**: yield 99%, DAR 3.0; NGM-DOX **5**: yield 90%, DAR 3.5.

Conjugation of trastuzumab with M-DOX 6 by sequential protocol

To trastuzumab (22.9 µM, 300 µL, 0.0069 µmol) in borate buffer was added TCEP (22.9 mM, 0.6 µL, 2 eq.) and the reaction was incubated at 37 °C for 2 h under mild agitation. Next, M-DOX **6** was prepared in dry DMF (9.16 mM) and added to the reduced trastuzumab (1.5 µL, 2 eq.). The concentration of DMF was corrected to 10% (v/v) and the reaction was incubated at 37 °C for 1 h. Afterwards, excess reagents were removed by ultrafiltration (10 kDa MWCO) with PBS to afford the modified trastuzumab ADC in PBS with yield 89%, DAR 3.7.

Conjugation of trastuzumab with NGM-DOX 3-5 by *in situ* protocol

To trastuzumab (22.9 µM, 300 µL, 0.0069 µmol) in borate buffer was added a 9.16 mM solution of NGM-DOX compound (3.8 µL, 5 eq.) in DMF and the amount of DMF was

corrected to 10% (v/v). Next, added TCEP (22.9 mM, 1.8 μL, 6 eq.) and the reaction was incubated at 37 °C for 2 h under mild agitation. Afterwards, excess reagents were removed by ultrafiltration (10 kDa MWCO) with PBS to afford the modified trastuzumab ADC in PBS. Yields and DAR are quoted for each reagent. NGM-DOX **3**: yield 89%, DAR 3.4; NGM-DOX **4**: yield 98%, DAR 1.8; NGM-DOX **5**: yield 95%, DAR 3.1.

Conjugation of trastuzumab with compound 15 by *in situ* protocols, followed by copper-catalysed Huisgen 1,3-dipolar cycloaddition with compound 16

Trastuzumab (22.9 μM, 250 μL, 0.00573 μmol) in borate buffer was treated with one of the following protocols for targeting a specific DAR:

DAR 1: Added DMF to a concentration of 12% (v/v) and the mixture was put on ice. Next, added benzeneselenol freshly prepared in dry DMF (22.9 mM, 2.5 μL, 10 eq.), followed by addition of NGM **15** in dry DMF (9.16 mM, 12.5 μL, 20 eq.) and corrected DMF to a concentration of 15% (v/v). The reaction was kept on ice for 1 h and purified by ultrafiltration (10 kDa MWCO). Yield 84%.

DAR 2: Added DMF to a concentration of 12% (v/v) and the mixture was put on ice. Next, added benzeneselenol freshly prepared in dry DMF (22.9 mM, 2.5 μL, 10 eq.), followed by addition of NGM **15** in dry DMF (9.16 mM, 12.5 μL, 20 eq.) and corrected DMF to a concentration of 15% (v/v). The reaction was kept on ice for 30 minutes, after which added benzeneselenol freshly prepared in dry DMF (22.9 mM, 2.5 μL, 10 eq.). The reaction was kept on ice for another 30 minutes and purified by ultrafiltration (10 kDa MWCO). Yield 86%.

DAR 3: Added NGM **15** in dry DMF (9.16 mM, 6.3 μL, 10 eq.) and corrected DMF to a concentration of 15% (v/v), followed by addition of TCEP in borate buffer (22.9 mM, 1.8 μL,

7 eq.). The reaction was incubated at 37 °C for 2 h under mild agitation after which the reaction was purified by ultrafiltration (10 kDa MWCO). Yield 82%.

DAR 4: Added NGM **15** in dry DMF (9.16 mM, 9.4 µL, 15 eq.) and corrected DMF to a concentration of 15% (v/v), followed by addition of TCEP in borate buffer (22.9 mM, 2.5 µL, 10 eq.). The reaction was incubated at 37 °C for 2 h under mild agitation after which the reaction was purified by ultrafiltration (10 kDa MWCO). Yield 86%.

Copper-catalysed Huisgen 1,3-dipolar cycloaddition: Bridging reaction was stopped for all protocols by addition of maleimide in PBS (22.9 mM, 5µL, 20 eq.) and purified by ultrafiltration (10 kDa MWCO) into PBS. After determination of the concentration by UV-vis and dilution of the antibody to 30 µM in PBS, reactions were treated with compound **16** in dry DMF (12 mM 30 eq.), in the presence of $CuSO_4$ (150 µM), THPTA (750 µM), aminoguanidine·HCl (5 mM) and sodium ascorbate (5 mM). Reactions were incubated at 22 °C for 90 min (for DAR 1) or 16 h (for DARs 2-4). ADCs were purified by size exclusion chromatography (on a HiLoad Superdex 75 16/60 column, GE Healthcare, equilibrated in PBS). Yields for the cycloaddition reactions and DARs after size exclusion chromatography are quoted for each protocol targeting a specific DAR: **DAR 1**: yield 82%, DAR 1.1; **DAR 2**: yield 72%, DAR 2.0; **DAR 3**: yield 69%, DAR 3.1; **DAR 4**: yield 60%, DAR 4.0.

<u>**Supplementary figures and tables**</u>

Figure S1 – Optimization of the sequential bridging protocol

a)

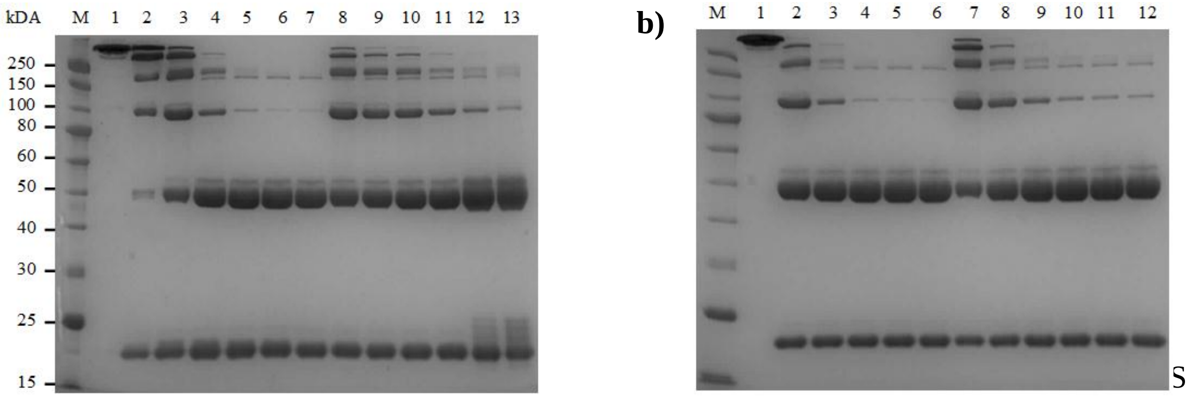

c)

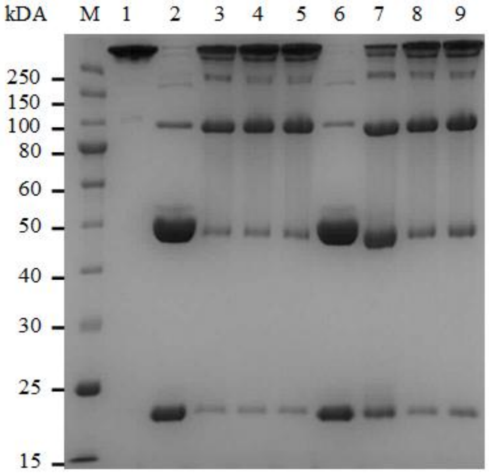

d)

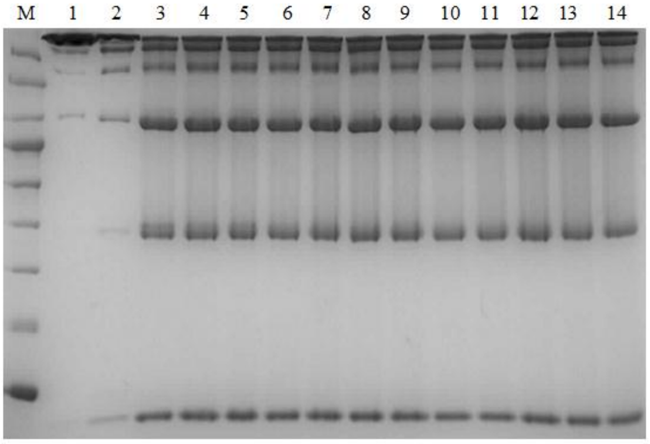

Figure S1 – Optimization of the sequential bridging of trastuzumab. All reactions were carried out as described for the general procedure with the here mentioned exceptions. **a)** Reduction in 50 mM sodium phosphate, 150 mM NaCl, 1 mM EDTA, pH 6.8 for 2 h at 37 °C. M) Molecular weight marker. 1) Untreated antibody. 2) 2.8, 3) 4, 4) 8, 5) 12, 6) 16 and 7) 20 eq. TCEP. 8) 16, 9) 20, 10) 24, 11) 32, 12) 40 and 50 eq. DTT. **b)** Reduction in 25 mM sodium borate, 25 mM NaCl, 1 mM EDTA, pH 8.0 for 2 h at 37 °C. M) Molecular weight marker. 1) Untreated antibody. 2) 4, 3) 5, 4) 6, 5) 7 and 6) 8 eq. TCEP. 7) 4, 8) 6, 9) 8, 10) 10, 11) 12 and 12) 14 eq. DTT. **c)** Bridging under different conditions. M) Molecular weight marker. 1) Untreated antibody. 2) Reduced antibody. 3) Bridging with 4, 4) 12 and 5) 20 eq. *N*-methyl-dibromomaleimide in pH 8.0. 6) Reduced antibody. 7) Bridging with 4, 8) 12 and 9) 20 eq. *N*-methyl-dibromomaleimide in pH 6.0. **d)** Bridging for different times. All reactions were stopped after the indicated time with an excess of maleimide. M) Molecular weight marker. 1) Untreated antibody. 2) Boiling control in DMF. 3) Bridging with 4 eq. of *N*-methyl-dibromomaleimide and stop after 1, 4) 5, 5) 10, 6) 20, 7) 40 and 8) 60 min. 9) Bridging with 20 eq. of *N*-methyl-dibromomaleimide and stop after 1, 10) 5, 11) 10, 12) 20, 13) 40 and 14) 60 min.

Figure S2 – Optimization of the *in situ* bridging protocol

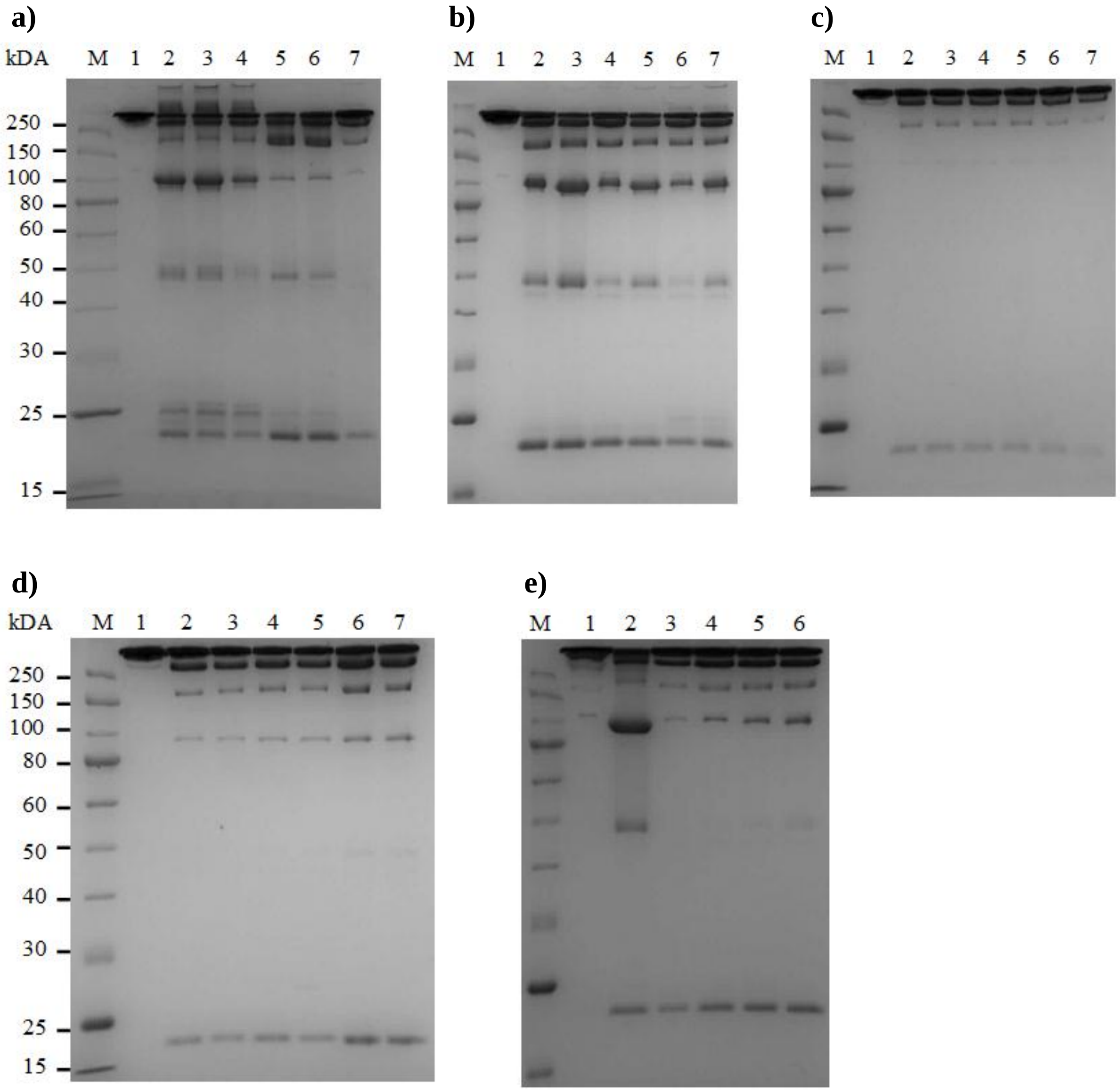

Figure S2 – Optimization of the *in situ* bridging of trastuzumab. All reactions were carried out as described for the general procedure with the here mentioned exceptions. **a)** Bridging at different temperatures in 20% DMF. The following ratios are eq. benzeneselenol: *N*-methyl-dithiophenolmaleimide. M) Molecular weight marker. 1) Untreated antibody. 2) 40 : 10, 3) 25 : 25 and 4) 10 : 40 at 37 °C. 5) 40 : 10, 6) 25 : 25 and 7) 10 : 40 on ice. **b)** Bridging in different amounts of DMF at 37 °C. The following ratios are eq. benzeneselenol : *N*-methyl-dithiophenolmaleimide. M) Molecular weight marker. 1) Untreated antibody. 2) 10 : 20 in 5% DMF. 3) 20 : 40 in 5% DMF. 4) 10 : 20 in 10% DMF. 5) 20 : 40 in 10% DMF. 6) 10 : 20 in

15% DMF. 7) 20 : 40 in 15% DMF. **c)** Bridging with different eq. of *N*-methyl-dithiophenolmaleimide in the presence of 10 eq. benzeneselenol on ice for 2 h. M) Molecular weight marker. 1) Untreated antibody. 2) 5, 3) 10, 4) 15, 5) 20, 6) 30 and 7) 40 eq. *N*-methyl-dithiophenolmaleimide. **d)** *In situ* bridging with TCEP. The following ratios are eq. TCEP : *N*-methyl-dithiophenolmaleimide employed for 2 h at 37 °C and 10% DMF. M) Molecular weight marker. 1) Untreated antibody. 2) 7 : 7. 3) 7 : 10. 4) 10 : 10. 5) 10 : 15. 6) 15 : 15. 7) 15 : 22. **e)** Repeated addition of benzeneselenol. The reaction was incubated for 60 min. in the presence of 20 eq. *N*-methyl-dithiophenolmaleimide and benzeneselenol was added in portions of 10 eq. M) Molecular weight marker. 2) Untreated antibody. 3) Representative sequentially bridged sample. 4) Addition of benzeneselenol at 0 min, 5) at 0 and 30 min, 6) at 0, 20 and 40 min and 7) at 0, 15, 30 and 45 min.

Figure S3 – UV-vis spectra for determination of DAR for ADCs prepared with NGM-DOX 3-5 and M-DOX 6 by sequential protocol

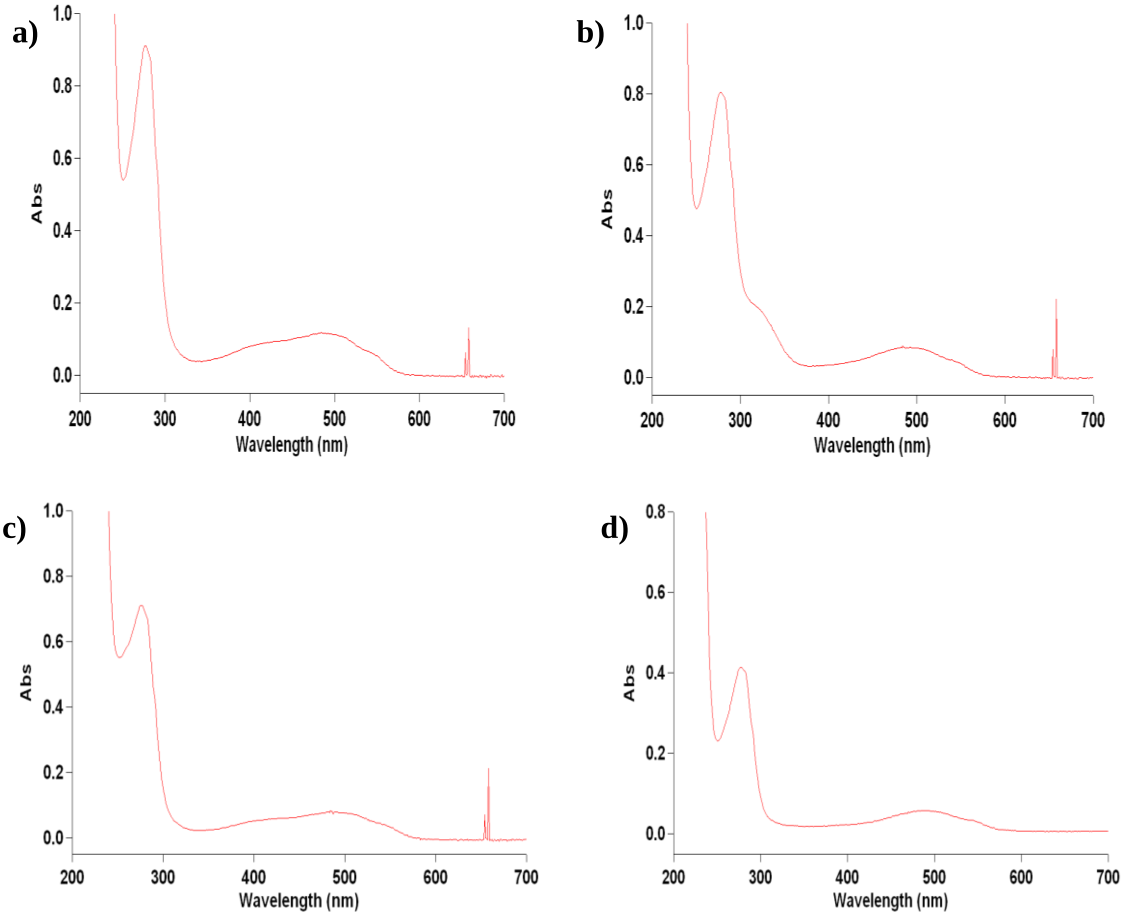

Figure S3 – UV-vis spectra for determination of DAR for ADCs prepared by sequential protocol with: **a)** NGM-DOX **3**, DAR 3.8. **b)** NGM-DOX **4**, DAR 3.0. **c)** NGM-DOX **5**, DAR 3.5. **d)** M-DOX **6**, DAR 3.7.

Table S1 – Ellman's analysis after conjugation of trastuzumab with NGM-DOX **3-5** by sequential protocol.

Entry	Reagent	DAR	Yield (%)	Ratio Reactive:mAb
1	no reagent	---	---	8.16
2	**3**	3.8	91	0.09
3	**4**	3.0	99	0.18
4	**5**	3.5	90	0.24

Notes: Reduction with TCEP (7 eq.). 37 °C, 2 h, followed by conjugation with NGM-DOX **3-5** (5 eq.), 37 °C, 1 h. Reactive corresponds to nucleophiles reactive towards Ellman's reagent, which includes any sulphydryl groups, traces of free thiophenol and TCEP.

Figure S4 – UV-vis spectra for determination of DAR for ADCs prepared with NGM-DOX 3-5 by *in situ* protocol

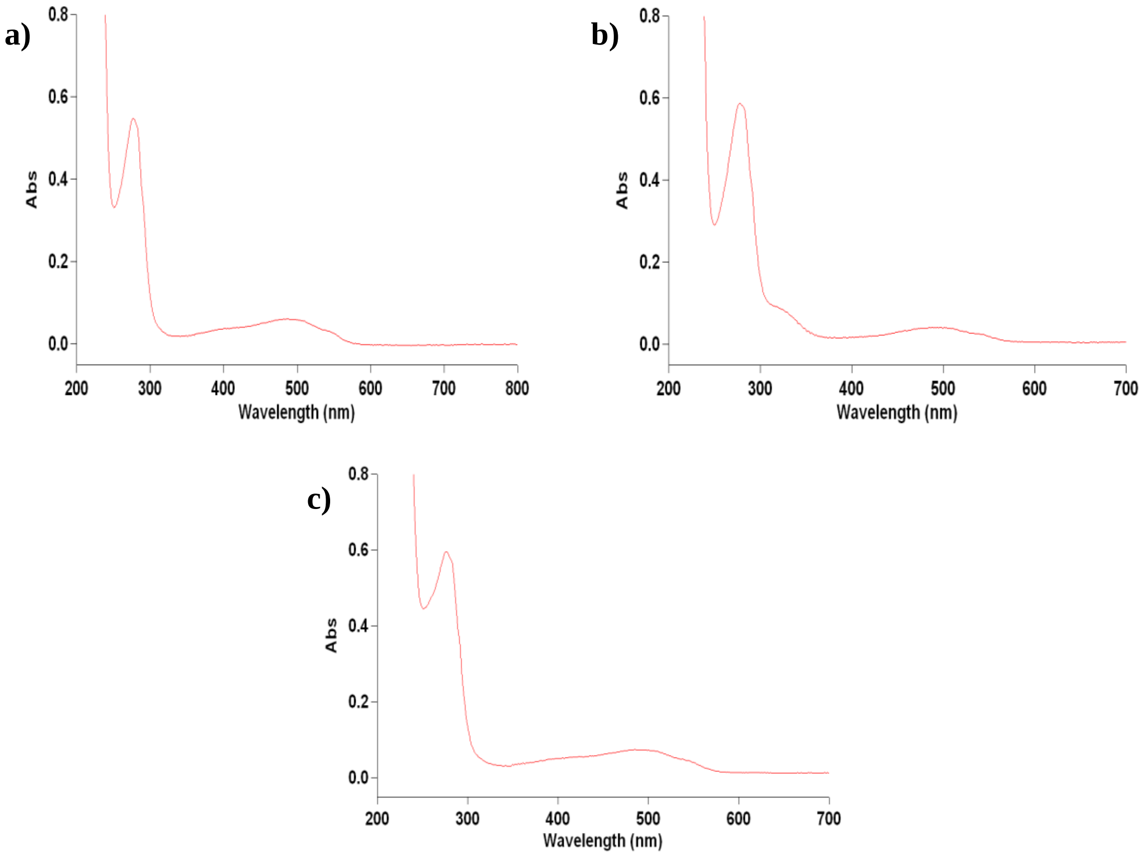

Figure S4 – UV-vis spectra for determination of DAR for ADCs prepared by *in situ* protocol with: **a)** NGM-DOX **3**, DAR 3.4. **b)** NGM-DOX **4**, DAR 1.8. **c)** NGM-DOX **5**, DAR 3.1.

Table S2 – Ellman's analysis after conjugation of trastuzumab with NGM-DOX **3-5** by *in situ* protocol.

Entry	Reagent	DAR	Yield (%)	Ratio Reactive:mAb
1	**3**	3.4	80	0.09
2	**4**	1.8	88	0.04
3	**5**	3.1	85	0.08

Notes: Reaction with NGM-DOX **3-5** (5 eq.) with concomitant reduction with TCEP (6 eq.), 37 °C, 2 h. Reactive corresponds to nucleophilic groups reactive towards Ellman's reagent, which would include any sulphydryl groups, traces of free thiophenol and TCEP.

Figure S5 – Reduction SDS-PAGE gel of ADCs to determine hydrolysis of maleimide bridges to maleamic acid bridges

Dithiothreitol (DTT) cleaves both native disulfides and maleimide bridges between cysteines but not maleamic acid derivate bridges, given that, as reported,[9] the latter are much more resistant to thiol attack. Thus, a trastuzumab ADC incubated with DTT would result in fragmentation if it has maleimide bridges and/or unmodified disulfide bonds, but experience no fragmentation if it has maleamic acid bridges. The trastuzumab ADCs (20 μM) prepared *via* sequential protocols with NGM-DOX **3-5** were subsequently incubated with DTT (100 eq.) in PBS (pH 7.4) at 37 °C for 2 h after which the samples were quenched with maleimide (1000 eq.). The ADC of trastuzumab with NGM-DOX **4** sustained no fragmentation, indicating that all maleimide bridges were already hydrolysed to maleamic acid derivate as a result of conjugation conditions (pH 8) On the other hand, ADCs conjugated with NGM-DOX **3** or **5** were cleaved upon incubation with DTT leaving free heavy and light chains as observed by SDS-PAGE gel (Figure S5).

a)

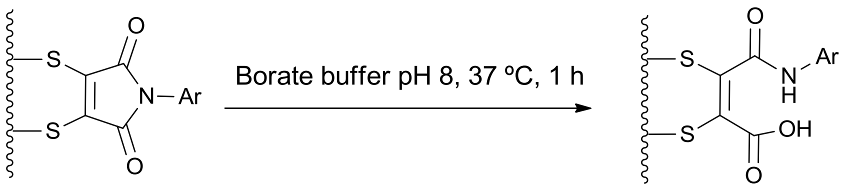

b)

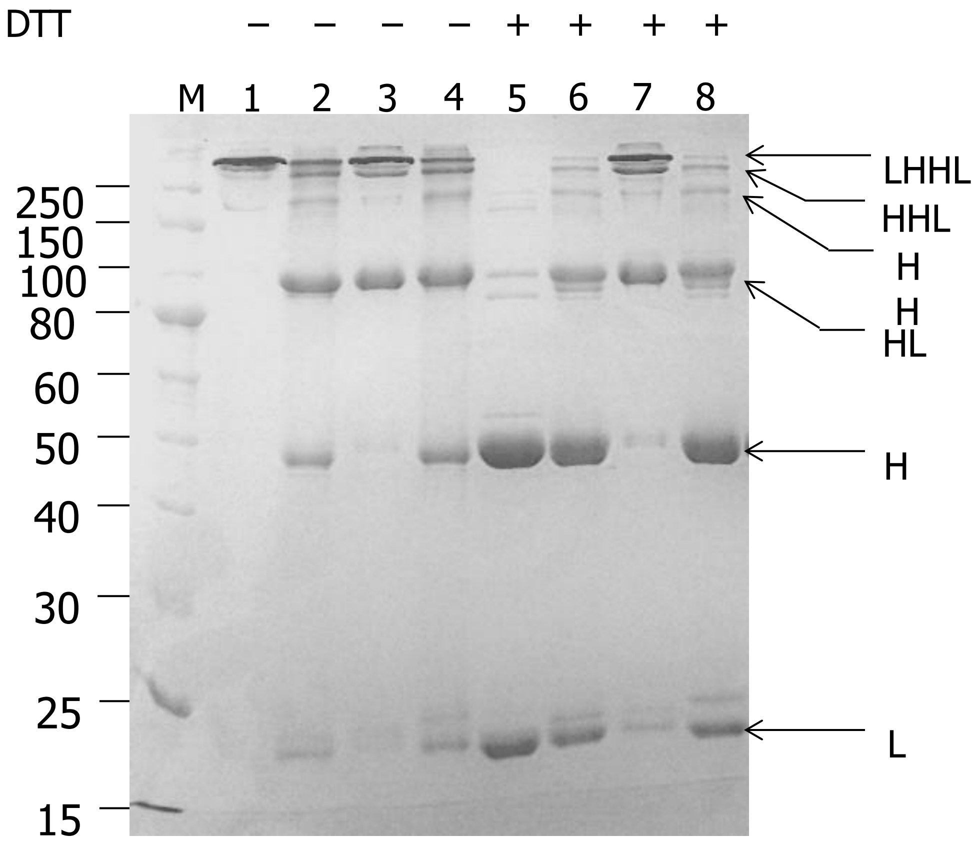

Figure S5 – **a)** Hydrolysis of maleimide bridges to maleamic acid bridges outline. **b)** SDS-PAGE gel of trastuzumab ADCs prepared by sequential method with NGM-DOX **3**-**5**, then incubated with DTT (100 eq.) at 37 °C for 4 h and quenched with maleimide (1000 eq.). M) protein ladder. 1) unmodified trastuzumab. 2) ADC with NGM-DOX **3** (no DTT). 3) ADC with NGM-DOX **4** (no DTT). 4) ADC with NGM-DOX **5** (no DTT). 5) reduced trastuzumab with DTT. 6) ADC with NGM-DOX **3** reduced with DTT. 7) ADC with NGM-DOX **4** reduced with DTT. 8) ADC with NGM-DOX **5** reduced with DTT.

Figure S6 – **MS data of conjugation of trastuzumab Fab with NGM-DOX 4**

Trastuzumab Fab was prepared through a reported protocol[10] and modified by an adaptation of the sequential and *in situ* protocols described above, using TCEP (3 eq.) and NGM-DOX **5** (5 eq.) in borate buffer at 37 °C. Analysis by LCMS revealed that all Fab was fully bridged as a mixture of the expected Fab-DOX conjugate (expected mass: 48441) and the product resulting from loss of the anthracycline motif of DOX (expected mass 48045) (Figure S6).

This instability of DOX has been previously reported.[10, 11] Also, DAR values measured by UV-vis absorbance are in agreement with LCMS analysis, indicating that this is the likely cause for low DAR for conjugation with NGM-DOX **4** (Table S3).

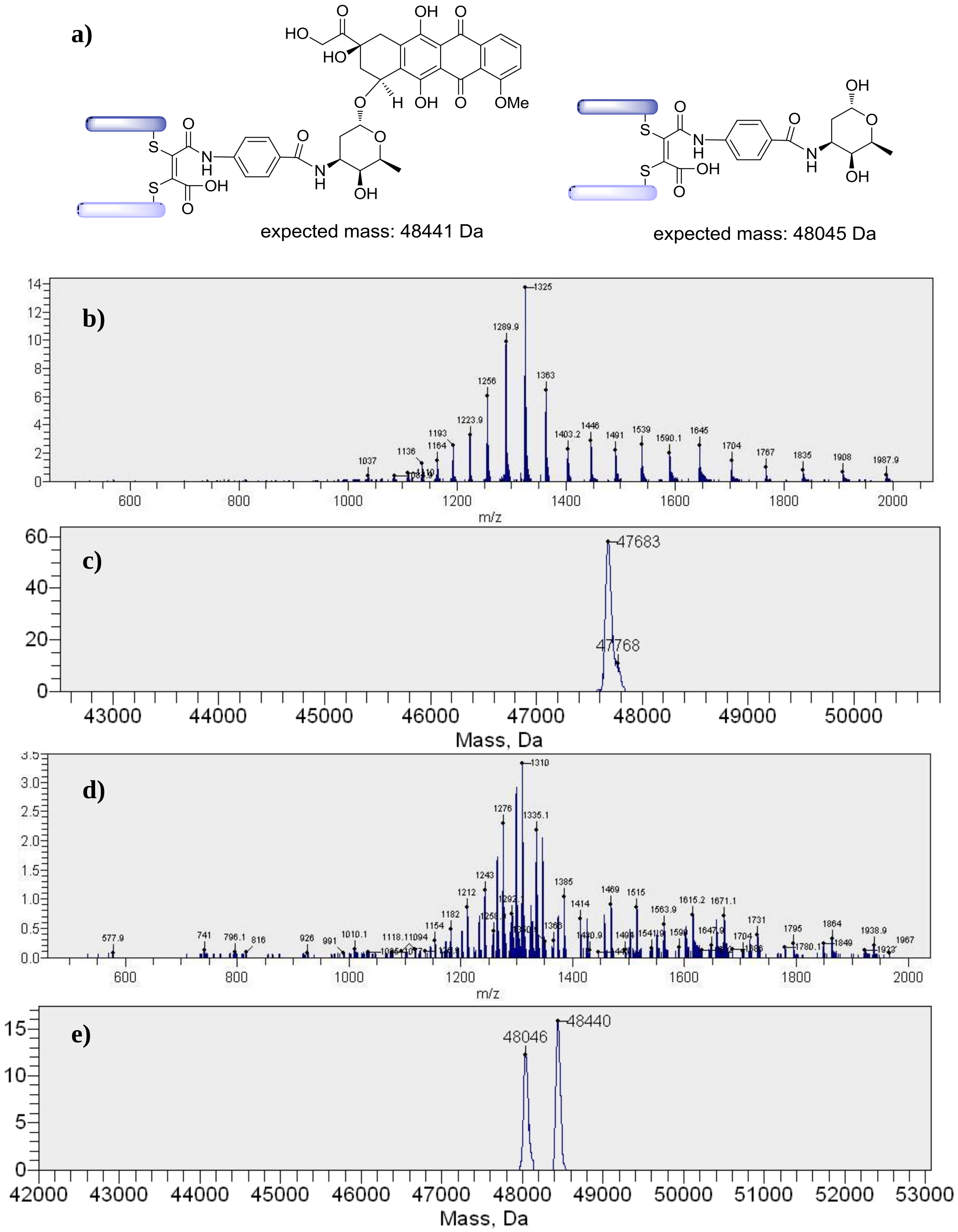

Figure S6 – ESI-MS data for the sequential conjugation of trastuzumab Fab with NGM-DOX **4** (5 eq.). **a)** Expected masses for the observed hydrolysed products (based on the observed Fab mass of 47683 Da). **b)** Non-deconvoluted and **c)** deconvoluted mass data for the unmodified Fab fragment (expected mass: 47685 Da). **d)** Non-deconvoluted and **e)** deconvoluted mass data for the Fab-DOX conjugate.

Table S3 – Comparison of DAR by UV-vis and amount of Fab-DOX by LCMS for conjugation of trastuzumab Fab with NGM-DOX **4**.

Entry	Protocol	NGM-DOX 5 (eq.)	DAR	Fab ADC by LCMS (%)
1	Sequential	5	0.64	67
2	*In situ*	5	0.74	77

Notes: DAR was determined by UV-vis absorbance. Fab ADC by LCMS (%) corresponds to the full ADC of trastuzumab Fab with intact DOX payload (expected mass: 48441 Da). The indicated value corresponds to the respective peak area by LCMS expressed as a percentage of the sum of all peaks.

Figure S7 – Initial bridging with compound 15 and influence of duration of the copper-catalysed Huisgen 1,3-dipolar cycloaddition

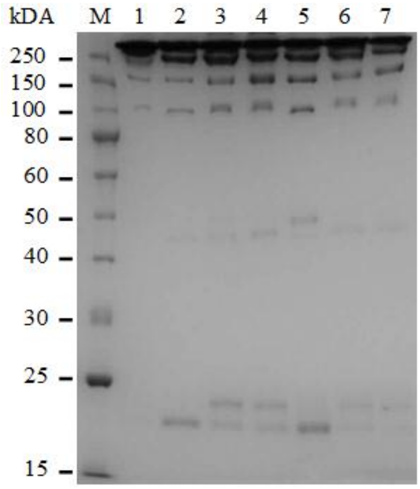

Figure S7 – Influence of *N*-alkyne-dithiophenolmaleimide **15** bridging and duration of the copper-catalysed Huisgen 1,3-dipolar cycloaddition on the preparation of ADCs using NGMs. Trastuzumab was modified using the pre-optimized conditions as described followed by copper-catalysed Huisgen 1,3-dipolar cycloaddition as described for various reaction times. M) Molecular weight marker. 1) Untreated antibody. 2) Antibody bridged using the

benzeneselenol-based protocol before the click reaction, 3) after 90 min click reaction (DAR 1.1) and 4) after 16 h click reaction (DAR 1.7). 5) Antibody bridged using the TCEP-based protocol before the click reaction, 6) after 90 min click reaction (DAR 3.1) and 7) after 16 h click reaction (DAR 4.0).

Figure S8 – UV-vis spectra for determination of DAR for ADCs prepared with compound 15 by *in situ* protocols, followed by copper-catalysed Huisgen 1,3-dipolar cycloaddition with compound 16

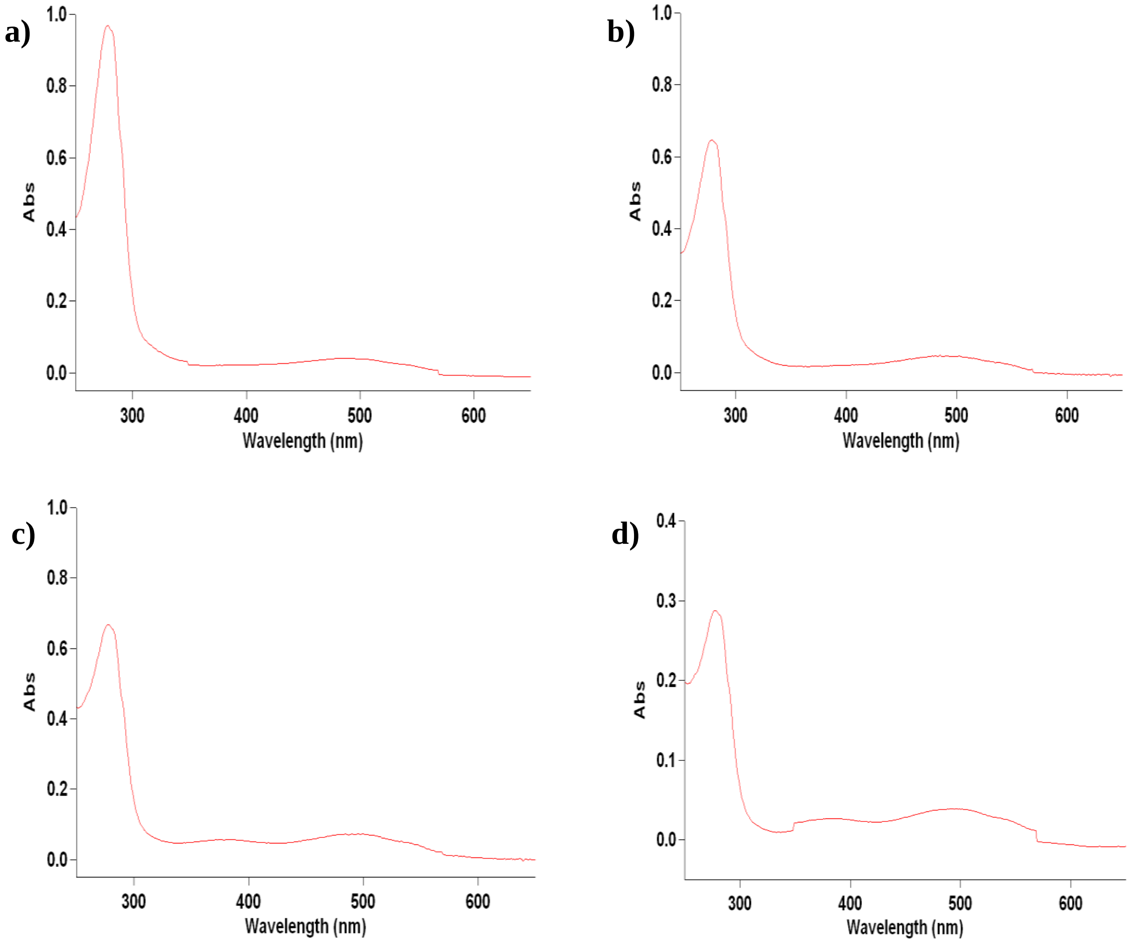

Figure S8 – UV-vis spectra for determination of DAR for ADCs prepared by *in situ* protocols with NGM **15**, followed by cycloaddition with compound **16** for DARs of: **a)** 1.1. **b)** 2.0. **c)** 3.1. **d)** 4.0.

Figure S9 – UV-vis spectra for determination of DAR for trastuzumab Fab ADC resulting from proteolytic digest of DAR 2 ADC material

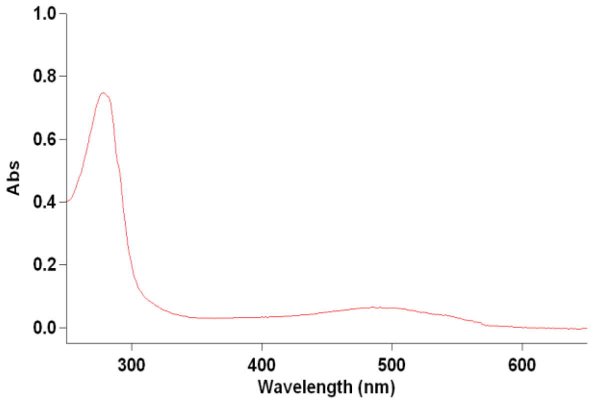

Figure S9 – UV-vis spectra for determination of DAR for trastuzumab Fab ADC with DAR of 0.79, resulting from proteolytic digest of DAR 2 ADC material prepared by *in situ* protocol with NGM **15**, followed by cycloaddition with compound **16**.

Figure S10 – MS data on the control digest of DAR 2 material

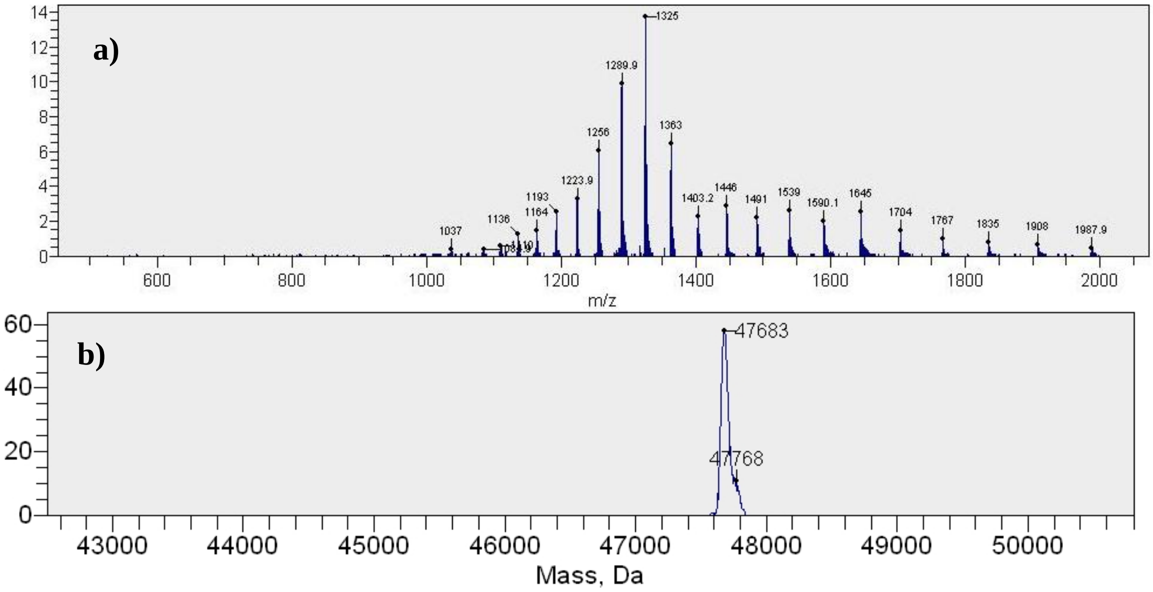

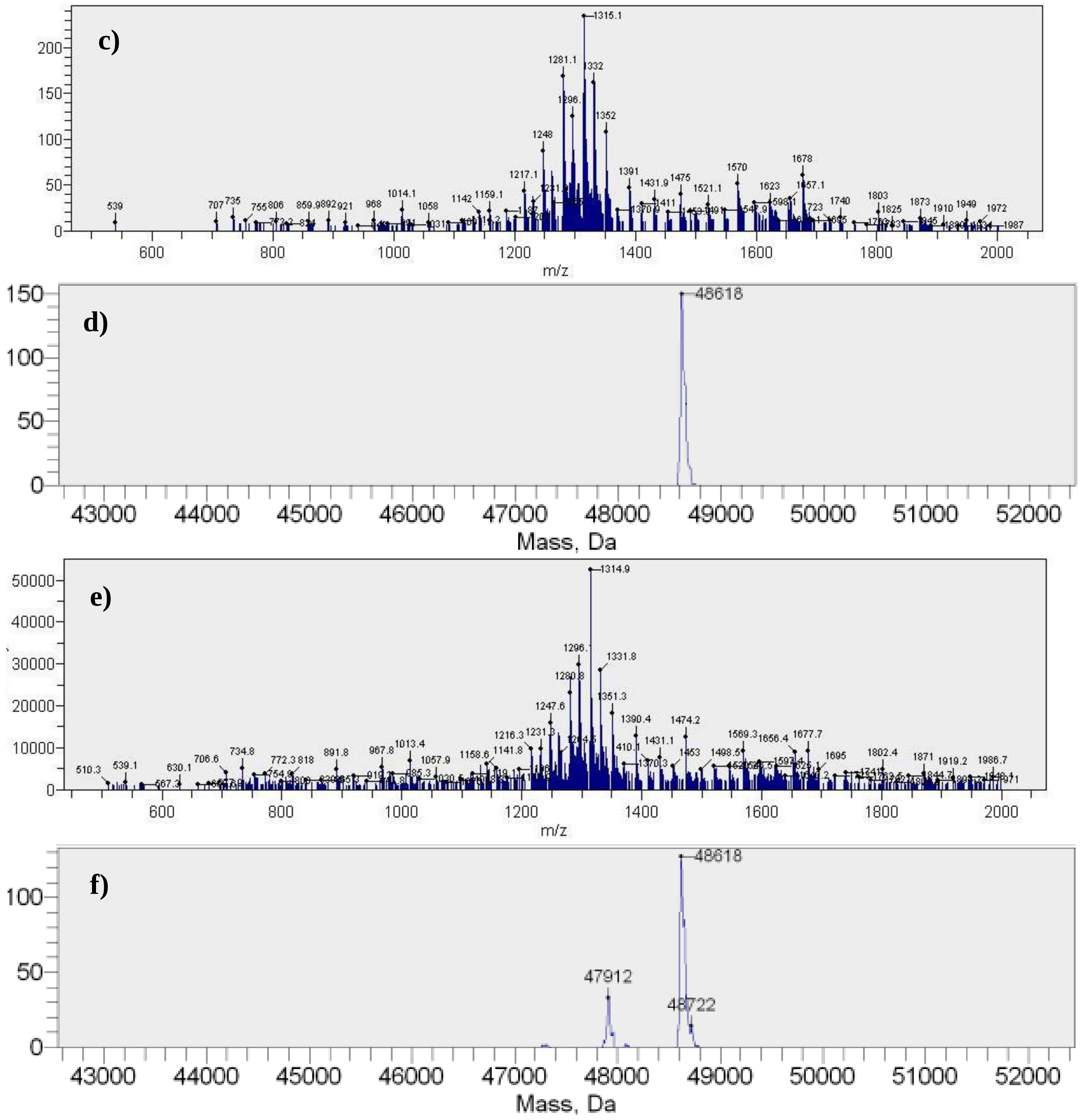

Figure S10 – ESI-MS data of the control experiment of the DAR 2 digest. **a)** Unmodified Fab fragment (expected mass: 47685 Da). **b)** Non-deconvoluted and **c)** deconvoluted mass data for the unmodified Fab fragment (expected mass: 47685 Da). **d**) Non-deconvoluted and **e)** deconvoluted mass data for Fab-DOX conjugate after bridging and click reaction (expected mass: 48619 Da). **e**) Non-deconvoluted and **f)** deconvoluted mass data for Fab-DOX conjugate after digest treatment.

Figure S11 – UV-vis spectra for determination of DAR for the control digest of DAR 2 material

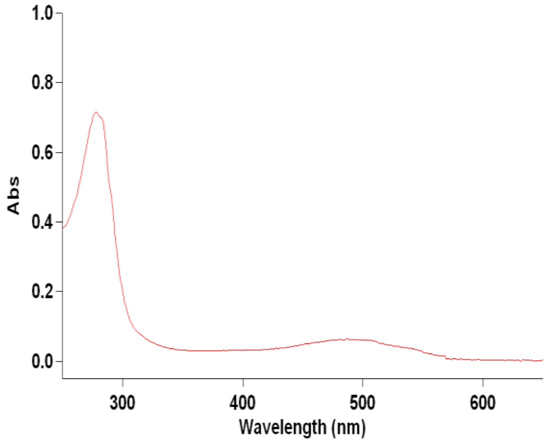

Figure S11 – UV-vis spectra for determination of DAR for trastuzumab Fab ADC with DAR 0.8. Trastuzumab Fab ADC was prepared from trastuzumab Fab by *in situ* protocol with NGM **15**, followed by cycloaddition with compound **16** and subjected to proteolytic digest conditions to serve as control digest of DAR 2 material.

References

1. F. F. Schumacher, M. Nobles, C. P. Ryan, M. E. B. Smith, A. Tinker, S. Caddick and J. R. Baker, *Bioconjugate Chem.*, 2011, **22**, 132-136.
2. M. Dubernet, V. Caubert, J. r. m. Guillard and M.-C. Viaud-Massuard, *Tetrahedron*, 2005, **61**, 4585-4593.
3. O. Keller and J. Rudinger, *Helv. Chim. Acta*, 1975, **58**, 531-541.
4. D. H. Rich, P. D. Gesellchen, A. Tong, A. Cheung and C. K. Buckner, *J. Med. Chem.*, 1975, **18**, 1004-1010.
5. G. M. Dubowchik, R. A. Firestone, L. Padilla, D. Willner, S. J. Hofstead, K. Mosure, J. O. Knipe, S. J. Lasch and P. A. Trail, *Bioconjugate Chem.*, 2002, **13**, 855-869.
6. L. Castañeda, Z. V. F. Wright, C. Marculescu, T. M. Tran, V. Chudasama, A. Maruani, E. A. Hull, J. P. M. Nunes, R. J. Fitzmaurice, M. E. B. Smith, L. H. Jones, S. Caddick and J. R. Baker, *Tetrahedron Lett.*, 2013, **54**, 3493-3495.
7. A. W. Schwabacher, J. W. Lane, M. W. Schiesher, K. M. Leigh and C. W. Johnson, *J. Org. Chem.*, 1998, **63**, 1727-1729.
8. G. Candiano, M. Bruschi, L. Musante, L. Santucci, G. M. Ghiggeri, B. Carnemolla, P. Orecchia, L. Zardi and P. G. Righetti, *Electrophoresis*, 2004, **25**, 1327-1333.
9. C. P. Ryan, M. E. B. Smith, F. F. Schumacher, D. Grohmann, D. Papaioannou, G. Waksman, F. Werner, J. R. Baker and S. Caddick, *Chem. Commun.*, 2011, **47**, 5452-5454.
10. L. Castañeda, A. Maruani, F. F. Schumacher, E. Miranda, V. Chudasama, K. A. Chester, J. R. Baker, M. E. B. Smith and S. Caddick, *Chem. Commun.*, 2013, **49**, 8187-8189.
11. L. A. Trissel, in *Handbook on Injectable Drugs*, ed. American Society of Health-System Pharmacists, MacMillan Press, Basingstoke, 9th edn., 1996, 379.
